# Global burden, trends and projections analysis of interstitial lung disease and pulmonary sarcoidosis in elderly adults (aged 55+ Years) based on GBD 2021

**DOI:** 10.1371/journal.pone.0347482

**Published:** 2026-04-20

**Authors:** Zhilan Huang, Tingyi Xie, Mingwen Tang, Zhuni Chen, Dan Jia, Anqi Su, Zhujin Jin, Tuliang Liang, Wei Xie

**Affiliations:** 1 The Fourth Clinical Medical College of Guangzhou University of Chinese Medicine, Shenzhen, Guangdong, China; 2 Department of Respiratory Medicine, Shenzhen Traditional Chinese Medicine Hospital, Shenzhen, China; Kurume University School of Medicine: Kurume Daigaku Igakubu Daigakuin Igaku Kenkyuka, JAPAN

## Abstract

**Background:**

Pulmonary fibrosis is a severe chronic lung disease whose prevalence has been rising in recent years, representing one of the major respiratory health challenges globally in the 21st century. The burden of this disease on the elderly population is garnering growing attention, particularly as the global population ages. The Global Burden of Disease (GBD) study has provided valuable insights; however, systematic analyses focused on this condition remain limited. To date, few studies have specifically examined interstitial lung disease and pulmonary sarcoidosis among individuals aged 55 years and older. This study aims to conduct a comprehensive analysis of burden trends from 1990 to 2021 for those aged 55 and above and to project future trends up to 2035.

**Methods:**

Our approach utilizes the estimation of four broad component measures: incidence, prevalence, death and Disability-Adjusted Life Years (DALYs), using data on ILD&PS from the Global Burden of Disease (GBD) 2021 database. Joinpoint regression models were applied to calculate the average annual percentage change (AAPC) in order to analyze temporal trends in disease burden and to identify years with significant trend shifts. Analyses were further stratified by age, sex, region, country, and Sociodemographic Index (SDI). Additionally, a Bayesian age-period-cohort (BAPC) model was used to project future disease burden trends.

**Results:**

Between 1990 and 2021, significant increases were observed in incidence, DALYs, and death rates for ILD&PS (AAPC incidence = 1.09, 95% CI: 1.04 to 1.15; AAPC DALYs = 1.10, 95% CI: 0.97 to 1.23; AAPC death = 1.65, 95% CI: 1.47 to 1.83). In 2021, the total number of incident cases reached 284,887 (95% UI 248,300–328,800), with the highest incidence rates observed in Andean Latin America. Across age- and sex-specific analyses, global burden trends were similar, though males consistently exhibited higher rates than females. The oldest age group (95 + years) had the highest incidence and DALYs rates among all age strata. Furthermore, incidence rates increased most markedly in high-SDI regions, showing a strong positive correlation between SDI and incidence. Bayesian age–period–cohort (BAPC) analyses indicated that while prevalence rates are projected to decline slightly, incidence rates are expected to continue rising. Both males and females showed a dip then rise in prevalence trends, but the increase was more pronounced among females. In 2035, the highest number of incident cases is projected to occur in the 65–69 age group, whereas the highest incidence rate is predicted in the 95 + age group.

**Conclusions:**

A concerning upward trend in incidence, DALYs, and deaths related to ILD&PS was observed in the global population aged 55 years and older, particularly among females. To our knowledge, this is the first study to comprehensively analyze the burden of ILD&PS in this age group from 1990 to 2021. Our findings on epidemiological trends and their variations across geography, SDI, age, and sex can inform policy-makers in designing targeted strategies to mitigate the anticipated rise in disease burden.

## Introduction

Interstitial Lung Disease (ILD) encompasses a spectrum of chronic, progressive lung disorders comprising over 200 distinct pathological types. The common characteristic of these diseases is inflammation and fibrosis of the alveolar walls and interstitium [1,2]. Pulmonary sarcoidosis, a systemic granulomatous disease, is one of the primary forms of ILD, with the lungs being a major target organ [3,4]. Research has characterized pulmonary sarcoidosis as an autoimmune disorder in which T lymphocytes initiate an immune response against self‑proteins through interactions with innate immune system cells during the differentiation phase [5]. Additionally, 10% to 20% of patients with pulmonary sarcoidosis may progress to fibrotic pulmonary sarcoidosis. This progression can lead to a decline in quality of life and, in severe cases, become life-threatening**.** The mortality rate in advanced stages is approximately 7% within a five-year follow-up period [6,7]. The clinical manifestations of ILD are typically non‑specific, with common symptoms including dyspnea and reduced exercise tolerance. Pulmonary function mainly exhibits restrictive ventilatory impairment and decreased gas exchange capacity, both of which can significantly affect quality of life or lead to severe respiratory failure [2,8]. Common ILDs encountered in clinical practice include Idiopathic Pulmonary Fibrosis (IPF), Hypersensitivity Pneumonitis (HP), and Connective Tissue Disease-associated ILD (CTD-ILD) [9]. Among these, IPF is the most prevalent and severe type, with a median survival of only 2–3 years [10].

Previous evidence has demonstrated a significant correlation between age and the incidence of IPF, with the incidence increasing with advancing age. The average age at diagnosis of IPF is approximately 65 years. However, certain subtypes of ILD may occur at an earlier age. Globally, there has been a notable rise in the patient population, likely attributable to factors such as population aging**,** increased awareness of the disease, and advancements in diagnostic methods [11].

Currently, the prevailing view is that pulmonary fibrosis results from scar repair driven by injured epithelial cells, leading to epithelial cell dysfunction and mesenchymal transformation [12]. Therapeutic options for ILD include immunomodulatory agents, treatments for vasculopathy, and anti-fibrotic therapies. Evidence-based guidelines for the treatment of IPF recommend the use of either nintedanib or pirfenidone. Both medications have been shown to slow the decline in forced vital capacity (FVC) and diffusing capacity of the lungs for carbon monoxide (DLCO), thereby helping delay disease progression and reducing the risk of acute exacerbations [13,14]. Lung transplantation remains the only intervention proven to extend life expectancy in patients with IPF. Among postoperative ILD patients, the median survival improves to 4.7 years; however, this procedure is associated with an increased risk of thromboembolism [15,16].

Epidemiological data indicate that the global incidence and prevalence of ILD vary significantly by region, age, sex, and race [11], but generally show an upward trend. U.S. National Death Statistics (NDS) indicate an increase in the overall age-adjusted mortality rate for idiopathic pulmonary fibrosis (IPF) from 18.81 to 20.66 per 100,000 population between 2002 and 2017 [17]. Similarly, the UK Office for National Statistics (ONS) data demonstrated a rise in the annual IPF mortality rate from 1.66 per 100,000 to 8.29 per 100,000 population from 1979 to 2016 [18].

The Global Burden of Disease (GBD) study provides a comprehensive platform for assessing the prevalence and health impacts of various diseases worldwide [19]. GBD 2021 summarizes cause-specific mortality and years of life lost (YLLs) for 288 causes, stratified by age and sex across 204 countries and territories from 1990 to 2021 [20]. Globally, ILD&PS is the third leading cause of death from chronic respiratory diseases, following asthma, and ranked as the third largest contributor to disability-adjusted life years (DALYs) in 2021 compared to 1990. Therefore, quantifying the burden of ILD&PS is essential to improving our understanding of disease incidence and evaluating progress toward health targets. While previous studies have examined global incidence, mortality, and DALYs from 1990 to 2019 [21], as well as projected trends through 2030 [22], our analysis specifically focuses on individuals aged 55 years and older. The selection of the lower age limit of 55 years is based on its common use as a threshold to define the “older adult” population in many global studies on aging and chronic diseases. This age group often represents a cohort at increased risk for chronic respiratory conditions due to cumulative environmental exposures, age-related declines in physiological function, and a higher prevalence of comorbidities [23]. This demographic represents a growing proportion of the global population amid worldwide aging trends. Additionally, long-term exposure to environmental risk factors, multimorbidity, and natural physiological decline contribute to a higher risk of disease in this age group. Furthermore, respiratory function declines markedly with age, and the weakening of the immune system results in reduced inhibition of inflammation and a higher risk of respiratory infections. Consequently, older adults may be more susceptible to ILD. Thus, the disease burden among the elderly poses a distinct challenge to public health systems, necessitating targeted preventive and therapeutic strategies.

Our study has three main aims: (a) to evaluate the most current estimates of incidence, prevalence, DALYs, and mortality rates; (b) to conduct a stratified analysis of global trends by age group, sex, and Socio-demographic Index (SDI); and (c) to forecast future epidemiological trajectories up to 2035. We primarily utilize GBD 2021 data to retrospectively describe trends in ILD&PS worldwide from 1990 to 2021 among individuals aged 55 years and older. This analysis provides an updated basis for further epidemiological research, disease prevention, and the formulation of public health policies.

## Methods

### Reference and data sources

In our analysis, we used cross-sectional data obtained from GBD 2021 data, available at the Global Health Data Exchange (GHDx) platform (https://vizhub.healthdata.org/gbd-results/). This dataset encompasses the latest estimates of descriptive epidemiologic data for the global burden of 371 diseases and injuries across 204 countries and territories from 1990 to 2021. Specifically, we collected data for interstitial lung disease and pulmonary sarcoidosis within the 55 + age group. In this report, since the potential effects of risk factors for ILD&PS on incidence burden are not represented in the current GBD study, the population attributable fraction estimation in the 55 + age group was not discussed.

### Disease definition

For our analysis, data specific to interstitial lung disease and pulmonary sarcoidosis were extracted. Based on the GBD 2021 classification, both ILD&PS are grouped together as a single cause of death or injury. The diagnosis of ILD&PS adheres to the International Statistical Classification of Diseases and Related Health Problems, 10th Revision (ICD-10; https://icd.who.int/browse10/2019/en), encompassing the following codes: J84 (Interstitial pulmonary diseases), including J84.0 (Alveolar and parietoalveolar conditions), J84.1 (Other interstitial pulmonary diseases with fibrosis), J84.8 (Other specified interstitial pulmonary diseases), and J84.9 (Interstitial pulmonary disease, unspecified), as well as J86.0 (Pulmonary nodular disease). Although pneumoconiosis is clinically classified as an interstitial lung disease, GBD 2021 categorizes it as a distinct cause of death or injury. Therefore, data on pneumoconiosis from GBD were not included in our analytical dataset.

### Study population

People over the age of 55 were included in our analysis and divided into nine age strata (55–59,60–64,65–69,70–74,75–79,80–84,85–89,90–94,95+). We used the GBD results tool to extract estimates of deaths, prevalence, incidence, disability-adjusted life years (DALYs), and their rates were reported with 95% uncertainty intervals (UIs), whose definition and methodology employed by GBD 2021 are documented in previously published associated literature [24]. The burden of elderly ILD&PS was analyzed by sex, year, age, and locations, along with 204 countries and territories. And it is divided into 21 GBD regions based on geographic proximity which were categorized into quintiles based on sociodemographic index (SDI) level: low, low-middle, middle, high-middle, and high to measure the burden for the period 1990–2021 across regions and countries.

### SDI

The Sociodemographic Index (SDI) is a composite indicator of a country or region’s level of development. It is derived as the geometric mean of three normalized components: the total fertility rate among women under 25 years of age, the average years of schooling in the population aged 15 and above, and the lag‑distributed income per capita [25]. The index ranges from 0 to 1, with values positively correlated with income and years of schooling, and negatively correlated with fertility. In other words, a score of 0 corresponds to the lowest income, fewest years of schooling, and highest fertility, while a score of 1 corresponds to the opposite [19]. Based on their SDI scores, regions are classified into five quintiles: low, low‑middle, middle, high‑middle, and high.

### Statistical analysis

a) Global Trend (1990–2021): The study aimed to assess global trends in the four main measures — incidence, prevalence, DALYs, and deaths — of ILD&PS from 1990 to 2021. Age-specific rates and average annual percentage changes (AAPCs) were used to track trends over time. The AAPC was calculated using a linear regression of the logarithm of the rates and is a weighted average representing the annual percentage change. The AAPC value indicates the overall annual rate of change (increasing, decreasing, or stable). For instance, an AAPC value of 0.1 corresponds to an average annual growth rate of 0.1%. AAPC values are reported along with their 95% confidence intervals (CIs). A positive AAPC indicates an increasing trend in incidence, death, or DALYs rates, while a negative AAPC suggests a decline.
b) Joinpoint Regression Analysis: We performed joinpoint regression analysis to identify years with significant changes in trends. This method estimates the annual percentage change (APC) within each segment defined by these joinpoints, thereby helping to distinguish meaningful shifts from random variability. We fit a linear regression model on the logarithm of each indicator, with year as the independent variable, allowing for a maximum of five joinpoints. This analysis produced APC estimates for each segment, along with corresponding 95% confidence intervals (CIs), providing insight into the direction and magnitude of trends throughout the study period.
c) Stratified Trends and Correlation Analysis: Trends were analyzed after stratification by age, sex, and SDI. Additionally, we examined the correlation between SDI and the four burden indicators using Pearson correlation analysis, assessing the strength and direction of their linear relationship across regions and all 204 countries.
d) Prediction: A Bayesian age-period-cohort (BAPC) model was used to forecast future disease burden from 2022 to 2035. Prior to forecasting, retrospective validation was performed by fitting the model using data from 1990–2016 and predicting prevalence for 2017–2021 to evaluate the predictive performance of the BAPC model. Model performance is quantitatively evaluated by comparing predicted values with observed values using three metrics: mean absolute error (MAE), which measures the average magnitude of the predicted error; root mean square error (RMSE), which calculates the square root of the mean squared error; and mean absolute percentage error (MAPE), which measures the average percentage deviation of the predicted value from the observed value. 95% Uncertainty Interval (UI) coverage, which determines the percentage of observations that fall within the 95% prediction interval. And then the BAPC model estimated the number of cases and disease prevalence by 2035, incorporating projected future population counts and sociodemographic indices (SDIs) as predictors. By combining past data with probability distributions, the model accounts for age, period, and cohort effects, thus capturing potential patterns and dynamics in disease burden over time and enabling predictions of the changing epidemiologic landscape of ILD&PS [26,27].

In the current study, all statistical analyses and visualizations were conducted using R version 4.4.1 [R Core Team (2021)], Origin 2021, and the online data analysis and visualization platform https://www.bioinformatics.com.cn (last accessed on 10 October 2024).

## Results

### Global trends

The incident case counts and incidence rates are summarized in Table 1 and S1 Fig. In 2021, an estimated 284,887 (95% UI: 244,836–328,846) cases were identified among the global population aged 55 years and older, accounting for 73% of the total incident cases across all age groups worldwide. The incidence rate increased from 13.75 per 100,000 in 1990 to 19.17 per 100,000 in 2021, representing a rise of 5.42%. Additionally, Table 1 present data indicating that ILD&PS resulted in 3,314,679 (95% UI: 2,897,474–3,787,334) prevalent cases, 174,368 (95% UI: 149,929–196,275) deaths, and 3,284,254 (95% UI: 2,847,979–3,664,739) DALYs within this age group. All of these metrics exhibited significant changes over the period from 1990 to 2021.

**Table 1:** Global Incidence and DALYs of ILD&PS and their AAPCs globally by gender, specific age group, SDI, and region from 1990 to 2021.

	Incidence						DALYs (Disability–Adjusted Life Years)					
	1990	1990	2021	2021	1990–2021	*P* value	1990	1990	2021	2021	1990–2021	*P* value
	Cases (n)	Rate (per 100 000 population)	Cases (n)	Rate (per 100 000 population)	AAPC (95%)		Cases (n)	Rate (per 100 000 population)	Cases (n)	Rate (per 100 000 population)	AAPC (95%)	
Global	92306 (75477–110535)	13.75 (11.24–16.46)	284887 (244836–328846)	19.17 (16.48–22.13)	1.09 (1.04 to 1.15)	0	1056321 (877668–1265644)	157.33 (130.72–188.50)	3284254 (2847979–3664739)	221.02 (191.66–246.62)	1.10 (0.97 to 1.23)	0
Sex												
Female	39182 (31877–47225)	10.89 (8.86–13.12)	122621 (104574–142311)	15.59 (13.30–18.09)	1.17 (1.14 to 1.21)	0	451128 (356958–604429)	125.33 (99.17–167.92)	1466042 (1202312–1798697)	186.41 (152.87–228.71)	1.28 (1.14 to 1.44)	0
Male	53124 (43543–63594)	17.06 (13.98–20.42)	162265 (140156–186817)	23.20 (20.04–26.71)	1.01 (0.97 to 1.04)	0	605194 (466976–746090)	194.29 (149.92–239.53)	1818212 (1500259–2067519)	259.92 (214.47–295.56)	0.98 (0.85 to 1.10)	0
Age group												
55–59 years	17969 (10982–26057)	9.70 (5.93–14.07)	40477 (25781–57982)	10.23 (6.51–14.65)	0.17 (0.09 to 0.24)	0	151442 (116197–195311)	81.77 (62.74–105.46)	330652 (266122–388740)	83.56 (67.25–98.23)	0.09 (–0.14 to 0.32)	0.452
60–64 years	20156 (12391–29666)	12.55 (7.71–18.47)	48087 (30736–68067)	15.02 (9.60–21.27)	0.58 (0.51 to 0.65)	0	195518 (152620–252684)	121.74 (95.03–157.33)	419878 (344894–501940)	131.19 (107.76–156.83)	0.27 (–0.05 to 0.60)	0.101
65–69 yearst	17909 (11597–26768)	14.49 (9.38–21.66)	52601 (34568–74257)	19.07 (12.53–26.92)	0.88 (0.79 to 0.97)	0	206531 (165763–253521)	167.08 (134.10–205.10)	522370 (434192–604073)	189.37 (157.41–218.99)	0.44 (0.22 to 0.67)	0
70–74 years	14063 (8521–20085)	16.61 (10.06–23.72)	52707 (33629–72823)	25.61 (16.34–35.38)	1.41 (1.25 to 1.58)	0	184848 (149901–230258)	218.34 (177.06–271.98)	610775 (514814–715439)	296.72 (250.10–347.57)	1.02 (0.79 to 1.25)	0
75–79 years	10336 (6767–14823)	16.79 (10.99–24.08)	37702 (25772–51769)	28.59 (19.54–39.25)	1.74 (1.65 to 1.82)	0	159646 (136680–191156)	259.35 (222.04–310.54)	538845 (470899–607655)	408.57 (357.05–460.75)	1.50 (1.21 to 1.78)	0
80–84 years	6654 (4472–9758)	18.81 (12.64–27.58)	26370 (18236–38195)	30.11 (20.82–43.61)	1.53 (1.48 to 1.58)	0	97168 (83256–114807)	274.67 (235.35–324.53)	422882 (360009–483633)	482.84 (411.05–552.20)	1.86 (1.68 to 2.04)	0
85–89 years	3592 (2733–4687)	23.77 (18.08–31.01)	16722 (13178–21243)	36.57 (28.82–46.46)	1.41 (1.37 to 1.44)	0	43762 (38233–50032)	289.60 (253.01–331.10)	265666 (222273–298320)	581.05 (486.14–652.47)	2.27 (1.90 to 2.65)	0
90–94 years	1264 (915–1752)	29.49 (21.35–40.89)	7606 (5645–10267)	42.52 (31.56–57.39)	1.20 (1.16 to 1.24)	0	13796 (11512–15856)	321.95 (268.64–370.02)	127031 (100800–144301)	710.09 (563.47–806.63)	2.64 (2.42 to 2.85)	0
95+ years	362 (231–521)	35.51 (22.64–51.20)	2615 (1745–3738)	47.97 (32.02–68.59)	0.98 (0.92 to 1.03)	0	3609 (2838–4212)	354.48 (278.78–413.75)	46154 (33944–53419)	846.82 (622.80–980.11)	2.84 (2.52 to 3.16)	0
Sociodemographic index (SDI)												
High–middle SDI	13288 (10903–15919)	7.70 (6.32–9.23)	39660 (34337–45513)	11.44 (9.90–13.13)	1.27 (1.14 to 1.39)	0	160247 (146909–178869)	92.88 (85.15–103.68)	403220 (355242–447809)	116.31 (102.47–129.17)	0.75 (0.46 to 1.05)	0
High SDI	42060 (34634–50187)	22.56 (18.57–26.92)	126795 (109606–145772)	36.75 (31.77–42.25)	1.60 (1.52 to 1.67)	0	397502 (368467–426929)	213.18 (197.61–228.96)	1349954 (1211439–1452156)	391.28 (351.13–420.90)	2.00(1.75 to 2.25)	0
Low–middle SDI	16958 (13597–20432)	16.82 (13.49–20.27)	46914 (39375–55120)	19.46 (16.33–22.86)	0.47 (0.43 to 0.51)	0	249378 (148403–376430)	247.40 (147.22–373.44)	718873 (493928–975084)	298.18 (204.88–404.46)	0.65 (0.30 to 1.00)	0
Low SDI	4396 (3518–5315)	11.78 (9.43–14.25)	10561 (8860–12437)	12.87 (10.80–15.16)	0.28 (0.26 to 0.31)	0	83494 (42755–118624)	223.80 (114.60–317.96)	200174 (122961–283961)	243.94 (149.85–346.05)	0.34 (–0.02 to 0.70)	0.066
Middle SDI	15550 (12507–18762)	8.96 (7.21–10.81)	60843 (51640–70725)	12.95 (10.99–15.05)	1.21(1.14 to 1.28)	0	164934 (130961–223594)	95.03 (75.46–128.83)	610413 (519978–736412)	129.91 (110.67–156.73)	1.04 (0.81 to 1.28)	0
Region												
Andean Latin America	1848 (1666–2065)	55.07 (49.65–61.53)	10032 (9315–10747)	101.26 (94.03–108.48)	1.99 (1.95 to 2.03)	0	22952 (16755–32220)	683.94 (499.27–960.10)	100282 (78652–124465)	1012.29 (793.95–1256.40)	1.32 (0.92 to 1.72)	0
Australasia	612 (538–699)	15.54 (13.65–17.74)	3027 (2690–3348)	34.27 (30.46–37.90)	2.58 (2.51 to 2.64)	0	4982 (4561–5386)	126.46 (115.78–136.72)	30157 (26127–32901)	341.36 (295.75–372.42)	3.16 (2.49 to 3.84)	0
Caribbean	204 (173–234)	4.72 (4.03–5.44)	630 (554–707)	6.81 (5.98–7.63)	1.19 (1.15 to 1.22)	0	2854 (2333–3480)	66.21 (54.14–80.75)	10288 (8724–12346)	111.12 (94.23–133.35)	1.72 (1.05 to 2.40)	0
Central Asia	917 (778–1053)	11.47 (9.73–13.17)	1666 (1464–1892)	11.45 (10.06–13.00)	–0.01 (–0.08 to 0.06)	0.808	11604 (10026–13398)	145.09 (125.36–167.52)	11790 (9701–14344)	81.03 (66.68–98.59)	–1.87 (–2.75 to –0.98)	0
Central Europe	1779 (1442–2144)	6.71 (5.44–8.08)	2351 (1993–2778)	6.35 (5.38–7.50)	–0.16 (–0.21 to –0.12)	0	29128 (27053–31542)	109.83 (102.01–118.93)	41514 (37832–45354)	112.11 (102.17–122.49)	0.03 (–0.38 to 0.44)	0.893
Central Latin America	1784 (1465–2110)	13.14 (10.79–15.55)	8515 (7390–9725)	19.91 (17.28–22.74)	1.35 (1.33 to 1.37)	0	18841 (17703–20182)	138.84 (130.45–148.73)	110450 (100242–121597)	258.26 (234.39–284.33)	2.01 (1.91 to 2.10)	0
Central Sub–Saharan Africa	218 (171–265)	5.80 (4.54–7.05)	550 (449–656)	6.09 (4.98–7.27)	0.16 (0.14 to 0.18)	0	4764 (1803–10057)	126.69 (47.95–267.46)	11773 (4616–28258)	130.47 (51.15–313.16)	0.10 (0.04 to 0.16)	0.001
East Asia	8341 (6252–10630)	5.60 (4.20–7.14)	34379 (27963–40825)	8.77 (7.13–10.41)	1.44 (1.25 to 1.63)	0	66164 (50645–99358)	44.42 (34.00–66.70)	189123 (134579–241957)	48.23 (34.32–61.70)	0.26 (0.14 to 0.38)	0
Eastern Europe	2060 (1524–2698)	4.21 (3.12–5.52)	1137 (871–1436)	1.83 (1.40–2.31)	–2.67 (–2.75 to –2.59)	0	53681 (49474–57952)	109.79 (101.19–118.53)	23527 (21470–25924)	37.90 (34.59–41.76)	–3.20 (–4.27 to –2.12)	0
Eastern Sub–Saharan Africa	624 (499–748)	5.13 (4.10–6.15)	1408 (1152–1661)	5.21 (4.26–6.14)	0.05 (0.03 to 0.07)	0	11711 (4449–20449)	96.26 (36.57–168.08)	24674 (9695–50121)	91.26 (35.86–185.38)	–0.17 (–0.22 to –0.11)	0
High–income Asia Pacific	12199 (9667–15031)	34.89 (27.65–42.98)	36996 (31811–42749)	52.47 (45.12–60.63)	1.31 (1.26 to 1.37)	0	110175 (100366–120262)	315.07 (287.02–343.92)	412038 (361512–453592)	584.42 (512.76–643.36)	2.01 (1.66 to 2.37)	0
High–income North America	18123 (14703–21583)	31.29 (25.38–37.26)	54502 (46352–63559)	48.43 (41.19–56.48)	1.47 (1.34 to 1.58)	0	155417 (143662–167533)	268.29 (248.00–289.21)	515124 (466454–549497)	457.75 (414.50–488.29)	1.75 (1.38 to 2.11)	0
North Africa and Middle East	1911 (1544–2296)	6.76 (5.46–8.12)	6913 (5814–8190)	9.07 (7.63–10.74)	0.96 (0.93 to 0.98)	0	17076 (12420–24808)	60.42 (43.94–87.77)	57770 (44428–83023)	75.78 (58.28–108.91)	0.71 (0.63 to 0.80)	0
Oceania	52 (43–62)	10.72 (8.87–12.96)	153 (130–177)	12.43 (10.50–14.37)	0.47 (0.45 to 0.50)	0	849 (569–1410)	176.52 (118.22–293.05)	2296 (1370–3962)	186.07 (111.01–321.02)	0.20 (0.11 to 0.28)	0
South Asia	22037 (17558–26683)	23.21 (18.49–28.10)	65075 (54107–77326)	26.21 (21.79–31.14)	0.39 (0.37 to 0.42)	0	336098 (193733–513482)	354.00 (204.05–540.84)	1017920 (679699–1371890)	409.96 (273.75–552.52)	0.51 (0.09 to 0.92)	0.016
Southeast Asia	2071 (1648–2498)	4.89 (3.89–5.90)	6816 (5639–8045)	5.95 (4.92–7.02)	0.63 (0.61 to 0.65)	0	12751 (7033–25409)	30.11 (16.61–60.01)	38433 (22547–69745)	33.55 (19.68–60.88)	0.36 (0.26 to 0.46)	0
Southern Latin America	1914 (1712–2149)	24.16 (21.61–27.13)	6735 (6198–7360)	45.77 (42.12–50.01)	2.09 (2.05 to 2.13)	0	21123 (19552–22754)	266.66 (246.83–287.25)	74183 (67944–79476)	504.10 (461.70–540.06)	2.17 (1.53 to 2.81)	0
Southern Sub–Saharan Africa	764 (620–915)	17.27 (14.01–20.68)	1522 (1241–1814)	15.63 (12.74–18.63)	–0.33 (–0.37 to –0.29)	0	8169 (4464–12209)	184.61 (100.89–275.92)	17924 (12610–23156)	184.11 (129.53–237.85)	–0.06 (–0.46 to 0.35)	0.782
Tropical Latin America	1110 (875–1348)	7.33 (5.78–8.90)	5044 (4263–5800)	11.39 (9.62–13.09)	1.42 (1.37 to 1.48)	0	13658 (12761–14633)	90.20 (84.28–96.64)	74555 (68107–79357)	168.30 (153.75–179.14)	2.07 (1.67 to 2.47)	0
Western Europe	13067 (11278–15118)	13.46 (11.61–15.57)	36335 (32365–40459)	24.36 (21.70–27.13)	1.94 (1.87 to 2.00)	0	130685 (121703–138962)	134.57 (125.32–143.09)	479906 (434189–511020)	321.79 (291.14–342.66)	2.88 (2.44 to 3.33)	0
Western Sub–Saharan Africa	670 (536–816)	4.64 (3.71–5.65)	1100 (903–1309)	3.42 (2.81–4.07)	–0.98 (–1.00 to –0.96)	0	23639 (9282–36943)	163.76 (64.30–255.92)	40527 (16536–69404)	126.08 (51.44–215.92)	–0.85 (–0.91 to –0.78)	0

Data in parentheses are 95% uncertainty intervals for cases, deaths, and prevalence, and 95% CIs for AAPCs. UI=uncertainty interval. CI=confidence interval. AAPC=average annual percentage change

**Fig 1:**
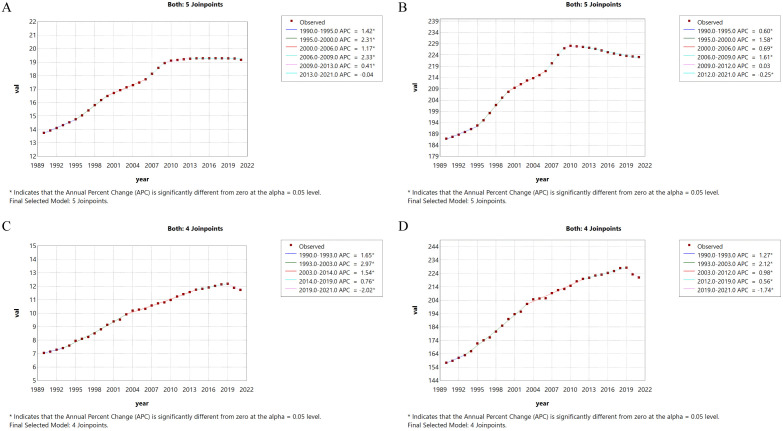
Joinpoint Regression analysis for global incidence of ILD&PS (A), prevalence (B), deaths (C) and DALYs (D) in adults aged 55 + years from 1990 to 2021.

As shown by our analysis, the global burden of ILD&PS in the population aged 55 years and older underwent notable changes in incidence, death, and DALYs rates over the past 31 years, while the prevalence rate increased only modestly. Therefore, it is important to examine the underlying patterns behind these trends. Using APC and AAPC methods, we performed a detailed analysis that revealed differing trajectories: the global prevalence rate showed an overall sustained increase, with the most rapid growth occurring from 2006 to 2009 (APC = 2.33, 95% CI: 1.94 to 2.73), followed by a slight downward trend between 2013 and 2021 (APC = –0.04). Joinpoint regression identified six change points for incidence and prevalence, five of which—1990, 1995, 2000, 2006, and 2009—coincided and exhibited similar upward tendencies (Fig 1). Both death and DALYs rates rose rapidly initially, then slowed, and eventually entered a phase of marked decline (death rate APC = –2.02; DALYs rate APC = –1.74). Moreover, integrated point analysis in Fig 1 confirmed that death and DALYs rates varied substantially across the same five time points—1990, 1993, 2003, and 2019—with 2019 representing a transition point. Detailed results are provided in Table 1.

### Global trends by Age and sex patterns

**Fig 2:**
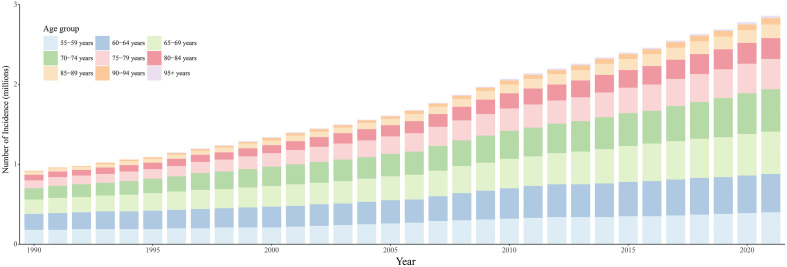
The trends in incidence cases of ILD&PS in specific age group ranging from 55 years to 95 + years over 1990-2021.

All age groups showed an upward trend in the number of incident cases, with the smallest increase observed in the ≥ 95‑year age group from 1990 to 2021 (Fig 2). In 2021, the incidence, mortality, and DALYs rates generally increased with age, without reaching a clear plateau, suggesting that the burden of ILD&PS may continue to rise with advancing age (S2 Fig). In contrast, the prevalence rate did not consistently increase with age. No significant sex‑based differences were observed in prevalence (S3 Fig). In the over‑85 age group, the mortality rate exceeded 20 per 100,000, while in the 55–64 age group it remained below 4 per 100,000. Notably, the mortality rate peaked sharply in the ≥ 95‑year age group at 101.57 per 100,000 (95% UI: 73.17–118.39), representing a 2.5‑fold increase from 1990.

Tables 1 and S1 Table indicate that the largest increase in incidence rate occurred in the 75–79 year group, with an AAPC of 1.74 (95% CI: 1.65 to 1.82), while the smallest increase was seen in the 55–59 year group (AAPC: 0.17; 95% CI: 0.09 to 0.24). The ≥ 95‑year group recorded the most pronounced increases in both DALYs and deaths rates. It is noteworthy that the prevalence rate in the 55–59 year group showed a declining trend unique among all age groups over the same period, decreasing moderately from 115.48 per 100,000 in 1990 to 108.96 per 100,000 in 2021. Nevertheless, the absolute number of prevalent cases continued to rise.

**Fig 3:**
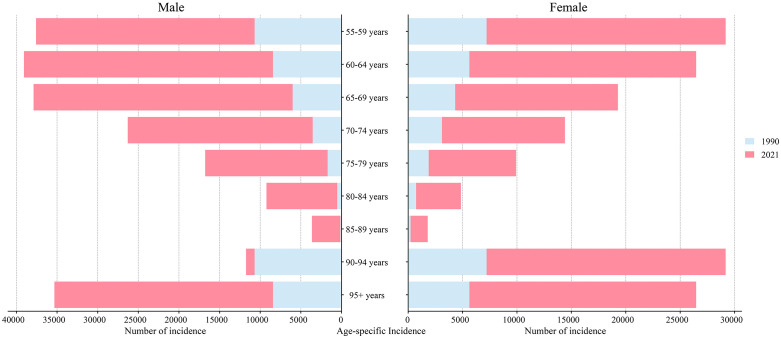
Trends in the incidence of ILD&PS by sex and across age groups (55–95 + years) in 1990 and 2021.

Furthermore, Fig 3 and S4 Fig illustrate that the disease burden in males consistently exceeded that in females, and the sex‑based disparity widened with increasing age. In 1990, the number of cases initially declined with age, reaching a nadir in the 85–89 year group before rising again in both sexes. A similar pattern was evident in 2021. However, the lowest number of incident cases was observed among males in the 90–94 year group compared with all other age groups in the same year. As of 2021, the global incidence rate was approximately 1.5 times higher in males than in females, with values of 15.59 per 100,000 (13.30–18.09) in females and 23.20 per 100,000 (20.04–26.71) in males. Over the past 31 years, however, the increase in incidence was particularly marked in females, nearly catching up to the growth seen in males. Regarding prevalence, both sexes exhibited mild and steady increases, though the rise was less pronounced in females (AAPC = 0.50; 95% CI: 0.46–0.54) than in males (AAPC = 0.64; 95% CI: 0.59–0.69).

### Global trends by SDI

**Fig 4:**
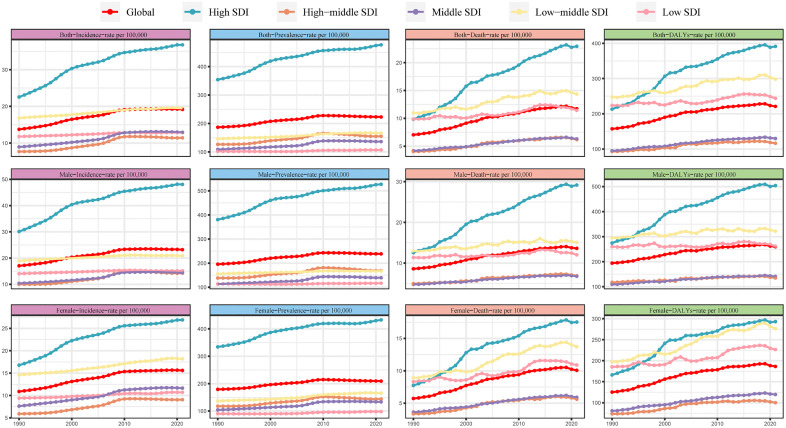
Changes in total rates in incidence, prevalence, death, and DALYs for ILD&PS in both sexes combined, females and males individually, and in global and 5 SDI quintiles regions (1990–2021). All countries and territories were categorized into five groups based on sociodemographic index quintiles: Low SDI, Low-middle SDI, middle SDI, High-middle SDI, and High SDI.

Using 1990 as the baseline, varying degrees of upward trends were observed across all age groups within each SDI region by 2021, revealing distinct trajectories in disease burden. Fig 4 illustrates the temporal evolution of the burden of ILD&PS by sex, highlighting substantial heterogeneity over the past 30 years. Values for all four metrics showed consistent increases. The High SDI region exhibited the most pronounced rise, with incidence rates exceeding global averages across all sex subgroups, whereas the High‑middle SDI region recorded the lowest prevalence at 11.44 per 100,000 (95% UI: 9.90–13.13). Regarding prevalence, the overall upward trend across SDI regions was not particularly pronounced and generally aligned with global patterns. In terms of mortality, an increasing trend was mainly evident in the High, High‑middle, and Middle SDI regions, with the greatest increase occurring in the High SDI region (AAPC: 2.78; 95% CI: 2.45 to 3.11). Even in Low‑middle SDI regions, where only a modest change in DALYs rates was observed, the number in 2021 rose to 71.8 per 100,000 (95% UI: 14.84–37.64) from 24.9 (95% UI: 14.84–37.64) in 1990. However, DALYs rates in High and Middle SDI regions showed a marked upward trend. In contrast, the High SDI region experienced the fastest increase in DALYs rates, with an AAPC of 2.95 (95% CI: 1.75 to 2.25). Overall, the incidence rate in High SDI regions continued to surpass the global average, while the High‑middle SDI region recorded the lowest rates.

### Regional trends

By 2021, Andean Latin America recorded the highest incidence rate, at 101.26 per 100,000 (95% UI: 94.03–108.48), along with the highest DALYs (1012.29 per 100,000; 95% UI: 793.95–1256.40) and mortality rates, substantially exceeding those of the other 20 regions. Meanwhile, High‑income Asia Pacific ranked first in prevalence, at 807.82 per 100,000 (95% UI: 703.57–925.40), in contrast to Western Sub‑Saharan Africa (44.83 per 100,000; 95% UI: 38.33–52.51). With respect to mortality, Southeast Asia had the lowest rate at 1.34 per 100,000 (95% UI: 0.69–2.66), consistent with its 1990 level. Table 1 summarizes the regional variations in disease incidence.

From 1990 to 2021, the prevalence rate in the population aged ≥55 years exhibited distinct geographic patterns. Andean Latin America showed a marked upward trend worldwide, with an AAPC of 2.16 (95% CI: 2.11 to 2.20). Notably, Australasia experienced the fastest increases in incidence, DALYs, and mortality. In stark contrast, Eastern Europe consistently had the lowest rates for all measures.

**Fig 5:**
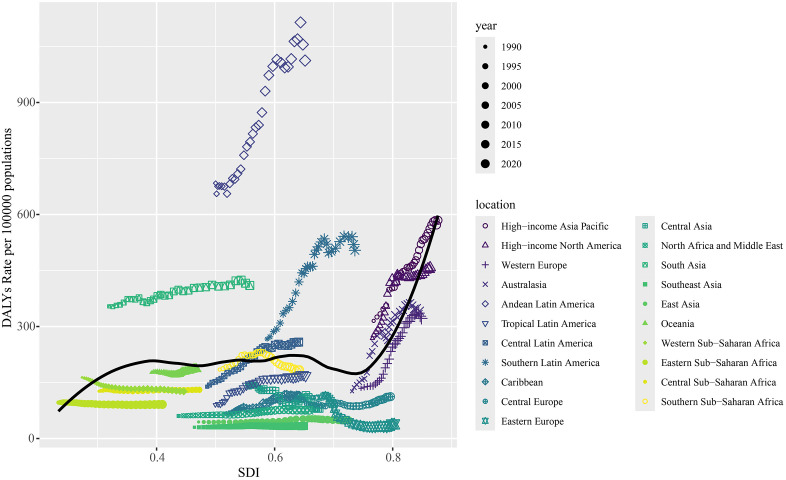
The DALYs rates stratified by SDI for ILD&PS globally and for 21 disease burden regions from 1990 to 2021. The DALYs rates (based solely on SDI) are represented by the black line. For each region, the dots from left to right indicate annual estimates.

**Fig 6:**
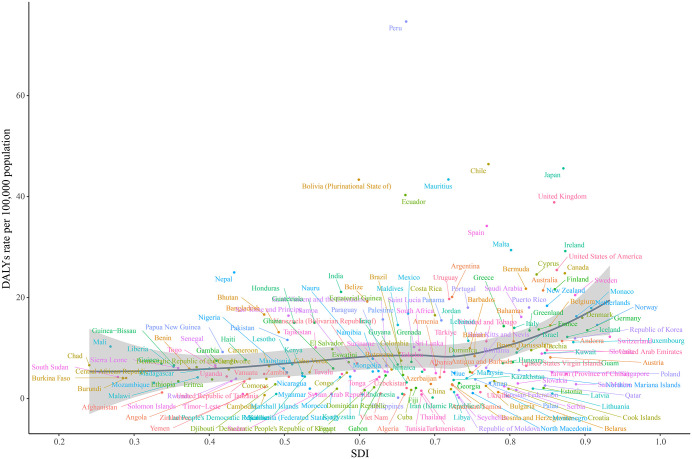
SDI correlation analysis of DALYs rates for 204 countries in 2021. DALY rates for 2021 (based on SDI only) are indicated by the black line.

Fig 5 and S5 Fig illustrate the correlation between the expected SDI and the rates of incidence, prevalence, mortality, and DALYs across the 21 GBD regions. Spearman correlation analysis indicated that all burden estimates increased with rising SDI. The prevalence rate showed the strongest positive correlation with SDI (*R* = 0.584, *P* < 0.001), followed by the incidence rate (*R* = 0.430, *P* < 0.001). Andean Latin America and Southern Latin America underwent substantial increases during 1990–2021, with South Asia also showing SDI values consistently above expected levels. However, SDI levels deviated from expectations in some areas, while others remained well ahead of the projected trend. When examined at specific time points, each SDI region displayed distinct patterns between 2009 and 2021. For instance, Southeast Asia, North Africa and the Middle East, and East Asia maintained relatively stable trends. The relationship between DALYs rate and SDI in Andean Latin America exhibited a U‑shaped pattern, rising initially and then declining. Southern Latin America followed an inverted U‑shaped (M‑shaped) curve. A broader analysis across 204 countries and territories confirmed these patterns (Fig 6 and S6 Fig), which will be discussed further in the following section.

### National trends

At the national level, Peru exhibited the most pronounced increases in incidence, mortality, and DALYs rates among the 204 countries and territories (S2–S3 Tables). The highest prevalence rate was observed in Japan (907.98 per 100,000; 95% UI: 778.56–1051.57), while the Philippines reported the lowest prevalence, at 29.76 per 100,000 (95% UI: 24.29–36.15). Additionally, the Philippines also had the lowest mortality rate, at 0.19 per 100,000 (95% UI: 0.11–0.27), in sharp contrast to Peru, which reported the highest mortality rate of 74.67 per 100,000 (95% UI: 51.74–98.52).

**Fig 7:**
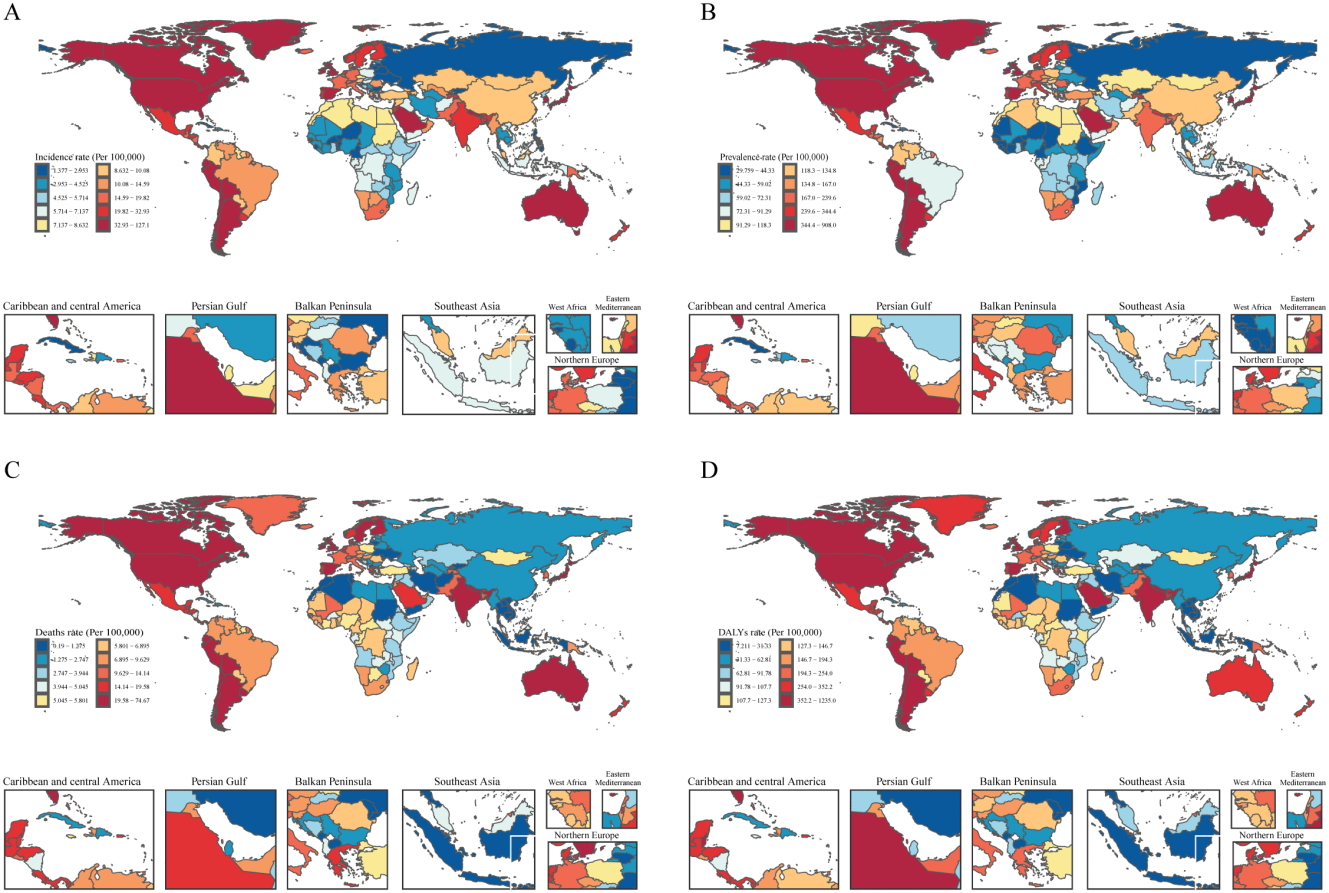
Global map of incidence (A), prevalence (B), deaths (C) and Prevalence DALYs (D) rates (per 100,000 population) in 2021 in 204 countries and territories. Note: Base map from Natural Earth (public domain). Map created by the authors in R.

When comparing the period 1990–2021, the Netherlands showed the greatest progressive increase in incidence rate, with an AAPC of 3.09 (95% CI: 3.00 to 3.18), whereas Belarus experienced the largest decline. Meanwhile, Taiwan (Province of China) recorded the most marked rise in prevalence rate, with an AAPC of 2.72 (95% CI: 2.64 to 2.80). Conversely, Ukraine showed the greatest decrease (AAPC: –4.26; 95% CI: –4.37 to –4.15). Italy led globally in the growth of both DALYs and mortality rates. The greatest reduction in mortality rate was seen in the Republic of Moldova, with an AAPC of –7.52 (95% CI: –8.49 to –6.53). Detailed data are presented in Fig 7.

### Global disease burden prediction for ILD&PS to 2035

**Fig 8:**
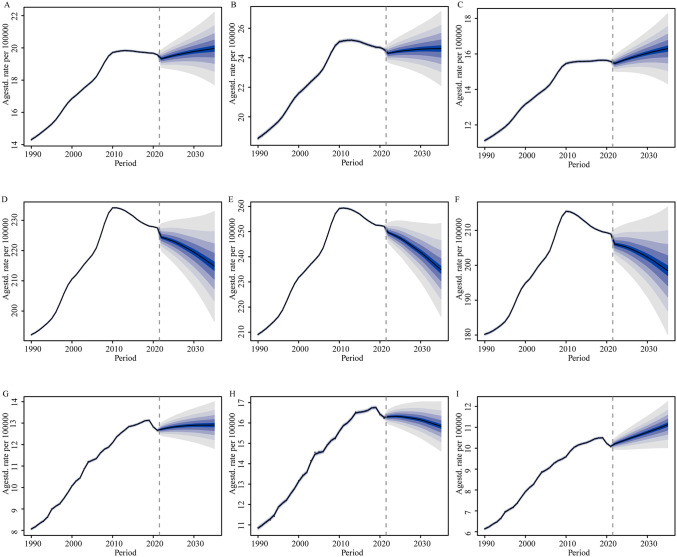
Trends in rates from 2019 to 2035, predicted using BAPC prediction models. The rows, from top to bottom, show incidence, prevalence, and mortality rates, and the three columns correspond to males and females. Solid points represent observed data, while shaded bands illustrate the predicted distribution between the 2.5% and 97.5% quantiles. The projected mean is shown as a solid line, and the vertical dashed line indicates the start of the prediction period.

The predicted age-standardized prevalence rates closely matched observed values during the validation period (2017–2021), with most estimates falling within the 95% credible intervals, demonstrating the strong predictive performance of the BAPC model. The predicted values were compared with the actual observations during the validating-period, and the results are summarized as root mean square error (RMSE) (S7 Fig and S4–S5 Tables). The BAPC model was used to project future trends in incidence, prevalence, deaths, and corresponding case counts (Fig 8) (S6–S7 Tables). The forecast results indicate that for the population aged 55 years and older, incidence is projected to show an overall upward trend from 2022 to 2035, while prevalence and mortality are expected to follow a pattern of initial increase followed by a decline. By 2035, the global prevalence rate is estimated to reach 214 per 100,000 (95% UI: 193–236). Projected incidence trends were examined separately by age group. For the 55–69 year age groups, incidence is expected to exhibit relatively stable, modest growth. In the 70–74 and 75–79 year age groups, incidence is projected to rise initially and then stabilize. Additionally, the model predicts that incidence in the 85–94 and ≥95 year age groups will follow a W‑shaped pattern: an initial increase, followed by a decline, and then another rise. Regarding sex differences, both males and females are projected to experience a mild decline in incidence after a period of sustained increase, a pattern expected to continue through 2035. However, the rise in incidence is projected to be more pronounced in females. At the same time, mortality is expected to continue declining in males, whereas an increase is anticipated in females. As for prevalence, a gradual downward trend is projected for both sexes. To better illustrate the projected burden trends of ILD&PS globally, Fig 8 and the Supplementary Material present results stratified by age group, sex, and all measured indicators.

## Discussion

### Global disease burden among individuals over 55 continues to worsen

This study utilized the GBD 2021 database to comprehensively and systematically examine the global burden and trends of ILD&PS in individuals aged 55 years and older from 1990 to 2021, and to project future trends through 2035. Our findings indicate that over the past three decades, global incidence, prevalence, mortality, and DALYs rates in this age group have all risen, with incidence increasing by nearly 1.4 times and mortality by approximately 1.7 times. According to joinpoint regression analysis, the most pronounced increases in overall incidence (APC = 2.33) and prevalence (APC = 1.61) of ILD&PS occurred between 2006 and 2009. These trends may be attributed to the heterogeneity and complexity of ILD, as well as the nonspecific nature of its early clinical manifestations, which often require confirmation through high-resolution computed tomography (HRCT), bronchoalveolar lavage (BAL), or even lung biopsy and pathological examination [28]. Furthermore, until the early 21st century, diagnosis was not standardized and relied heavily on clinical experience, introducing considerable uncertainty. In recent years, multidisciplinary discussions have provided valuable insights for diagnosing ILD, and their importance is increasingly recognized [12,29]. Advances in high‑technology fields such as next‑generation sequencing (NGS), mass spectrometry (MS), and computer science (CS) have further enhanced diagnostic reliability. On the other hand, according to the United Nations Department of Economic and Social Affairs, Population Division’s World Population Prospects 2022 report [30,31], the global population continues to grow, albeit at a slowing rate. This demographic trend is closely associated with declining mortality and rising life expectancy at birth. At the same time, the elderly population—both in absolute numbers and as a proportion of the total population—has expanded substantially, while the incidence of ILD is strongly linked to aging. Consequently, there is an urgent need to adapt public health programs to the growing proportion of older adults. Ensuring universal health coverage and establishing sustainable long‑term care systems are critical priorities. Encouragingly, all four indicators have shown a gradual decline in recent years, particularly between 2019 and 2021. This improvement is likely due to enhanced quality of life and survival rates resulting from numerous clinical trials and novel therapies, including antifibrotic agents, immunosuppressive medications, and advances in lung transplantation technology. Although the World Health Organization (WHO) estimated that global mortality attributable to the COVID‑19 pandemic may have exceeded 14.9 million by the end of 2021 [32], previous studies have reported a positive correlation between ILD‑related mortality and COVID‑19 mortality [33,34]. This observed association contrasts with the findings of the present study, and the underlying reasons remain a subject for further investigation.

### Burden of disease is tightly correlated with gender and positively correlated with age

Our findings indicate that among individuals aged over 55 years, global rates of incidence, mortality, DALYs, and prevalence all exceed 70%. The link between age and ILD may arise from the biological process of telomere shortening [35]. Telomere length determines the frequency of cell division, which in turn regulates cellular senescence and apoptosis. Consistent with prior studies, endoplasmic reticulum stress has been observed to contribute to epithelial cell dysfunction and the fibrotic response, suggesting a potential mechanism connecting aging and IPF [36]. Therefore, our research focuses on the population aged over 55 years. Furthermore, we conducted a systematic analysis of the study cohort, dividing it into subgroups at 5‑year age intervals. All targeted measures—except prevalence—show a rapid upward trend, underscoring that aging is a significant risk factor for disease progression. The rising burden can also be attributed to age‑related declines in lung function and increased susceptibility [37]. Incidence rates exhibited a rapid rise followed by a gradual decline, peaking in the 85–89‑year age group. This highlights the need for early diagnosis and treatment of ILD, as well as the importance of pulmonary rehabilitation in alleviating symptoms and improving patient outcomes [38]. Cross‑sectional comparisons revealed that all four indicators increased multiplicatively across subgroups. The growth in overall incidence and mortality appears to be faster in females than in males, consistent with the findings of Olson et al. [39]. This may be related to the higher incidence of connective tissue diseases in women. Although the overall disease burden is lower in females than in males, this is likely attributable to their generally more limited smoking history [10]. Tobacco exposure damages epithelial cells, leading to increased production of TGF‑β1, which promotes secretion and extracellular matrix deposition [8]. Moreover, existing research confirms a clear association between tobacco use and respiratory bronchiolitis‑associated interstitial lung disease, desquamative interstitial pneumonia, Langerhans cell histiocytosis, and acute eosinophilic pneumonia [40].

### High SDI regions experience high overall disease burden, with variation in regional country distribution

Rates of incidence, prevalence, mortality, and DALYs were significantly elevated in High SDI regions, followed by Low‑middle SDI regions. Geographically, the burden is predominantly concentrated in Andean Latin America and High‑income Asia Pacific, where these metrics are substantially higher than in other regions [41]. Unhealthy lifestyle factors prevalent in High SDI regions—such as smoking, high body mass index (BMI), and hyperglycemia—are likely among the leading risk factors for ILD. Additionally, High SDI regions typically possess more advanced medical technologies and greater resources for diagnosing ILD compared to Middle or Low SDI regions, which may contribute to higher reported case numbers. In contrast, Low‑middle SDI regions are often characterized by greater exposure to environmental risk factors, including air pollution and occupational hazards, potentially leading to comparable adverse health outcomes [42]. Socioeconomically, the high cost and long‑term requirement of antifibrotic medications make sustained treatment challenging for many patients. Thus, in Low‑middle SDI regions, an under‑resourced healthcare system conflicts with the high costs of care, likely explaining part of the observed increase in disease burden. Some studies also suggest that lower income is closely associated with a higher incidence of sarcoidosis [43].

Globally, inadequate disease management and social support systems persist, which can accelerate disease progression. Addressing this issue requires implementing multifaceted strategies, including developing and refining more comprehensive public health policies and medical insurance systems. Notably, Oceania—where most cases occur at older ages—showed the greatest increase in incidence and mortality rates compared to other regions, consistent with findings from Cox’s team [44,45]. Similarly, data from the Australasian Interstitial Lung Disease Registry indicate that the average age at disease onset is 68.3 ± 12.5 years, highlighting the need to enhance overall disease management and deepen healthcare coverage [46].

As notable examples of high‑risk countries for ILD&PS in the population over 55, Peru recorded the highest incidence and mortality rates, followed by Chile. Since 2021, Peru#39;s incidence rate reached 127.09 per 100,000, markedly exceeding rates in other countries and regions. Geographically, Peru and Chile—neighboring countries in western South America—are rich in mineral resources. Mining‑related dust exposure is a recognized major risk factor for the disease [42]，making residents near such sites a continued public health concern. It is recommended that local governments implement rational planning, enforce rigorous environmental quality monitoring, and collaborate with industries to reduce pollution. Despite geographical variations in ILD distribution, epidemiological research remains sparse in several regions, including parts of Latin America. The relationship between population genetic predisposition and the disease across all regions has yet to be fully elucidated [45].

Among all countries, Belarus and Ukraine—both in Eastern Europe—ranked first and second for the largest declines in incidence rates, with AAPCs of –4.13 and –3.92, respectively. This decline may be linked to multiple factors, such as healthcare system improvements, experience of major socioeconomic transitions, slowed industrialization, and strengthened environmental protections, all contributing to better patient quality of life. In East Asia, India and China have large populations and notably higher disease incidence rates than many other countries. A multicenter cohort study in India reported a mean patient age of 55.3 years (47.2% male), with hypersensitivity pneumonitis (HP) as the most common ILD subtype; only 7.5% of diagnoses were confirmed by lung biopsy [47]. A retrospective study of 1,945 subjects in China found a mean age of 57.9 years (55.5% male), with IPF being the most prevalent subtype (20.3%) [48]. These findings underscore that healthcare and health protection systems in both countries require strengthening. The above evidence confirms that robust medical health assurance is a prerequisite for clinicians to provide timely diagnosis and treatment for ILD&PS, which helps improve patients’ quality of life and prolong survival.

### The overall burden trend of disease is shifting to smooth for the age of 55+ in the next 14 years

From a research perspective, BAPC model forecasts future trends in disease incidence and mortality, thereby aiding public health policymakers in planning resources and interventions. Projections suggest that from 2022 to 2035, overall incidence and mortality rates will likely show a steady increase. Specifically, the incidence rate is predicted to rise from 19.32 to 19.96 per 100,000, and the death rate is anticipated to increase from 13.06 to 14.20 per 100,000 by 2035. Notably, among adults over 55 years of age, the trend no longer follows a linear rise; instead, incidence peaks in the 70–74, 75–79, 80–84, and 85–89 age groups before beginning to decline. This pattern can be attributed to several factors. Advances in economic development and medical technology have facilitated the use of molecular phenotypes, genetic variants, and markers of telomere dysfunction as biomarkers to guide treatment and prognosis. In addition, the ongoing development and increasing availability of novel therapies that target aberrant immune responses or pro‑fibrotic pathways have collectively contributed to reducing both incidence and mortality [49]. On the other hand, heightened individual awareness of healthy lifestyles and improvements in quality of life—such as reduced environmental exposures and declining smoking rates—also play a significant role.

### Strengths and limitations

To the best of our knowledge, this study is the first to comprehensively assess the global burden of ILD&PS across four primary measurable indicators—incidence, prevalence, mortality, and DALYs—in adults aged 55 years and older, along with its spatiotemporal distribution patterns. Furthermore, we have projected the overall disease burden through 2035.

Our study is, however, subject to several limitations. First, GBD data are derived from a wide range of sources, including official statistics, surveys, and published studies. Nevertheless, differences in diagnostic criteria and disease classification systems across countries and over time may exist. Additionally, regional variations in data collection methods may affect the quality and completeness of the data. Second, although the GBD database offers broad coverage, the absence of data on identifiable risk factors specific to ILD&PS makes it difficult for policymakers to develop targeted primary and secondary prevention strategies based on modifiable risks. Third, the GBD study does not provide burden estimates stratified by ethnicity. Health behaviors and medical practices rooted in different cultural and social contexts may also influence disease burden, yet these factors are not fully accounted for in the GBD database. Fourth, the burden of ILD&PS can be highly uneven within a given country. For example, large or economically developed nations may exhibit substantial subnational disparities due to varying provincial priorities or differences in developmental trajectories. Therefore, assessing burden at subnational levels is crucial. Fifth, recent public health initiatives aimed at specific age groups may have influenced observed temporal trends in disease burden. Future research should therefore include more comprehensive comparisons of model assumptions and methodologies—through sensitivity analyses or cross-validation—to ensure that trend forecasts are both reliable and robust.

## Conclusion

In summary, the global burden of ILD&PS in adults aged 55 years and older continues to rise amid population growth and aging, representing a growing public health challenge. The burden varies substantially across regions and countries. Therefore, it is crucial for policymakers to strengthen comprehensive strategies across the continuum of prevention, diagnosis, treatment, and rehabilitation in order to effectively address current and future healthcare needs.List of abbreviations.

## Supporting information

S1 Table Global Deaths and Prevalence of ILD&PS and their AAPCs globally by gender, specific age group, SDI, and region from 1990 to 2021. Abbreviations: ILD&PS, Interstitial lung disease and pulmonary sarcoidosis; AAPC, average annual per centage change; SDI, Sociodemographic Index; UI, uncertainty interval.
(PDF)

S2 Table Global Incidence and DALYs of ILD&PS and their AAPCs globally by country from 1990 to 2021. Abbreviations: ILD&PS, Interstitial lung disease and pulmonary sarcoidosis; AAPC, average annual per centage change; DALYs, disability-adjusted life-years; UI, uncertainty interval.
(PDF)

S3 Table Global Prevalence and Deaths of ILD&PS and their AAPCs globally by country from 1990 to 2021. Abbreviations: ILD&PS, Interstitial lung disease and pulmonary sarcoidosis; AAPC, average annual per centage change; UI, uncertainty interval.
(PDF)

S4 Table Predicted and Observed Values from BAPC Retrospective Validation (1990–2021). (PDF)

S5 Table Model performance evaluation. (PDF)

S6 Table BAPC-modeled projections: incidence, prevalence, and deaths by 5‑year age group in 2035. Abbreviations: BAPC, Bayesian age–period–cohort.
(PDF)

S7 Table Projected incidence, prevalence, and mortality by 5‑year age group in 2035 using the BAPC model. Abbreviations: BAPC, Bayesian age–period–cohort.
(PDF)

S1 Fig Burden comparison of ILD&PS: number of cases, deaths, and DALYs in populations below and above 55 years of age. Abbreviations: ILD&PS, Interstitial lung disease and pulmonary sarcoidosis; DALYs, disability-adjusted life-years.
(PDF)

S2 Fig Numbers and age‑standardized rates of incidence, prevalence, deaths, and DALYs for ILD&PS among individuals aged ≥55 years, by sex, in 2021. Abbreviations: ILD&PS, Interstitial lung disease and pulmonary sarcoidosis; DALYs, disability-adjusted life-years.
(PDF)

S3 Fig The trends in prevalence, deaths and DALYs cases of ILD&PS in specific age group ranging from 55 years to 95 + years over 1990–2021. Abbreviations: ILD&PS, Interstitial lung disease and pulmonary sarcoidosis; DALYs, disability-adjusted life-years.
(PDF)

S4 Fig Trends in prevalence, deaths, and DALYs for ILD&PS by sex and age group (55–95 + years) in 1990 and 2021. Abbreviations: ILD&PS, Interstitial lung disease and pulmonary sarcoidosis; DALYs, disability-adjusted life-years.
(PDF)

S5 Fig The temporal trends and correlation of incidence, prevalence and deaths with SDI from 1990 to 2021 in global and 21 regions. Abbreviations: SDI, Sociodemographic Index.
(PDF)

S6 Fig SDI correlation analysis of incidence, prevalence, and death rates for 204 countries in 2021. Abbreviations: SDI, Sociodemographic Index.
(PDF)

S7 Fig A back-testing procedure by fitting the model on data from 1990–2016 and forecasting the incidence(A), deaths(B), and prevalence(C) for 2017–2021. (PDF)
