# Supplementary material for: Global burden, trends and projections analysis of interstitial lung disease and pulmonary sarcoidosis in elderly adults (aged 55+ Years) based on GBD 2021
Source: PLoS One. 2026 Apr 20;21(4):e0347482. doi: 10.1371/journal.pone.0347482 (PMC13095001; doi:10.1371/journal.pone.0347482)
Supplement: S1 Table — Abbreviations: ILD&PS, Interstitial lung disease and pulmonary sarcoidosis; AAPC, average annual per centage change; SDI, Sociodemographic Index; UI, uncertainty interval. (PDF) [file pone.0347482.s001.pdf]

Supplementary table 1 Global Deaths and Prevalence of ILD&PS and their AAPCs globally by gender, specific age group, SDI, and region from 1990 to 2021.

	Deaths						Prevalence					
	1990	1990	2021	2021	1990–2021	*P* value	1990	1990	2021	2021	1990–2021	*P* value
	Cases (n)	Rate (per 100 000 population)	Cases (n)	Rate (per 100 000 population)	AAPC (95%)		Cases (n)	Rate (per 100 000 population)	Cases (n)	Rate (per 100 000 population)	AAPC (95%)	
Global	47353 (39013-57966)	7.05 (5.81-8.63)	174368 (149929-196275)	11.73 (10.09-13.21)	1.65 (1.47 to 1.83)	0	1254751 (1067431-1473565)	186.88 (158.98-219.47)	3314679 (2897474-3787334)	223.06 (194.99-254.87)	0.57 (0.53 to 0.61)	0
Sex												
Female	20600 (15934-28280)	5.72 (4.43-7.86)	79204 (64061-97610)	10.07 (8.15-12.41)	1.84 (1.62 to 2.05)	0	644944 (554980-754722)	179.18 (154.19-209.68)	1646113 (1437555-1875154)	209.30 (182.79-238.43)	0.50 (0.46 to 0.54)	0
Male	26753 (20936-33114)	8.59 (6.72-10.63)	95163 (78274-106571)	13.60 (11.19-15.23)	1.49 (1.31 to 1.67)	0	609807 (515790-718473)	195.78 (165.59-230.66)	1668566 (1452114-1905870)	238.53 (207.59-272.45)	0.64 (0.59 to 0.69)	0
Age group												
55-59 years	3869 (2913-5129)	2.09 (1.57-2.77)	8544 (6737-10400)	2.16 (1.70-2.63)	0.12 (-0.10 to 0.35)	0.278	213865 (164227-265975)	115.48 (88.68-143.61)	431200 (342749-535796)	108.96 (86.61-135.40)	-0.20 (-0.25 to -0.14)	0
60-64 years	5884 (4419-7866)	3.66 (2.75-4.90)	12726 (10023-15308)	3.98 (3.13-4.78)	0.30 (-0.05 to 0.65)	0.095	255097 (203242-315410)	158.83 (126.54-196.38)	518793 (420561-632629)	162.10 (131.41-197.67)	0.08 (0.02 to 0.14)	0.008
65-69 years	7448 (5749-9366)	6.03 (4.65-7.58)	18983 (15254-21873)	6.88 (5.53-7.93)	0.47 (0.24 to 0.70)	0	254862 (204502-311803)	206.18 (165.44-252.25)	611071 (497398-741096)	221.53 (180.32-268.67)	0.21 (0.08 to 0.33)	0.001
70-74 years	8205 (6492-10590)	9.69 (7.67-12.51)	27436 (22263-32856)	13.33 (10.82-15.96)	1.05 (0.81 to 1.29)	0	211934 (173118-259655)	250.33 (204.48-306.70)	632825 (520342-762125)	307.44 (252.79-370.25)	0.67 (0.59 to 0.75)	0
75-79 years	8979 (7524-10932)	14.59 (12.22-17.76)	30778 (26416-34918)	23.34 (20.03-26.48)	1.55 (1.22 to 1.87)	0	170044 (140332-203575)	276.25 (227.98-330.72)	495581 (409185-590169)	375.77 (310.26-447.49)	1.01 (0.89 to 1.14)	0
80-84 years	7019 (5873-8455)	19.84 (16.60-23.90)	31227 (26080-36046)	35.65 (29.78-41.16)	1.95 (1.69 to 2.21)	0	98239 (82363-117153)	277.70 (232.82-331.17)	353010 (298075-414873)	403.06 (340.33-473.69)	1.19 (1.08 to 1.31)	0
85-89 years	4040 (3466-4673)	26.74 (22.94-30.92)	25124 (20406-28320)	54.95 (44.63-61.94)	2.35 (1.94 to 2.76)	0	38393 (31865-45639)	254.07 (210.87-302.02)	185408 (156929-218788)	405.51 (343.23-478.52)	1.52 (1.38 to 1.67)	0
90-94 years	1490 (1216-1721)	34.77 (28.37-40.17)	14013 (10961-15982)	78.33 (61.27-89.34)	2.71 (2.48 to 2.94)	0	10087 (8296-12082)	235.40 (193.59-281.96)	67981 (57542-80038)	380.01 (321.65-447.41)	1.57 (1.49 to 1.65)	0
95+ years	419 (325-494)	41.13 (31.94-48.53)	5536 (3988-6452)	101.57 (73.17-118.39)	2.95 (2.62 to 3.29)	0	2228 (1716-2874)	218.80 (168.53-282.30)	18810 (15130-23439)	345.11 (277.61-430.04)	1.49 (1.42 to 1.55)	0
Sociodemographic index (SDI)												

	Deaths						Prevalence					
	1990	1990	2021	2021	1990–2021		1990	1990	2021	2021	1990–2021	
	Cases (n)	Rate (per 100 000 population)	Cases (n)	Rate (per 100 000 population)	AAPC (95%)	*P* value	Cases (n)	Rate (per 100 000 population)	Cases (n)	Rate (per 100 000 population)	AAPC (95%)	*P* value
High SDI	18327 (16874-19104)	9.83 (9.05-10.25)	79141 (68649-85444)	22.94 (19.90-24.77)	2.78 (2.45 to 3.11)	0	660592 (565380-773429)	354.27 (303.21-414.79)	1644637 (1435592-1867919)	476.69 (416.10-541.41)	0.97 (0.92 to 1.02)	0
High-middle SDI	6979 (6454-7821)	4.05 (3.74-4.53)	21470 (18633-23617)	6.19 (5.37-6.81)	1.43 (1.14 to 1.72)	0	218755 (188931-254567)	126.80 (109.51-147.55)	538470 (474771-609820)	155.32 (136.95-175.90)	0.66 (0.59 to 0.72)	0
Middle SDI	7273 (5683-10029)	4.19 (3.27-5.78)	29659 (24668-35735)	6.31 (5.25-7.61)	1.37 (1.15 to 1.60)	0	188736 (158371-224951)	108.74 (91.25-129.61)	640755 (553398-738067)	136.37 (117.78-157.08)	0.74 (0.70 to 0.77)	0
Low-middle SDI	11056 (6402-16921)	10.97 (6.35-16.79)	34618 (23517-47242)	14.36 (9.75-19.60)	0.92 (0.39 to 1.45)	0.001	147622 (122457-176178)	146.45 (121.48-174.78)	401178 (343875-464674)	166.41 (142.64-192.74)	0.41 (0.38 to 0.44)	0
Low SDI	3684 (1838-5204)	9.87 (4.93-13.95)	9395 (5633-13483)	11.45 (6.86-16.43)	0.54 (0.10 to 0.98)	0.015	38196 (31568-45718)	102.38 (84.61-122.54)	88041 (75357-102187)	107.29 (91.83-124.53)	0.15 (0.12 to 0.18)	0
Region												
Andean Latin America	1253 (901-1807)	37.34 (26.84-53.84)	5963 (4544-7555)	60.19 (45.87-76.26)	1.57 (1.14 to 2.01)	0	11359 (10098-12526)	338.47 (300.91-373.26)	64598 (59323-69497)	652.09 (598.83-701.53)	2.16 (2.11 to 2.20)	0
Australasia	252 (230-273)	6.40 (5.84-6.93)	1849 (1547-2032)	20.93 (17.51-23.01)	3.81 (3.12 to 4.49)	0	6830 (6009-7755)	173.38 (152.53-196.86)	29504 (26460-32997)	333.97 (299.52-373.52)	2.13 (2.07 to 2.19)	0
Caribbean	138 (115-169)	3.21 (2.67-3.92)	541 (458-651)	5.84 (4.95-7.03)	2.05 (1.25 to 2.86)	0	2367 (2044-2749)	54.93 (47.43-63.78)	7217 (6407-8032)	77.95 (69.20-86.75)	1.14 (1.09 to 1.20)	0
Central Asia	567 (469-671)	7.09 (5.87-8.39)	497 (406-608)	3.42 (2.79-4.18)	-2.31 (-3.16 to -1.46)	0	9963 (8757-11282)	124.57 (109.49-141.06)	18136 (16144-20271)	124.65 (110.96-139.32)	0.00 (-0.08 to 0.08)	0.943
Central Europe	1241 (1160-1338)	4.68 (4.37-5.05)	1979 (1790-2157)	5.35 (4.84-5.82)	0.43 (-0.27 to 1.14)	0.23	32265 (28002-37212)	121.66 (105.59-140.32)	45704 (40726-51629)	123.43 (109.99-139.43)	0.05 (0.00 to 0.10)	0.046
Central Latin America	866 (819-921)	6.38 (6.03-6.79)	5554 (5006-6128)	12.99 (11.71-14.33)	2.34 (2.22 to 2.46)	0	21764 (18501-25551)	160.38 (136.34-188.29)	85179 (74835-96479)	199.17 (174.99-225.60)	0.70 (0.67 to 0.72)	0
Central Sub-Saharan Africa	200 (69-452)	5.32 (1.83-12.02)	505 (175-1302)	5.60 (1.94-14.43)	0.17 (0.12 to 0.23)	0	2290 (1919-2767)	60.91 (51.03-73.59)	6071 (5162-7020)	67.28 (57.21-77.79)	0.32 (0.29 to 0.36)	0
East Asia	2411 (1808-3879)	1.62 (1.21-2.60)	7548 (4749-10033)	1.92 (1.21-2.56)	0.58 (0.37 to 0.79)	0	153157 (126464-186683)	102.82 (84.90-125.33)	507278 (432825-595940)	129.37 (110.38-151.98)	0.71 (0.56 to 0.87)	0
Eastern Europe	2399 (2242-2564)	4.91 (4.58-5.24)	977 (890-1073)	1.57 (1.43-1.73)	-3.36 (-4.64 to -2.06)	0	49159 (41256-58928)	100.55 (84.38-120.52)	28233 (23634-33096)	45.48 (38.07-53.31)	-2.54 (-2.59 to -2.48)	0

	Deaths						Prevalence					
	1990	1990	2021	2021	1990–2021		1990	1990	2021	2021	1990–2021	
	Cases (n)	Rate (per 100 000 population)	Cases (n)	Rate (per 100 000 population)	AAPC (95%)	*P* value	Cases (n)	Rate (per 100 000 population)	Cases (n)	Rate (per 100 000 population)	AAPC (95%)	*P* value
Eastern Sub-Saharan Africa	511 (175-939)	4.20 (1.44-7.72)	1089 (379-2350)	4.03 (1.40-8.69)	-0.14 (-0.23 to -0.04)	0.005	5822 (4858-6990)	47.86 (39.93-57.46)	14481 (12336-16998)	53.56 (45.63-62.87)	0.36 (0.35 to 0.38)	0
High-income Asia Pacific	4877 (4400-5259)	13.95 (12.58-15.04)	25879 (21548-28543)	36.71 (30.56-40.48)	3.37 (3.01 to 3.73)	0	205142 (172549-243237)	586.65 (493.44-695.59)	569542 (496041-652435)	807.82 (703.57-925.40)	1.03 (0.93 to 1.12)	0
High-income North America	7161 (6551-7508)	12.36 (11.31-12.96)	28573 (24935-30368)	25.39 (22.16-26.99)	2.32 (2.04 to 2.61)	0	281365 (238420-330638)	485.71 (411.58-570.77)	655461 (565506-749257)	582.45 (502.52-665.80)	0.63 (0.54 to 0.71)	0
North Africa and Middle East	722 (504-1123)	2.55 (1.78-3.97)	2408 (1761-3728)	3.16 (2.31-4.89)	0.71 (0.58 to 0.83)	0	24620 (21048-28894)	87.11 (74.47-102.23)	101507 (88750-114986)	133.15 (116.42-150.83)	1.38 (1.36 to 1.40)	0
Oceania	34 (22-58)	7.07 (4.58-12.03)	93 (54-166)	7.57 (4.36-13.46)	0.22 (0.07 to 0.36)	0.003	621 (535-720)	129.13 (111.25-149.76)	1819 (1609-2044)	147.39 (130.38-165.59)	0.42 (0.37 to 0.47)	0
South Asia	14831 (8265-23407)	15.62 (8.71-24.65)	48856 (32065-66447)	19.68 (12.91-26.76)	0.83 (0.49 to 1.17)	0	186239 (152915-224418)	196.16 (161.06-236.37)	537460 (455342-627084)	216.46 (183.39-252.56)	0.31 (0.28 to 0.34)	0
Southeast Asia	501 (246-1124)	1.18 (0.58-2.65)	1539 (790-3053)	1.34 (0.69-2.66)	0.42 (0.26 to 0.57)	0	22779 (19109-27624)	53.80 (45.13-65.24)	83298 (70468-97124)	72.71 (61.52-84.78)	0.98 (0.96 to 0.99)	0
Southern Latin America	1015 (941-1092)	12.81 (11.88-13.79)	4135 (3687-4450)	28.10 (25.05-30.24)	2.62 (1.85 to 3.40)	0	20540 (18371-22775)	259.29 (231.91-287.50)	70708 (64567-76802)	480.48 (438.75-521.89)	2.01 (1.98 to 2.05)	0
Southern Sub-Saharan Africa	400 (200-635)	9.05 (4.52-14.35)	834 (579-1100)	8.57 (5.94-11.30)	-0.19 (-0.56 to 0.17)	0.302	7928 (6599-9428)	179.17 (149.15-213.08)	16097 (13649-18831)	165.34 (140.20-193.43)	-0.28 (-0.33 to -0.22)	0
Tropical Latin America	608 (561-648)	4.01 (3.70-4.28)	3979 (3538-4278)	8.98 (7.99-9.66)	2.73 (2.38 to 3.08)	0	14008 (11719-16893)	92.51 (77.39-111.57)	38129 (32586-43912)	86.07 (73.56-99.13)	-0.22 (-0.31 to -0.12)	0
Western Europe	6265 (5826-6557)	6.45 (6.00-6.75)	29701 (26012-31944)	19.92 (17.44-21.42)	3.76 (3.34 to 4.17)	0	188999 (166448-215074)	194.62 (171.40-221.47)	419846 (376193-465977)	281.52 (252.25-312.45)	1.19 (1.12 to 1.25)	0
Western Sub-Saharan Africa	1101 (417-1743)	7.62 (2.89-12.07)	1867 (716-3263)	5.81 (2.23-10.15)	-0.87 (-0.95 to -0.79)	0	7534 (6348-9026)	52.19 (43.97-62.53)	14410 (12322-16879)	44.83 (38.33-52.51)	-0.49 (-0.52 to -0.47)	0

Data in parentheses are 95% uncertainty intervals for cases, deaths, and prevalence, and 95% CIs for AAPCs. UI=uncertainty interval. CI=confidence interval. AAPC=average annual percentage change.
