# Supplementary material for: Global burden, trends and projections analysis of interstitial lung disease and pulmonary sarcoidosis in elderly adults (aged 55+ Years) based on GBD 2021
Source: PLoS One. 2026 Apr 20;21(4):e0347482. doi: 10.1371/journal.pone.0347482 (PMC13095001; doi:10.1371/journal.pone.0347482)
Supplement: S2 Table — Abbreviations: ILD&PS, Interstitial lung disease and pulmonary sarcoidosis; AAPC, average annual per centage change; DALYs, disability-adjusted life-years; UI, uncertainty interval. (PDF) [file pone.0347482.s002.pdf]

	Deaths						Prevalence					
	Cases（n),1990	Deaths（per 100 000 population), 1990	Cases（n),2021	Deaths（per 100 000 population), 2021	AAPC, 1990–2021	P value	Cases（n),1990	Prevalence（per 100 000 population), 1990	Cases（n),2021	Prevalence（per 100 000 population), 2021	AAPC, 1990–2021	P value
Afghanistan	3（0-12)	0.26（0.00-0.97)	15（1-50)	1.21（0.10-4.06)	5.19（4.85 to 5.54)	0	776（652-939)	63.84（53.64-77.30)	960（825-1114)	78.14（67.13-90.63)	0.67（0.59 to 0.74)	0
Albania	19（12-28)	5.35（3.36-8.14)	33（17-58)	4.23（2.20-7.36)	-0.77（-1.00 to -0.54)	0	405（352-465)	116.58（101.31-134.02)	949（830-1076)	120.71（105.59-136.89)	0.12（0.07 to 0.16)	0
Algeria	3（0-10)	0.14（0.00-0.48)	48（4-181)	0.80（0.06-2.98)	5.77（5.48 to 6.06)	0	1908（1602-2275)	91.08（76.46-108.59)	7205（6237-8364)	118.63（102.70-137.71)	0.86（0.80 to 0.92)	0
American Samoa	0（0-0)	3.59（2.10-5.18)	0（0-0)	2.66（1.77-4.49)	-0.93（-1.33 to -0.52)	0	3（3-4)	90.40（78.72-104.84)	6（6-7)	74.36（65.42-84.72)	-0.63（-0.64 to -0.62)	0
Andorra	1（1-2)	10.91（5.91-18.39)	3（1-6)	11.41（3.29-22.56)	0.08（-0.40 to 0.57)	0.737	24（21-28)	246.16（213.37-282.40)	74（65-83)	277.82（246.16-312.91)	0.40（0.33 to 0.48)	0
Angola	29（10-59)	4.59（1.63-9.46)	76（28-147)	3.93（1.44-7.62)	-0.50（-0.77 to -0.24)	0	325（275-389)	52.21（44.20-62.40)	1287（1101-1494)	66.57（56.96-77.25)	0.79（0.76 to 0.82)	0

Antigua and Barbuda	0 (0-0)	2.87 (2.55-3.23)	1 (1-1)	5.13 (4.70-5.64)	1.83 (1.12 to 2.54)	0	4 (4-5)	47.78 (41.03-55.66)	14 (12-16)	72.75 (63.86-82.44)	1.36 (1.31 to 1.41)	0
Argentina	645 (581-713)	11.58 (10.44-12.81)	1878 (1667-2057)	20.12 (17.86-22.04)	1.85 (0.71 to 3.01)	0.001	13176 (11687-14722)	236.79 (210.03-264.57)	34477 (31592-37567)	369.40 (338.48-402.50)	1.44 (1.41 to 1.47)	0
Armenia	41 (37-45)	8.43 (7.56-9.14)	52 (45-59)	6.63 (5.75-7.54)	-0.57 (-2.83 to 1.75)	0.628	720 (628-816)	147.89 (128.94-167.74)	1308 (1175-1455)	166.31 (149.33-184.94)	0.39 (0.30 to 0.47)	0
Australia	206 (187-224)	6.27 (5.69-6.83)	1587 (1325-1751)	21.43 (17.88-23.63)	3.93 (3.13 to 4.74)	0	5299 (4687-5978)	161.48 (142.84-182.18)	25616 (22904-28597)	345.79 (309.18-386.04)	2.48 (2.44 to 2.53)	0
Austria	64 (59-69)	3.28 (3.02-3.55)	241 (208-268)	8.15 (7.04-9.07)	3.19 (2.39 to 3.99)	0	2885 (2562-3251)	148.24 (131.65-167.05)	4823 (4333-5333)	163.22 (146.64-180.46)	0.30 (0.24 to 0.36)	0
Azerbaijan	45 (19-90)	5.19 (2.15-10.36)	55 (25-100)	2.89 (1.28-5.22)	-1.89 (-2.09 to -1.69)	0	924 (808-1058)	105.92 (92.60-121.26)	2097 (1820-2392)	109.46 (94.97-124.87)	0.10 (0.04 to 0.16)	0.002229
Bahamas	1 (1-2)	5.67 (5.04-6.28)	8 (7-10)	11.34 (9.35-13.66)	2.05 (1.00 to 3.12)	0	24 (21-27)	97.66 (85.17-109.88)	88 (78-97)	122.07 (108.70-134.88)	0.72 (0.69 to 0.76)	0
Bahrain	2 (2-4)	8.08 (5.65-14.45)	14 (8-20)	8.41 (4.88-12.27)	0.05 (-0.18 to 0.28)	0.666	37 (31-43)	130.55 (110.80-152.73)	341 (301-387)	210.22 (185.36-238.07)	1.53 (1.42 to 1.65)	0

Bangladesh	1143 (614-1828)	15.09 (8.11-24.14)	3072 (1974-4748)	13.15 (8.45-20.32)	-0.40 (-0.75 to -0.05)	0.027	12300 (10598-14189)	162.38 (139.92-187.32)	52461 (45869-58712)	224.52 (196.31-251.27)	1.04 (1.00 to 1.08)	0
Barbados	3 (2-3)	5.44 (4.86-5.96)	9 (7-11)	9.96 (8.06-12.00)	2.10 (1.65 to 2.55)	0	35 (31-40)	75.59 (66.41-86.37)	96 (86-105)	104.94 (94.37-115.47)	1.07 (1.04 to 1.10)	0
Belarus	212 (181-240)	9.15 (7.83-10.37)	37 (30-44)	1.27 (1.04-1.53)	-6.01 (-6.38 to -5.65)	0	4007 (3496-4582)	173.10 (151.02-197.95)	1924 (1670-2191)	66.90 (58.04-76.17)	-3.03 (-3.06 to -2.99)	0
Belgium	154 (140-165)	5.88 (5.36-6.31)	549 (470-605)	14.51 (12.44-16.00)	2.95 (2.13 to 3.78)	0	3363 (2975-3800)	128.28 (113.45-144.95)	7568 (6818-8400)	200.17 (180.33-222.19)	1.45 (1.34 to 1.56)	0
Belize	2 (1-2)	10.49 (8.77-12.84)	10 (8-11)	19.24 (16.23-22.69)	1.93 (0.14 to 3.75)	0.035	21 (18-24)	136.36 (118.12-153.77)	127 (115-139)	253.01 (228.95-277.30)	2.00 (1.92 to 2.08)	0
Benin	31 (11-56)	9.75 (3.52-17.54)	49 (17-108)	5.92 (2.11-13.11)	-1.62 (-1.85 to -1.40)	0	151 (128-177)	47.13 (40.08-55.35)	324 (277-378)	39.12 (33.45-45.65)	-0.60 (-0.66 to -0.54)	0
Bermuda	1 (1-2)	13.57 (11.41-15.87)	5 (4-6)	21.76 (18.27-26.08)	1.55 (0.51 to 2.61)	0.004	20 (18-22)	190.65 (170.35-210.32)	69 (63-75)	296.75 (270.83-323.17)	1.43 (1.40 to 1.47)	0
Bhutan	5 (3-7)	12.05 (7.16-18.50)	17 (10-26)	16.65 (10.46-26.34)	1.06 (0.95 to 1.18)	0	58 (49-67)	146.19 (123.51-169.40)	288 (254-322)	289.33 (254.70-322.92)	2.23 (2.18 to 2.27)	0

Bolivia （Plurinational State of)	186 （89-360)	35.40 （16.88-68.64)	670 （413-987)	43.36 （26.70-63.84)	0.67 （0.59 to 0.76)	0	1636 （1435-1828)	312.03 （273.61-348.73)	8100 （7285-8925)	524.13 （471.36-577.49)	1.70 （1.66 to 1.73)	0
Bosnia and Herzegovina	25 （14-40)	3.39 （1.94-5.43)	31 （17-51)	2.80 （1.58-4.70)	-0.54 （-0.86 to -0.21)	0.001	643 （550-758)	87.64 （74.96-103.39)	967 （845-1112)	88.30 （77.19-101.54)	0.05 （-0.09 to 0.20)	0.468411
Botswana	8 （2-16)	8.35 （1.98-17.03)	14 （4-30)	5.75 （1.44-12.50)	-1.23 （-1.85 to -0.59)	0	119 （101-139)	128.18 （108.88-149.39)	367 （321-417)	151.31 （132.34-171.84)	0.53 （0.50 to 0.56)	0
Brazil	592 （547-634)	4.00 （3.70-4.29)	3914 （3482-4210)	9.04 （8.04-9.72)	2.75 （2.40 to 3.11)	0	13825 （11560-16682)	93.55 （78.22-112.88)	37389 （31917-43118)	86.33 （73.70-99.56)	-0.25 （-0.34 to -0.15)	0
Brunei Darussalam	2 （2-4)	15.43 （9.87-22.51)	6 （4-8)	9.87 （6.63-13.59)	-1.50 （-1.96 to -1.03)	0	110 （98-125)	705.09 （625.25-795.44)	324 （292-359)	541.42 （487.87-598.98)	-0.85 （-0.95 to -0.76)	0
Bulgaria	42 （36-49)	1.85 （1.58-2.16)	60 （50-71)	2.52 （2.10-3.00)	1.28 （0.58 to 1.99)	0	1664 （1446-1936)	73.53 （63.89-85.53)	1330 （1153-1538)	55.96 （48.53-64.71)	-0.88 （-0.92 to -0.84)	0
Burkina Faso	38 （13-66)	5.24 （1.77-9.18)	62 （23-122)	4.09 （1.53-8.13)	-0.78 （-0.94 to -0.61)	0	265 （220-315)	36.59 （30.39-43.50)	463 （389-548)	30.83 （25.89-36.47)	-0.55 （-0.59 to -0.51)	0
Burundi	21 （7-38)	5.51 （1.80-10.05)	33 （11-73)	4.08 （1.34-9.10)	-0.95 （-1.14 to -0.77)	0	208 （173-247)	55.23 （45.89-65.59)	438 （369-512)	54.73 （46.05-63.94)	-0.03 （-0.05 to 0.00)	0.071194

Cabo Verde	5 （1-12)	13.55 （3.44-29.19)	4 （2-7)	5.21 （2.00-9.69)	-3.22 （-3.91 to -2.52)	0	11 （9-13)	27.66 （22.62-33.03)	28 （23-33)	36.53 （30.60-43.24)	0.91 （0.88 to 0.95)	0
Cambodia	5 （1-15)	0.61 （0.09-2.02)	15 （3-47)	0.69 （0.12-2.17)	0.38 （0.34 to 0.43)	0	270 （222-330)	36.46 （29.96-44.58)	1122 （936-1327)	51.62 （43.07-61.06)	1.13 （1.11 to 1.14)	0
Cameroon	72 （28-127)	9.75 （3.77-17.17)	131 （48-250)	6.42 （2.35-12.30)	-1.33 （-1.46 to -1.21)	0	382 （321-453)	51.56 （43.40-61.23)	824 （709-971)	40.50 （34.82-47.68)	-0.78 （-0.81 to -0.74)	0
Canada	617 （560-670)	11.30 （10.26-12.28)	3046 （2673-3317)	24.82 （21.78-27.03)	2.64 （1.81 to 3.48)	0	18058 （16416-19973)	330.80 （300.71-365.87)	67566 （61057-73984)	550.61 （497.57-602.91)	1.65 （1.62 to 1.68)	0
Central African Republic	11 （3-22)	5.60 （1.79-11.70)	18 （7-40)	5.06 （1.86-11.18)	-0.34 （-0.51 to -0.17)	0	105 （87-127)	55.86 （45.91-67.43)	183 （154-216)	51.24 （42.95-60.48)	-0.28 （-0.31 to -0.25)	0
Chad	41 （14-74)	8.87 （3.08-16.05)	61 （23-121)	6.61 （2.52-13.02)	-0.94 （-1.03 to -0.84)	0	197 （165-233)	42.58 （35.61-50.18)	352 （299-413)	37.88 （32.21-44.47)	-0.38 （-0.42 to -0.34)	0
Chile	321 （298-344)	19.07 （17.70-20.44)	2079 （1851-2240)	46.43 （41.34-50.03)	2.89 （2.14 to 3.65)	0	6197 （5626-6761)	368.50 （334.53-402.01)	33613 （30682-36847)	750.69 （685.23-822.91)	2.33 （2.28 to 2.37)	0
China	2337 （1745-3764)	1.63 （1.22-2.62)	7068 （4279-9521)	1.87 （1.13-2.51)	0.44 （0.26 to 0.62)	0	149285 （123096-	104.02 （85.77-126.98)	491498 （418647-	129.70 （110.47-152.61)	0.73 （0.52 to 0.94)	0

							182234)		578309)			
Colombia	61 (56-66)	2.13 (1.96-2.29)	718 (597-848)	7.51 (6.24-8.86)	4.24 （3.56 to 4.92)	0	2107 (1787-2488)	73.19 (62.06-86.43)	12069 (10747-13482)	126.20 (112.37-140.98)	1.78 （1.75 to 1.81)	0
Comoros	2 (0-3)	4.69 (1.52-9.20)	3 (1-7)	3.92 (1.39-8.71)	-0.58 （-0.69 to -0.47)	0	19 (16-22)	57.29 (48.10-67.67)	46 (39-53)	56.70 (48.78-66.12)	-0.03 （-0.08 to 0.01)	0.171443
Congo	11 (4-25)	6.31 (2.03-14.00)	22 (9-44)	5.14 (1.98-10.11)	-0.68 （-0.78 to -0.58)	0	109 (91-130)	61.33 (51.00-72.98)	315 (272-363)	72.39 (62.51-83.63)	0.53 （0.49 to 0.57)	0
Cook Islands	0 (0-0)	4.11 (2.68-7.10)	0 (0-0)	3.45 (1.68-6.83)	-0.58 （-0.68 to -0.49)	0	3 (2-3)	120.68 (106.18-138.02)	6 (5-7)	131.19 (116.09-146.86)	0.25 （0.18 to 0.32)	0
Costa Rica	27 (24-30)	9.59 (8.53-10.45)	158 (137-178)	16.50 (14.36-18.63)	2.07 （0.98 to 3.17)	0	672 (600-746)	236.28 (210.93-262.14)	3207 (2921-3507)	334.97 (305.03-366.26)	1.12 （1.09 to 1.16)	0
Côte d'Ivoire	13 (12-14)	1.20 (1.11-1.30)	29 (25-33)	1.95 (1.70-2.21)	-1.12 （-1.26 to -0.98)	0	734 (613-866)	65.96 (55.05-77.77)	1107 (981-1246)	74.25 (65.78-83.54)	0.37 （0.30 to 0.45)	0
Croatia	13 (12-14)	0.77 (0.70-0.85)	59 (51-67)	1.72 (1.49-1.95)	1.59 （0.83 to 2.34)	0	644 (529-783)	38.00 (31.23-46.23)	1415 (1208-1653)	41.42 (35.34-48.38)	0.28 （0.24 to 0.31)	0

Cuba	46 (29-73)	33.00 (21.05-52.04)	86 (58-120)	24.59 (16.55-34.29)	2.71 (1.71 to 3.73)	0	479 (415-547)	342.88 (297.01-391.26)	1261 (1128-1408)	360.85 (322.89-402.72)	0.16 (0.10 to 0.22)	1.00E-06
Cyprus	75 (65-89)	3.18 (2.73-3.76)	335 (288-388)	9.59 (8.24-11.11)	-1.03 (-1.38 to -0.68)	0	1713 (1473-1987)	72.34 (62.21-83.93)	5390 (4824-6027)	154.29 (138.08-172.52)	2.50 (2.38 to 2.62)	0
Czechia	52 (19-93)	8.18 (2.94-14.57)	104 (36-208)	5.78 (2.01-11.61)	3.74 (2.52 to 4.98)	0	322 (269-380)	50.19 (41.96-59.28)	749 (644-869)	41.75 (35.93-48.46)	-0.58 (-0.64 to -0.52)	0
Democratic People's Republic of Korea	42 (21-87)	1.56 (0.78-3.26)	110 (57-217)	1.95 (1.02-3.85)	0.73 (0.66 to 0.81)	0	2040 (1697-2421)	76.67 (63.75-90.98)	4384 (3781-5070)	77.79 (67.10-89.96)	0.04 (-0.04 to 0.12)	0.320381
Democratic Republic of the Congo	141 (48-340)	5.36 (1.81-12.89)	377 (124-1096)	6.24 (2.06-18.14)	0.49 (0.37 to 0.62)	0	1669 (1397-2022)	63.21 (52.92-76.56)	4072 (3463-4746)	67.41 (57.33-78.57)	0.22 (0.14 to 0.30)	0
Denmark	83 (76-91)	6.37 (5.80-6.98)	308 (267-348)	16.01 (13.86-18.09)	3.01 (1.97 to 4.05)	0	2550 (2270-2851)	195.43 (174.01-218.54)	4831 (4369-5321)	250.91 (226.92-276.38)	0.81 (0.77 to 0.85)	0
Djibouti	1 (0-1)	3.46 (1.20-6.47)	3 (1-6)	2.84 (0.98-5.99)	-0.62 (-0.71 to -0.53)	0	12 (10-14)	55.99 (46.43-67.07)	64 (54-74)	61.56 (52.17-71.60)	0.31 (0.27 to 0.35)	0
Dominica	0 (0-1)	4.14 (3.00-6.39)	1 (0-1)	5.26 (3.29-7.47)	0.79 (0.66 to 0.92)	0	6 (5-7)	60.32 (52.14-70.14)	12 (11-14)	82.59 (73.10-92.32)	1.02 (1.00 to 1.04)	0

Dominican Republic	9 (5-18)	1.40 (0.80-2.93)	37 (20-66)	2.24 (1.19-3.92)	1.53 (0.94 to 2.12)	0	210 (175-252)	34.35 (28.62-41.23)	904 (783-1037)	54.07 (46.86-62.03)	1.48 (1.45 to 1.52)	0
Ecuador	134 (118-151)	15.54 (13.74-17.53)	1116 (920-1355)	40.31 (33.25-48.96)	3.07 (2.04 to 4.11)	0	1565 (1387-1751)	182.01 (161.28-203.64)	10508 (9531-11404)	379.69 (344.37-412.08)	2.42 (2.33 to 2.52)	0
Egypt	122 (88-176)	2.72 (1.96-3.94)	184 (128-262)	1.66 (1.16-2.37)	-1.60 (-2.06 to -1.14)	0	3375 (2819-4059)	75.48 (63.05-90.78)	12842 (11003-15099)	116.05 (99.43-136.45)	1.40 (1.38 to 1.42)	0
El Salvador	35 (24-57)	7.07 (4.99-11.69)	100 (60-138)	9.78 (5.89-13.47)	1.03 (0.77 to 1.29)	0	547 (477-621)	111.84 (97.54-127.08)	2254 (2025-2481)	220.38 (198.02-242.59)	2.23 (2.18 to 2.27)	0
Equatorial Guinea	2 (1-4)	5.69 (1.90-11.41)	4 (1-8)	4.68 (1.60-10.18)	-0.61 (-0.91 to -0.32)	0	14 (12-17)	43.75 (36.13-52.90)	70 (60-81)	87.99 (75.50-101.98)	2.30 (2.17 to 2.44)	0
Eritrea	7 (2-13)	3.96 (1.15-7.27)	17 (6-30)	3.79 (1.45-6.76)	-0.14 (-0.24 to -0.04)	0.006	77 (63-93)	43.67 (35.86-52.55)	223 (191-259)	50.61 (43.26-58.76)	0.46 (0.43 to 0.50)	0
Estonia	50 (45-57)	14.03 (12.47-15.79)	9 (8-11)	2.10 (1.79-2.42)	-5.78 (-7.91 to -3.59)	0	251 (218-286)	70.03 (60.72-79.92)	406 (367-455)	92.85 (83.83-104.05)	0.92 (0.88 to 0.97)	0
Eswatini	4 (1-8)	8.94 (2.26-18.77)	7 (2-13)	7.20 (2.08-13.85)	-0.69 (-0.77 to -0.61)	0	55 (47-64)	122.26 (104.65-141.93)	104 (91-118)	115.39 (100.16-130.73)	-0.19 (-0.22 to -0.15)	0

Ethiopia	122 (34-221)	3.78 (1.05-6.87)	235 (70-513)	3.43 (1.02-7.49)	-0.29 (-0.44 to -0.14)	0	1242 (1013-1532)	38.66 (31.52-47.67)	3032 (2546-3587)	44.29 (37.19-52.40)	0.45 (0.41 to 0.49)	0
Fiji	1 (1-2)	2.00 (1.38-2.80)	3 (2-4)	2.10 (1.23-3.04)	0.16 (-0.01 to 0.32)	0.069	33 (29-40)	59.39 (50.81-70.15)	83 (72-96)	61.01 (52.88-70.57)	0.08 (0.05 to 0.11)	1.00E-06
Finland	89 (78-100)	7.49 (6.60-8.46)	436 (371-484)	21.64 (18.43-24.06)	3.46 (2.71 to 4.21)	0	2258 (1992-2550)	190.14 (167.74-214.78)	6083 (5465-6726)	302.24 (271.51-334.18)	1.52 (1.46 to 1.58)	0
France	830 (745-900)	5.97 (5.35-6.47)	3026 (2522-3401)	13.68 (11.41-15.38)	2.77 (2.27 to 3.27)	0	21179 (18791-23967)	152.19 (135.03-172.22)	48367 (44030-53208)	218.76 (199.14-240.65)	1.17 (1.12 to 1.23)	0
Gabon	6 (2-12)	6.49 (2.27-12.76)	8 (3-18)	4.57 (1.64-10.24)	-1.10 (-1.15 to -1.05)	0	67 (57-80)	68.72 (58.13-81.90)	145 (125-166)	81.04 (69.91-93.12)	0.54 (0.50 to 0.57)	0
Gambia	5 (2-9)	9.20 (3.15-16.90)	12 (4-25)	7.49 (2.87-15.92)	-0.70 (-1.10 to -0.30)	0.001	27 (22-32)	48.33 (40.41-56.93)	62 (53-71)	39.92 (34.35-46.00)	-0.62 (-0.65 to -0.59)	0
Georgia	30 (24-37)	2.70 (2.20-3.30)	32 (27-38)	3.07 (2.59-3.61)	0.50 (-2.24 to 3.31)	0.725	1050 (921-1193)	94.53 (82.95-107.44)	1048 (938-1163)	99.79 (89.36-110.79)	0.20 (-0.02 to 0.43)	0.079635
Germany	1381 (1213-1552)	6.58 (5.78-7.40)	4266 (3698-4711)	13.54 (11.73-14.95)	2.46 (2.16 to 2.77)	0	34079 (30371-38046)	162.41 (144.74-181.32)	67728 (61098-74614)	214.92 (193.88-236.77)	0.92 (0.85 to 0.99)	0

Ghana	58 (21-96)	5.64 (2.03-9.37)	163 (67-274)	5.97 (2.44-10.07)	0.19 (0.09 to 0.28)	0	645 (547-757)	63.17 (53.56-74.08)	1796 (1563-2036)	65.87 (57.33-74.67)	0.13 (0.04 to 0.22)	0.006444
Greece	57 (51-62)	2.12 (1.91-2.32)	527 (461-579)	14.35 (12.53-15.76)	6.43 (3.25 to 9.71)	0	2198 (1875-2566)	82.22 (70.12-95.98)	6050 (5366-6729)	164.60 (146.00-183.09)	2.29 (2.20 to 2.37)	0
Greenland	1 (1-1)	18.58 (9.42-25.93)	2 (1-3)	13.49 (5.30-20.97)	-0.92 (-1.21 to -0.64)	0	22 (20-25)	405.30 (354.15-461.74)	60 (53-66)	440.20 (392.62-489.73)	0.27 (0.18 to 0.36)	0
Grenada	0 (0-1)	3.82 (3.25-4.35)	1 (1-2)	7.26 (6.26-8.21)	2.10 (1.04 to 3.17)	0	7 (6-8)	58.52 (50.96-66.73)	18 (16-21)	91.13 (81.05-102.62)	1.43 (1.40 to 1.47)	0
Guam	2 (1-2)	14.43 (10.07-19.16)	3 (2-4)	7.23 (5.20-11.45)	-2.20 (-2.58 to -1.81)	0	41 (37-46)	317.87 (284.75-353.46)	97 (87-107)	256.61 (231.64-282.71)	-0.69 (-0.75 to -0.63)	0
Guatemala	52 (43-62)	9.02 (7.52-10.83)	275 (234-319)	15.02 (12.78-17.43)	1.25 (-0.32 to 2.84)	0.119	769 (684-854)	133.72 (118.92-148.56)	3377 (3075-3694)	184.43 (167.96-201.77)	1.05 (0.95 to 1.15)	0
Guinea	49 (19-87)	8.90 (3.38-15.67)	63 (23-133)	6.92 (2.56-14.51)	-0.79 (-0.87 to -0.71)	0	263 (223-310)	47.59 (40.27-55.94)	358 (309-413)	39.21 (33.82-45.24)	-0.62 (-0.65 to -0.60)	0
Guinea-Bissau	7 (2-13)	10.57 (3.44-19.78)	8 (3-14)	6.60 (2.68-11.92)	-1.51 (-1.59 to -1.42)	0	30 (25-35)	46.26 (38.65-54.53)	42 (36-49)	36.43 (31.04-42.92)	-0.77 (-0.79 to -0.74)	0

Guyana	4 （4-5)	6.73 （5.84-7.64)	8 （6-10)	6.75 （5.23-8.54)	0.30 （-1.08 to 1.69)	0.675	36 （32-41)	59.00 （51.42-67.54)	94 （83-105)	83.11 （73.69-93.09)	1.12 （1.09 to 1.16)	0
Haiti	33 （13-55)	6.09 （2.38-10.12)	91 （43-172)	7.68 （3.59-14.55)	0.78 （0.66 to 0.90)	0	297 （252-347)	55.02 （46.71-64.27)	805 （692-921)	67.95 （58.42-77.73)	0.69 （0.66 to 0.73)	0
Honduras	30 （19-47)	9.01 （5.77-14.04)	158 （76-231)	15.01 （7.21-22.00)	1.68 （1.43 to 1.93)	0	451 （385-519)	135.23 （115.47-155.70)	2396 （2111-2727)	227.80 （200.77-259.30)	1.70 （1.68 to 1.73)	0
Hungary	111 （103-121)	4.31 （4.00-4.69)	223 （194-253)	7.02 （6.09-7.97)	1.77 （1.11 to 2.44)	0	2735 （2425-3075)	106.17 （94.16-119.40)	4165 （3773-4592)	130.98 （118.64-144.41)	0.69 （0.63 to 0.75)	0
Iceland	2 （2-2)	3.92 （3.46-4.30)	12 （10-14)	12.22 （10.42-13.87)	3.83 （2.90 to 4.78)	0	66 （57-75)	138.32 （120.49-157.39)	193 （175-214)	198.15 （179.54-219.69)	1.17 （1.13 to 1.22)	0
India	12247 （6731-19619)	15.97 （8.78-25.59)	42474 （27506-59623)	21.13 （13.68-29.66)	1.00 （0.61 to 1.39)	0	154373 （125883-187607)	201.35 （164.19-244.70)	446277 （374669-524070)	221.98 （186.36-260.67)	0.31 （0.29 to 0.33)	0
Indonesia	136 （23-407)	0.84 （0.15-2.52)	393 （77-1109)	0.94 （0.18-2.65)	0.34 （0.23 to 0.44)	0	9119 （7546-11235)	56.45 （46.71-69.54)	29048 （24314-34545)	69.36 （58.06-82.49)	0.67 （0.65 to 0.69)	0
Iran （Islamic Republic of)	3 （1-6)	0.07 （0.02-0.13)	27 （4-50)	0.21 （0.03-0.39)	3.77 （3.51 to 4.03)	0	2679 （2186-3316)	58.10 （47.41-71.93)	8735 （7429-10388)	67.24 （57.19-79.96)	0.47 （0.45 to 0.49)	0

Iraq	39 (25-63)	3.05 (1.96-5.02)	125 (77-180)	3.21 (1.98-4.63)	0.17 (0.04 to 0.30)	0.008	894 (755-1057)	70.82 (59.78-83.74)	3958 (3423-4572)	101.88 (88.11-117.67)	1.19 (1.12 to 1.25)	0
Ireland	80 (74-87)	11.79 (10.91-12.85)	383 (322-438)	29.21 (24.56-33.38)	3.18 (2.83 to 3.53)	0	1615 (1445-1798)	237.29 (212.23-264.06)	6397 (5739-7077)	487.44 (437.32-539.26)	2.37 (2.31 to 2.42)	0
Israel	46 (42-51)	5.68 (5.13-6.25)	182 (155-202)	9.12 (7.78-10.09)	1.68 (1.21 to 2.15)	0	1093 (955-1243)	134.79 (117.81-153.35)	3285 (2954-3650)	164.38 (147.82-182.64)	0.65 (0.60 to 0.70)	0
Italy	174 (162-182)	1.14 (1.06-1.20)	3182 (2717-3469)	14.01 (11.96-15.27)	8.76 (6.80 to 10.76)	0	24361 (20346-29321)	160.07 (133.69-192.66)	59070 (50821-68149)	260.03 (223.72-299.99)	1.58 (1.42 to 1.75)	0
Jamaica	6 (5-7)	2.03 (1.81-2.25)	28 (22-35)	5.31 (4.09-6.66)	3.33 (1.26 to 5.43)	0.001	128 (110-150)	43.49 (37.29-50.90)	320 (283-362)	60.57 (53.51-68.36)	1.07 (1.02 to 1.11)	0
Japan	4434 (4083-4630)	14.97 (13.79-15.63)	23799 (19665-26116)	45.59 (37.67-50.03)	3.85 (3.43 to 4.27)	0	191540 (159785-228011)	646.80 (539.57-769.96)	473976 (406414-548927)	907.98 (778.56-1051.57)	1.09 (0.97 to 1.20)	0
Jordan	20 (13-34)	9.44 (5.93-15.99)	106 (70-152)	8.44 (5.56-12.06)	-0.32 (-0.60 to -0.04)	0.023	494 (433-560)	229.78 (201.21-260.35)	3619 (3239-4019)	287.85 (257.64-319.65)	0.73 (0.70 to 0.76)	0
Kazakhstan	80 (68-92)	3.82 (3.26-4.40)	117 (88-155)	3.68 (2.76-4.88)	-0.39 (-1.53 to 0.77)	0.51	1535 (1330-1771)	73.39 (63.60-84.68)	3659 (3237-4117)	115.30 (101.99-129.74)	1.45 (1.35 to 1.55)	0

Kenya	57 (14-168)	4.24 (1.04-12.64)	188 (48-617)	5.02 (1.28-16.49)	0.52 (0.36 to 0.67)	0	832 (691-1013)	62.45 (51.83-76.03)	2304 (1951-2745)	61.58 (52.16-73.37)	-0.05 (-0.08 to -0.02)	0.001909
Kiribati	0 (0-1)	4.81 (2.20-8.72)	1 (0-1)	4.35 (2.21-8.42)	-0.33 (-0.38 to -0.28)	0	5 (4-5)	76.14 (65.98-88.07)	9 (8-11)	74.88 (65.72-85.39)	-0.05 (-0.07 to -0.03)	0
Kuwait	10 (9-11)	10.71 (9.33-11.98)	42 (34-49)	9.03 (7.31-10.61)	-0.59 (-2.43 to 1.28)	0.535	152 (133-172)	166.31 (145.88-188.13)	975 (875-1090)	209.20 (187.58-233.71)	0.73 (0.50 to 0.97)	0
Kyrgyzstan	22 (18-27)	4.36 (3.54-5.32)	7 (6-9)	0.83 (0.67-1.04)	-5.65 (-8.24 to -2.99)	0	307 (263-361)	59.60 (51.13-70.09)	373 (311-443)	44.06 (36.79-52.43)	-0.99 (-1.06 to -0.92)	0
Lao People's Democratic Republic	3 (0-11)	0.96 (0.11-3.17)	7 (1-22)	0.91 (0.14-2.83)	-0.17 (-0.23 to -0.10)	0	160 (132-192)	46.61 (38.48-55.88)	502 (427-581)	64.99 (55.18-75.16)	1.08 (1.05 to 1.10)	0
Latvia	124 (111-138)	19.90 (17.82-22.13)	13 (11-15)	1.98 (1.68-2.29)	-6.96 (-8.24 to -5.66)	0	696 (612-790)	111.33 (97.96-126.39)	476 (420-539)	72.34 (63.77-81.89)	-1.37 (-1.53 to -1.21)	0
Lebanon	30 (14-56)	7.92 (3.75-14.83)	112 (79-169)	11.45 (8.08-17.28)	1.22 (1.04 to 1.40)	0	354 (298-431)	93.44 (78.51-113.64)	1299 (1129-1510)	132.51 (115.21-154.04)	1.14 (1.11 to 1.17)	0
Lesotho	11 (2-21)	7.57 (1.74-15.16)	12 (3-25)	6.79 (1.62-14.03)	-0.35 (-0.59 to -0.12)	0.004	158 (134-185)	113.03 (96.01-132.04)	206 (180-234)	114.05 (99.56-129.72)	0.02 (-0.02 to 0.06)	0.241846

Liberia	17 (6-33)	9.24 (3.14-17.52)	21 (7-46)	6.30 (2.06-14.06)	-1.29 (-1.43 to -1.15)	0	85 (71-102)	45.15 (37.46-54.11)	128 (108-153)	38.82 (32.68-46.39)	-0.50 (-0.58 to -0.42)	0
Libya	1 (0-2)	0.20 (0.00-0.74)	11 (1-44)	1.29 (0.08-5.21)	6.19 (5.91 to 6.47)	0	307 (259-366)	98.78 (83.17-117.72)	865 (745-1005)	103.39 (89.02-120.12)	0.14 (0.06 to 0.23)	0.000501
Lithuania	60 (51-69)	7.52 (6.48-8.75)	10 (8-11)	0.99 (0.86-1.12)	-6.39 (-7.62 to -5.15)	0	446 (378-518)	56.29 (47.71-65.40)	523 (455-594)	54.07 (47.05-61.38)	-0.13 (-0.23 to -0.03)	0.011985
Luxembourg	4 (4-4)	4.19 (3.83-4.56)	20 (17-22)	11.18 (9.84-12.57)	3.24 (2.74 to 3.75)	0	119 (105-135)	127.37 (112.56-145.00)	326 (293-361)	183.19 (164.95-203.15)	1.18 (1.13 to 1.24)	0
Madagascar	56 (21-108)	6.65 (2.43-12.74)	110 (41-227)	5.93 (2.24-12.30)	-0.34 (-0.44 to -0.25)	0	500 (420-593)	59.18 (49.74-70.24)	1220 (1039-1413)	66.05 (56.29-76.51)	0.36 (0.33 to 0.40)	0
Malawi	22 (8-43)	3.56 (1.20-6.90)	49 (18-102)	4.20 (1.51-8.71)	0.57 (0.41 to 0.73)	0	282 (236-337)	44.81 (37.53-53.65)	593 (501-693)	50.74 (42.84-59.29)	0.41 (0.39 to 0.43)	0
Malaysia	54 (38-80)	3.66 (2.60-5.45)	197 (135-268)	4.01 (2.76-5.47)	0.24 (0.01 to 0.46)	0.037	1122 (967-1302)	75.99 (65.53-88.23)	5824 (5149-6628)	118.78 (105.02-135.18)	1.46 (1.42 to 1.50)	0
Maldives	3 (2-6)	21.79 (11.32-37.67)	8 (5-11)	14.58 (9.83-20.42)	-1.31 (-1.84 to -0.78)	0	52 (44-60)	337.02 (289.30-388.74)	295 (263-329)	542.22 (483.25-605.14)	1.54 (1.46 to 1.63)	0

Mali	78 (26-138)	11.62 (3.88-20.63)	149 (50-319)	10.31 (3.43-22.02)	-0.37 (-0.49 to -0.26)	0	345 (288-411)	51.56 (43.11-61.49)	774 (671-881)	53.45 (46.38-60.89)	0.12 (0.11 to 0.14)	0
Malta	7 (7-8)	10.03 (8.99-11.00)	46 (39-53)	29.41 (24.96-33.43)	3.34 (2.92 to 3.77)	0	171 (153-191)	236.38 (211.52-263.41)	663 (601-726)	419.99 (380.87-459.87)	1.88 (1.82 to 1.94)	0
Marshall Islands	0 (0-0)	5.48 (2.88-10.33)	0 (0-1)	4.23 (1.97-8.60)	-0.84 (-0.96 to -0.72)	0	3 (3-4)	121.97 (105.64-139.70)	7 (6-8)	122.61 (108.30-138.50)	0.00 (-0.07 to 0.08)	0.976466
Mauritania	16 (6-27)	9.68 (3.47-16.48)	21 (8-42)	5.99 (2.24-11.62)	-1.54 (-1.69 to -1.38)	0	79 (66-93)	47.63 (40.04-56.16)	158 (137-184)	44.15 (38.15-51.39)	-0.25 (-0.30 to -0.19)	0
Mauritius	16 (14-17)	12.88 (11.78-13.95)	147 (132-158)	43.39 (39.08-46.80)	4.15 (2.69 to 5.63)	0	301 (263-339)	247.42 (216.25-278.87)	1734 (1557-1921)	512.62 (460.19-567.85)	2.40 (2.29 to 2.50)	0
Mexico	578 (553-595)	8.31 (7.96-8.56)	3629 (3202-4038)	16.84 (14.86-18.74)	2.35 (1.92 to 2.78)	0	15124 (12598-18048)	217.52 (181.20-259.58)	52283 (44541-60618)	242.61 (206.68-281.28)	0.35 (0.31 to 0.39)	0
Micronesia (Federated States of)	0 (0-1)	6.04 (3.35-10.41)	1 (0-1)	4.36 (2.32-8.20)	-1.05 (-1.13 to -0.98)	0	9 (8-10)	111.74 (97.26-127.74)	16 (14-18)	120.86 (106.54-136.46)	0.24 (0.21 to 0.28)	0
Monaco	2 (1-2)	15.58 (10.58-21.54)	3 (2-4)	17.93 (12.20-25.84)	0.45 (0.41 to 0.49)	0	24 (21-27)	215.84 (189.70-246.75)	37 (34-41)	238.59 (214.53-264.83)	0.33 (0.30 to 0.35)	0

Mongolia	18 (10-27)	10.30 (5.84-15.87)	21 (12-35)	5.36 (3.12-8.87)	-2.10 (-2.43 to -1.76)	0	165 (143-188)	95.41 (82.69-108.67)	421 (370-480)	106.75 (93.79-121.60)	0.36 (0.33 to 0.39)	0
Montenegro	1 (0-1)	0.50 (0.28-0.86)	1 (1-2)	0.53 (0.29-0.89)	0.25 (-0.16 to 0.67)	0.235	64 (54-76)	59.07 (49.23-69.98)	96 (82-111)	54.85 (46.78-63.79)	-0.23 (-0.27 to -0.20)	0
Morocco	4 (0-13)	0.17 (0.00-0.56)	54 (5-169)	0.90 (0.08-2.81)	5.68 (4.95 to 6.42)	0	1856 (1565-2219)	79.14 (66.75-94.62)	6884 (5936-8012)	114.58 (98.79-133.36)	1.20 (1.17 to 1.23)	0
Mozambique	28 (9-56)	2.84 (0.94-5.73)	56 (21-114)	3.13 (1.15-6.35)	0.34 (0.27 to 0.42)	0	371 (307-446)	37.94 (31.41-45.65)	782 (664-920)	43.68 (37.10-51.43)	0.46 (0.43 to 0.49)	0
Myanmar	61 (8-217)	1.56 (0.20-5.54)	166 (30-517)	1.97 (0.35-6.12)	0.77 (0.73 to 0.80)	0	2516 (2089-3021)	64.27 (53.37-77.17)	9989 (8595-11533)	118.32 (101.82-136.62)	2.00 (1.96 to 2.04)	0
Namibia	9 (2-19)	8.34 (2.04-17.18)	18 (4-36)	7.90 (1.98-15.93)	-0.19 (-0.28 to -0.10)	0	137 (116-161)	126.08 (106.95-148.04)	316 (278-356)	139.77 (122.95-157.71)	0.32 (0.28 to 0.37)	0
Nauru	0 (0-0)	5.20 (2.57-9.51)	0 (0-0)	5.45 (2.49-10.99)	0.14 (0.10 to 0.18)	0	1 (1-1)	149.40 (131.92-170.05)	1 (1-2)	149.81 (132.23-168.03)	0.00 (-0.04 to 0.05)	0.922271
Nepal	312 (163-520)	20.24 (10.58-33.78)	989 (635-1420)	24.98 (16.05-35.87)	0.69 (0.60 to 0.78)	0	3147 (2679-3665)	204.41 (174.01-238.09)	13503 (11722-15231)	341.18 (296.16-384.82)	1.68 (1.58 to 1.78)	0

Netherlands	100 (91-109)	3.03 (2.75-3.28)	953 (809-1064)	16.29 (13.83-18.19)	5.76 (5.13 to 6.40)	0	3907 (3430-4500)	118.00 (103.59-135.90)	14105 (12638-15693)	241.08 (216.01-268.22)	2.34 (2.28 to 2.39)	0
New Zealand	46 (42-50)	7.01 (6.36-7.62)	262 (226-289)	18.38 (15.85-20.29)	3.34 (2.86 to 3.83)	0	1531 (1269-1852)	232.72 (192.85-281.52)	3888 (3342-4461)	272.59 (234.28-312.76)	0.51 (0.45 to 0.56)	0
Nicaragua	8 (6-12)	3.27 (2.41-5.01)	37 (21-50)	4.50 (2.60-6.14)	0.95 (0.54 to 1.36)	0	216 (185-252)	87.09 (74.55-101.37)	1217 (1084-1358)	149.46 (133.16-166.83)	1.79 (1.73 to 1.85)	0
Niger	37 (13-66)	8.36 (2.92-15.03)	81 (29-168)	5.83 (2.12-12.06)	-1.15 (-1.40 to -0.90)	0	205 (172-244)	46.70 (39.24-55.44)	496 (423-582)	35.65 (30.42-41.82)	-0.87 (-0.94 to -0.80)	0
Nigeria	497 (183-805)	6.82 (2.51-11.04)	777 (282-1459)	5.18 (1.88-9.72)	-0.88 (-1.03 to -0.73)	0	4012 (3290-4939)	55.07 (45.16-67.79)	6812 (5721-8114)	45.41 (38.14-54.09)	-0.62 (-0.65 to -0.59)	0
Niue	0 (0-0)	7.16 (4.61-11.93)	0 (0-0)	5.07 (2.83-8.86)	-1.12 (-1.24 to -1.01)	0	1 (0-1)	145.97 (128.57-163.78)	1 (1-1)	154.94 (138.45-173.75)	0.19 (0.14 to 0.24)	0
North Macedonia	3 (2-4)	0.90 (0.64-1.27)	6 (3-12)	1.09 (0.52-2.02)	0.68 (0.50 to 0.85)	0	180 (151-212)	54.75 (45.89-64.44)	322 (275-376)	54.33 (46.41-63.46)	-0.03 (-0.06 to 0.00)	0.088014
Northern Mariana Islands	0 (0-0)	8.15 (5.69-11.32)	1 (0-1)	5.43 (3.22-9.91)	-1.32 (-1.93 to -0.70)	0	5 (4-6)	197.43 (167.92-231.60)	19 (16-21)	193.17 (168.60-219.66)	-0.09 (-0.31 to 0.12)	0.390669

Norway	83 (77-87)	7.66 (7.13-8.06)	237 (209-254)	14.58 (12.90-15.64)	2.17 (1.34 to 3.01)	0	2848 (2375-3413)	262.97 (219.31-315.17)	4673 (3989-5447)	288.12 (245.98-335.90)	0.28 (0.16 to 0.39)	4.00E-06
Oman	3 (1-4)	2.52 (1.47-4.48)	10 (5-16)	3.09 (1.56-5.10)	0.68 (0.13 to 1.24)	0.015	85 (72-101)	85.04 (71.83-100.65)	448 (386-516)	143.51 (123.73-165.29)	1.69 (1.62 to 1.76)	0
Pakistan	1124 (684-1656)	12.32 (7.50-18.16)	2304 (1523-3293)	11.62 (7.68-16.61)	-0.17 (-0.30 to -0.04)	0.01	16361 (13415-19631)	179.41 (147.10-215.26)	24931 (20964-29452)	125.75 (105.74-148.55)	-1.15 (-1.18 to -1.12)	0
Palau	0 (0-0)	5.12 (3.22-8.87)	0 (0-0)	3.81 (2.29-6.75)	-0.98 (-1.11 to -0.84)	0	3 (2-3)	160.31 (141.29-181.20)	7 (6-7)	155.93 (138.14-175.91)	-0.10 (-0.15 to -0.05)	0.00024
Palestine	27 (19-37)	18.61 (12.77-25.76)	67 (42-90)	15.67 (9.74-20.94)	-0.57 (-0.77 to -0.37)	0	460 (399-524)	316.93 (274.56-360.78)	1895 (1689-2110)	440.52 (392.57-490.45)	1.08 (0.98 to 1.17)	0
Panama	13 (11-14)	5.18 (4.65-5.74)	115 (90-139)	15.49 (12.10-18.72)	3.71 (3.09 to 4.33)	0	277 (244-313)	114.44 (100.65-129.17)	1745 (1569-1914)	234.67 (210.98-257.44)	2.35 (2.32 to 2.39)	0
Papua New Guinea	25 (15-44)	8.42 (4.97-14.91)	76 (40-137)	9.23 (4.86-16.74)	0.30 (0.16 to 0.44)	0	417 (357-486)	141.10 (120.60-164.21)	1357 (1197-1522)	165.17 (145.75-185.29)	0.50 (0.44 to 0.56)	0
Paraguay	16 (11-24)	4.36 (3.16-6.68)	65 (40-95)	6.53 (4.01-9.56)	1.33 (1.00 to 1.66)	0	183 (157-212)	50.36 (43.24-58.40)	740 (652-835)	74.75 (65.82-84.30)	1.29 (1.27 to 1.31)	0

Peru	934 (656-1305)	47.36 (33.25-66.21)	4177 (2894-5510)	74.67 (51.74-98.52)	1.55 (0.89 to 2.21)	0	8157 (7285-8990)	413.73 (369.50-455.97)	45990 (42300-49650)	822.22 (756.24-887.65)	2.27 (2.20 to 2.33)	0
Philippines	9 (5-14)	0.18 (0.11-0.29)	26 (16-38)	0.19 (0.11-0.27)	0.26 (0.08 to 0.44)	0.005	1991 (1618-2466)	41.71 (33.88-51.65)	4150 (3387-5041)	29.76 (24.29-36.15)	-1.09 (-1.11 to -1.06)	0
Poland	290 (277-302)	3.77 (3.60-3.92)	682 (620-743)	5.63 (5.12-6.14)	1.34 (0.37 to 2.32)	0.007	9699 (8117-11637)	125.87 (105.33-151.01)	15370 (13443-17736)	126.86 (110.96-146.39)	0.03 (-0.06 to 0.13)	0.482086
Portugal	98 (91-107)	4.03 (3.71-4.37)	701 (592-777)	18.01 (15.21-19.98)	5.05 (4.38 to 5.73)	0	3081 (2647-3564)	126.39 (108.59-146.20)	10205 (9242-11129)	262.30 (237.54-286.04)	2.39 (2.36 to 2.42)	0
Puerto Rico	46 (42-51)	7.65 (6.90-8.39)	211 (174-249)	18.04 (14.91-21.33)	2.95 (1.65 to 4.28)	0	653 (578-737)	107.70 (95.23-121.48)	2313 (2078-2551)	198.16 (178.00-218.49)	2.00 (1.96 to 2.03)	0
Qatar	1 (0-1)	3.71 (2.54-6.04)	4 (2-6)	2.58 (1.38-4.20)	-1.58 (-2.48 to -0.68)	0.001	18 (15-21)	108.06 (90.99-127.07)	181 (156-210)	118.29 (101.68-137.14)	0.30 (0.22 to 0.37)	0
Republic of Korea	426 (253-757)	8.56 (5.08-15.22)	1995 (1071-2758)	11.93 (6.40-16.49)	1.02 (0.75 to 1.30)	0	12894 (11360-14691)	259.09 (228.26-295.20)	91426 (82275-101502)	546.68 (491.96-606.93)	2.45 (2.35 to 2.55)	0

Republic of Moldova	20 (17-22)	2.53 (2.23-2.91)	2 (2-3)	0.21 (0.18-0.24)	-7.52 (-8.49 to -6.53)	0	390 (320-461)	50.49 (41.46-59.71)	487 (409-569)	46.10 (38.76-53.86)	-0.29 (-0.31 to -0.27)	0
Romania	551 (480-639)	11.01 (9.59-12.77)	367 (317-418)	6.11 (5.28-6.95)	-1.91 (-2.74 to -1.08)	0	11366 (9869-12984)	227.14 (197.24-259.47)	10429 (9238-11696)	173.66 (153.84-194.77)	-0.85 (-0.92 to -0.78)	0
Russian Federation	1247 (1109-1361)	3.95 (3.51-4.31)	733 (677-792)	1.72 (1.59-1.86)	-2.93 (-4.00 to -1.83)	0	21031 (17261-25438)	66.63 (54.69-80.60)	18069 (14973-21412)	42.51 (35.23-50.38)	-1.45 (-1.49 to -1.40)	0
Rwanda	24 (8-42)	5.10 (1.66-8.93)	42 (14-97)	3.95 (1.30-9.16)	-0.81 (-1.00 to -0.63)	0	244 (201-294)	52.15 (43.05-62.78)	675 (570-791)	63.85 (53.86-74.80)	0.67 (0.59 to 0.76)	0
Saint Kitts and Nevis	0 (0-0)	4.89 (4.09-5.75)	1 (1-1)	6.46 (5.40-7.55)	0.95 (-0.42 to 2.33)	0.175	6 (5-6)	87.04 (76.28-99.34)	18 (16-20)	136.69 (120.93-152.90)	1.48 (1.43 to 1.52)	0
Saint Lucia	1 (1-1)	6.01 (5.30-6.81)	4 (4-5)	10.21 (8.40-12.28)	1.69 (-0.02 to 3.42)	0.053	12 (10-13)	80.30 (70.37-90.36)	53 (48-58)	124.93 (112.90-137.02)	1.43 (1.39 to 1.48)	0
Saint Vincent and the Grenadines	0 (0-0)	0.95 (0.85-1.05)	1 (1-2)	5.27 (4.57-5.92)	5.51 (3.98 to 7.07)	0	5 (4-6)	42.44 (35.90-50.29)	17 (15-19)	66.93 (58.98-75.28)	1.48 (1.43 to 1.53)	0
Samoa	1 (0-2)	5.86 (3.27-10.50)	1 (1-2)	5.51 (2.99-10.15)	-0.21 (-0.28 to -0.14)	0	18 (15-20)	121.69 (106.66-139.28)	33 (30-37)	134.47 (120.29-149.23)	0.32 (0.29 to 0.35)	0

San Marino	0 (0-0)	3.69 (2.41-5.24)	0 (0-1)	2.84 (1.53-4.89)	-0.98 (-1.86 to -0.10)	0.029	5 (5-6)	92.16 (79.15-107.59)	10 (9-11)	83.12 (72.57-95.87)	-0.33 (-0.37 to -0.28)	0
Sao Tome and Principe	2 (1-4)	20.09 (6.89-37.78)	3 (1-6)	16.43 (6.10-34.53)	-0.64 (-0.73 to -0.54)	0	10 (9-12)	90.03 (77.02-105.81)	20 (18-23)	111.92 (98.15-126.30)	0.71 (0.69 to 0.73)	0
Saudi Arabia	169 (108-303)	19.02 (12.14-34.01)	487 (314-706)	16.18 (10.44-23.46)	-0.52 (-0.64 to -0.38)	0	2174 (1881-2476)	244.02 (211.09-277.88)	12485 (11038-13924)	414.68 (366.62-462.49)	1.73 (1.67 to 1.80)	0
Senegal	48 (17-83)	9.14 (3.19-15.65)	85 (32-173)	6.58 (2.45-13.38)	-1.03 (-1.17 to -0.88)	0	262 (223-311)	49.65 (42.11-58.90)	565 (486-650)	43.79 (37.66-50.38)	-0.40 (-0.44 to -0.36)	0
Serbia	45 (31-64)	2.15 (1.47-3.08)	69 (40-100)	2.44 (1.41-3.58)	0.40 (0.28 to 0.52)	0	1332 (1138-1568)	64.05 (54.73-75.38)	2072 (1833-2343)	73.87 (65.35-83.54)	0.45 (0.34 to 0.56)	0
Seychelles	0 (0-0)	1.00 (0.19-3.00)	0 (0-0)	0.70 (0.12-2.09)	-1.09 (-1.29 to -0.89)	0	6 (5-7)	58.35 (48.77-69.75)	15 (13-18)	72.07 (61.01-84.69)	0.68 (0.65 to 0.71)	0
Sierra Leone	30 (10-55)	9.01 (3.03-16.53)	37 (13-81)	6.04 (2.10-13.43)	-1.28 (-1.40 to -1.16)	0	151 (127-179)	45.65 (38.17-54.04)	220 (187-258)	36.43 (30.89-42.69)	-0.72 (-0.79 to -0.66)	0
Singapore	15 (14-17)	4.21 (3.87-4.56)	79 (68-86)	5.18 (4.47-5.66)	0.78 (0.03 to 1.54)	0.042	597 (515-699)	164.67 (141.99-192.78)	3816 (3413-4278)	251.25 (224.68-281.67)	1.38 (1.33 to 1.43)	0

Slovakia	28 (20-43)	2.72 (1.93-4.17)	49 (26-77)	3.00 (1.60-4.71)	0.28 (0.14 to 0.43)	0	870 (753-1012)	83.91 (72.54-97.57)	1805 (1608-2024)	110.05 (98.05-123.37)	0.89 (0.86 to 0.92)	0
Slovenia	19 (16-21)	4.30 (3.80-4.88)	65 (54-75)	8.91 (7.42-10.26)	2.45 (1.21 to 3.71)	0	343 (297-395)	79.69 (68.99-91.75)	1036 (914-1179)	141.49 (124.88-161.00)	1.89 (1.76 to 2.02)	0
Solomon Islands	1 (0-1)	3.07 (1.86-5.10)	2 (1-3)	3.46 (2.06-6.24)	0.33 (0.17 to 0.50)	0	20 (17-24)	88.93 (75.98-104.42)	50 (44-57)	91.94 (80.88-104.90)	0.10 (0.07 to 0.13)	0
Somalia	17 (5-32)	4.60 (1.35-8.77)	37 (13-70)	3.91 (1.42-7.40)	-0.51 (-0.58 to -0.45)	0	183 (152-222)	50.82 (42.26-61.63)	432 (361-512)	45.76 (38.29-54.27)	-0.33 (-0.35 to -0.31)	0
South Africa	354 (189-551)	10.49 (5.60-16.33)	758 (553-1062)	9.61 (7.02-13.46)	-0.31 (-0.71 to 0.10)	0.136	6946 (5759-8276)	205.77 (170.62-245.17)	14372 (12138-16903)	182.19 (153.87-214.28)	-0.41 (-0.49 to -0.34)	0
South Sudan	24 (8-50)	5.69 (1.86-11.99)	27 (10-59)	4.28 (1.60-9.49)	-0.91 (-1.06 to -0.76)	0	239 (199-284)	57.61 (48.09-68.39)	276 (237-322)	44.40 (38.10-51.76)	-0.84 (-0.88 to -0.80)	0
Spain	940 (863-1012)	9.83 (9.02-10.58)	5291 (4358-5905)	34.19 (28.16-38.16)	4.12 (3.46 to 4.79)	0	23521 (21126-25882)	246.02 (220.97-270.72)	61033 (55484-67146)	394.40 (358.54-433.90)	1.54 (1.47 to 1.61)	0
Sri Lanka	93 (70-133)	5.22 (3.92-7.48)	243 (138-392)	5.04 (2.86-8.15)	-0.07 (-0.54 to 0.41)	0.781	1055 (886-1241)	59.29 (49.79-69.75)	5007 (4362-5731)	103.96 (90.57-119.00)	1.84 (1.81 to 1.87)	0

Sudan	3 (0-12)	0.22 (0.00-0.83)	29 (2-105)	0.94 (0.07-3.38)	4.81 (4.59 to 5.04)	0	1087 (900-1307)	73.11 (60.49-87.88)	3032 (2622-3505)	97.38 (84.20-112.56)	0.93 (0.89 to 0.97)	0
Suriname	2 (1-3)	4.61 (3.29-7.24)	6 (3-9)	5.36 (3.01-8.00)	0.58 (0.06 to 1.10)	0.03	28 (24-32)	63.87 (54.89-73.56)	99 (88-110)	88.67 (78.64-98.77)	1.08 (1.03 to 1.13)	0
Sweden	229 (207-247)	9.64 (8.72-10.39)	690 (601-785)	20.50 (17.85-23.33)	2.51 (1.75 to 3.27)	0	6188 (5119-7441)	260.66 (215.64-313.43)	10518 (8975-12205)	312.43 (266.61-362.55)	0.58 (0.54 to 0.62)	0
Switzerland	122 (109-136)	7.20 (6.43-8.03)	359 (303-405)	12.25 (10.35-13.81)	1.79 (1.22 to 2.35)	0	3028 (2702-3372)	179.27 (159.97-199.62)	6161 (5573-6785)	210.21 (190.16-231.49)	0.52 (0.45 to 0.58)	0
Syrian Arab Republic	27 (17-44)	3.09 (1.90-5.03)	90 (52-146)	3.81 (2.21-6.19)	0.71 (0.52 to 0.89)	0	853 (720-1002)	96.73 (81.61-113.70)	3284 (2859-3777)	138.98 (121.00-159.88)	1.17 (1.14 to 1.21)	0
Taiwan (Province of China)	32 (29-35)	1.16 (1.06-1.26)	370 (324-414)	4.91 (4.30-5.50)	4.66 (3.04 to 6.31)	0	1831 (1542-2179)	66.02 (55.61-78.56)	11396 (10033-12877)	151.35 (133.24-171.01)	2.72 (2.64 to 2.80)	0
Tajikistan	91 (41-168)	19.49 (8.83-35.95)	104 (56-186)	10.13 (5.43-18.13)	-2.16 (-2.64 to -1.68)	0	1175 (1025-1330)	250.92 (218.82-283.89)	2606 (2317-2918)	253.76 (225.53-284.06)	0.04 (-0.03 to 0.11)	0.31481
Thailand	57 (39-86)	0.97 (0.66-1.45)	158 (96-275)	0.82 (0.49-1.42)	-0.54 (-0.87 to -0.22)	0.001	2609 (2188-3152)	43.97 (36.86-53.12)	9618 (8150-11324)	49.48 (41.93-58.26)	0.37 (0.34 to 0.41)	0

Timor-Leste	0 (0-1)	0.75 (0.12-2.18)	1 (0-4)	0.92 (0.14-2.64)	0.72 (0.56 to 0.88)	0	21 (17-25)	47.62 (39.08-57.76)	96 (81-111)	67.27 (57.27-78.12)	1.11 (0.98 to 1.23)	0
Togo	16 (6-30)	8.37 (3.08-15.17)	38 (14-82)	6.10 (2.19-13.05)	-1.01 (-1.17 to -0.85)	0	93 (78-110)	47.57 (40.01-56.37)	237 (202-279)	37.98 (32.27-44.73)	-0.73 (-0.77 to -0.69)	0
Tokelau	0 (0-0)	5.78 (2.82-10.24)	0 (0-0)	5.72 (3.16-9.99)	-0.03 (-0.24 to 0.19)	0.803	0 (0-0)	119.14 (103.89-136.32)	0 (0-0)	134.84 (119.98-149.95)	0.41 (0.30 to 0.51)	0
Tonga	0 (0-1)	3.81 (2.33-6.54)	1 (0-1)	4.29 (2.44-7.91)	0.40 (0.25 to 0.54)	0	8 (7-10)	88.78 (77.38-102.81)	13 (12-15)	98.93 (87.83-111.09)	0.34 (0.30 to 0.39)	0
Trinidad and Tobago	10 (9-11)	7.39 (6.67-8.17)	40 (30-49)	11.34 (8.72-14.21)	1.39 (0.92 to 1.85)	0	135 (119-152)	98.29 (86.96-110.37)	449 (404-499)	129.04 (115.88-143.37)	0.90 (0.84 to 0.95)	0
Tunisia	1 (0-4)	0.14 (0.00-0.48)	19 (1-75)	0.83 (0.06-3.22)	5.85 (5.61 to 6.08)	0	778 (657-932)	89.37 (75.45-107.08)	2932 (2528-3392)	125.46 (108.15-145.12)	1.10 (1.09 to 1.12)	0
Türkiye	16 (13-18)	4.86 (4.02-5.50)	11 (8-14)	1.55 (1.18-2.01)	0.95 (0.65 to 1.25)	0	271 (235-312)	83.45 (72.25-95.94)	360 (303-425)	50.82 (42.80-60.12)	-1.60 (-1.65 to -1.55)	0
Turkmenistan	0 (0-0)	5.43 (2.80-9.93)	0 (0-0)	4.87 (2.37-9.11)	-3.52 (-4.19 to -2.85)	0	1 (1-2)	115.57 (100.93-132.52)	2 (2-3)	136.25 (120.90-152.86)	0.53 (0.49 to 0.56)	0

Tuvalu	240 （137-404)	4.02 （2.29-6.77)	873 （572-1291)	5.28 （3.46-7.81)	-0.35 （-0.41 to -0.30)	0	5679 （4898-6611)	95.05 （81.99-110.66)	26548 （23350-29775)	160.69 （141.33-180.22)	1.72 （1.66 to 1.79)	0
Uganda	53 （17-113)	5.10 （1.61-10.83)	104 （34-257)	4.38 （1.42-10.82)	-0.49 （-0.58 to -0.41)	0	581 （488-691)	55.54 （46.66-66.08)	1514 （1289-1754)	63.84 （54.36-73.94)	0.46 （0.41 to 0.52)	0
Ukraine	687 （609-759)	5.51 （4.88-6.09)	173 （129-224)	1.27 （0.95-1.65)	-4.67 （-5.15 to -4.20)	0	22338 （18606-26666)	179.18 （149.24-213.90)	6347 （5298-7488)	46.75 （39.02-55.15)	-4.26 （-4.37 to -4.15)	0
United Arab Emirates	12 （7-20)	21.54 （12.58-34.82)	69 （48-99)	9.58 （6.71-13.73)	-2.65 （-4.42 to -0.86)	0.004	70 （59-84)	125.17 （105.37-149.14)	998 （850-1170)	138.97 （118.42-162.87)	0.34 （0.30 to 0.37)	0
United Kingdom	1667 （1579-1720)	11.22 （10.63-11.58)	8175 （7276-8621)	38.87 （34.60-41.00)	4.15 （3.50 to 4.80)	0	49801 （42062-58904)	335.29 （283.19-396.58)	96016 （82706-109671)	456.56 （393.27-521.49)	1.00 （0.98 to 1.03)	0
United Republic of Tanzania	61 （21-120)	3.39 （1.18-6.66)	139 （47-317)	3.38 （1.14-7.69)	-0.00 （-0.05 to 0.05)	0.975	801 （664-958)	44.41 （36.80-53.12)	2213 （1906-2556)	53.70 （46.24-62.00)	0.62 （0.58 to 0.66)	0
United States of America	6543 （5966-6868)	12.47 （11.37-13.09)	25525 （22191-27142)	25.46 （22.14-27.07)	2.33 （1.94 to 2.73)	0	263278 （221294-310682)	501.84 （421.82-592.20)	587825 （504169-678530)	586.37 （502.92-676.85)	0.55 （0.45 to 0.64)	0

United States Virgin Islands	1 (1-1)	7.15 (5.26-10.65)	2 (1-3)	6.49 (4.29-9.92)	-0.37 (-0.74 to -0.00)	0.048	16 (14-18)	113.61 (99.66-130.28)	62 (56-68)	192.10 (172.99-212.19)	1.70 (1.65 to 1.74)	0
Uruguay	50 (45-54)	7.35 (6.62-8.08)	178 (157-196)	19.72 (17.37-21.68)	3.16 (2.65 to 3.67)	0	1165 (1002-1334)	172.71 (148.51-197.68)	2613 (2373-2851)	289.01 (262.44-315.23)	1.68 (1.65 to 1.71)	0
Uzbekistan	223 (162-314)	11.41 (8.26-16.04)	97 (82-116)	2.09 (1.76-2.50)	-5.16 (-6.11 to -4.20)	0	3816 (3353-4365)	195.13 (171.42-223.18)	6264 (5597-6951)	134.74 (120.40-149.54)	-1.20 (-1.29 to -1.10)	0
Vanuatu	0 (0-1)	4.94 (2.58-8.45)	1 (1-3)	4.88 (2.42-9.12)	-0.03 (-0.29 to 0.23)	0.813	11 (9-12)	105.62 (91.48-123.77)	29 (26-33)	102.40 (91.07-116.13)	-0.11 (-0.14 to -0.08)	0
Venezuela (Bolivarian Republic of)	62 (56-69)	3.99 (3.59-4.40)	365 (282-469)	6.97 (5.38-8.97)	1.89 (1.12 to 2.68)	0	1601 (1397-1834)	102.24 (89.24-117.15)	6632 (5980-7347)	126.76 (114.29-140.41)	0.69 (0.64 to 0.73)	0
Viet Nam	63 (11-187)	0.91 (0.16-2.69)	175 (31-494)	1.00 (0.18-2.83)	0.31 (0.25 to 0.37)	0	3525 (2935-4263)	50.56 (42.09-61.14)	15783 (13368-18454)	90.35 (76.53-105.64)	1.90 (1.85 to 1.95)	0
Yemen	1 (0-5)	0.18 (0.00-0.65)	19 (2-61)	0.86 (0.08-2.70)	5.10 (4.65 to 5.55)	0	571 (478-685)	71.20 (59.54-85.39)	1925 (1651-2228)	85.60 (73.41-99.09)	0.60 (0.57 to 0.63)	0
Zambia	18 (6-33)	3.88 (1.38-7.21)	47 (17-94)	4.33 (1.57-8.74)	0.34 (0.25 to 0.44)	0	227 (192-269)	49.35 (41.70-58.40)	656 (560-765)	60.74 (51.81-70.86)	0.68 (0.64 to 0.72)	0

Zimbabwe	15 （3-28)	2.22 （0.42-4.26)	25 （5-52)	2.27 （0.47-4.68)	0.17 （-0.16 to 0.51)	0.317	513 （432-606)	77.38 （65.18-91.49)	731 （625-855)	66.04 （56.47-77.21)	-0.52 （-0.57 to -0.47)	0
