# Supplementary material for: Global burden, trends and projections analysis of interstitial lung disease and pulmonary sarcoidosis in elderly adults (aged 55+ Years) based on GBD 2021
Source: PLoS One. 2026 Apr 20;21(4):e0347482. doi: 10.1371/journal.pone.0347482 (PMC13095001; doi:10.1371/journal.pone.0347482)
Supplement: S3 Table — Abbreviations: ILD&PS, Interstitial lung disease and pulmonary sarcoidosis; AAPC, average annual per centage change; UI, uncertainty interval. (PDF) [file pone.0347482.s003.pdf]

	Incidence						DALYs（Disability-Adjusted Life Years)					
	Cases（n),1990	Incidence（per 100 000 population), 1990	Cases（n),2021	Incidence（per 100 000 population), 2021	AAPC, 1990–2021	P value	Cases（n),1990	DALYs（per 100 000 population), 1990	Cases（n),2021	DALYs（per 100 000 population), 2021	AAPC, 1990–2021	P value
Afghanistan	68（55-83)	5.61（4.49-6.79)	78（65-92)	6.36（5.25-7.52)	0.41（0.36 to 0.45)	0	386（92-1103)	31.43（7.46-89.74)	145（50-341)	11.90（4.13-28.05)	3.23（2.99 to 3.47)	0
Albania	29（25-35)	8.45（7.08-10.14)	56（47-66)	7.13（6.00-8.36)	-0.54（-0.61 to -0.48)	0	704（402-1146)	89.59（51.16-145.76)	408（271-603)	117.57（78.22-173.77)	-0.92（-1.13 to -0.71)	0
Algeria	141（110-172)	6.72（5.26-8.20)	478（391-570)	7.87（6.44-9.38)	0.51（0.49 to 0.54)	0	1617（570-4094)	26.62（9.39-67.42)	248（120-440)	11.82（5.73-20.98)	2.67（2.53 to 2.81)	0
American Samoa	0（0-0)	5.36（4.36-6.43)	0（0-0)	4.53（3.82-5.29)	-0.54（-0.57 to -0.52)	0	5（4-9)	62.53（43.63-103.40)	3（2-4)	87.76（55.10-120.66)	-1.06（-1.30 to -0.81)	0
Andorra	2（2-2)	20.73（17.66-24.44)	7（6-8)	25.31（22.11-28.77)	0.66（0.59 to 0.72)	0	56（22-102)	212.08（84.91-384.62)	23（14-36)	228.30（137.24-364.35)	-0.28（-0.74 to 0.19)	0.244
Angola	34（27-41)	5.51（4.39-6.63)	111（92-132)	5.74（4.74-6.81)	0.14（0.10 to 0.18)	0	1813（745-3344)	93.75（38.52-172.93)	686（260-1332)	110.18（41.77-213.78)	-0.52（-0.79 to -0.24)	0

Antigua and Barbuda	0 (0-0)	3.97 (3.39-4.54)	1 (1-1)	6.10 (5.32-6.93)	1.39 (1.32 to 1.45)	0	19 (17-20)	99.05 (91.58-107.74)	4 (4-5)	49.78 (44.55-55.43)	2.17 (1.45 to 2.90)	0
Argentina	1198 (1057-1365)	21.53 (19.00-24.52)	3298 (3026-3590)	35.33 (32.42-38.47)	1.60 (1.57 to 1.64)	0	34571 (31396-37873)	370.41 (336.39-405.79)	13548 (12294-14905)	243.48 (220.93-267.86)	1.55 (0.74 to 2.37)	0
Armenia	68 (57-78)	13.89 (11.69-16.10)	135 (121-152)	17.18 (15.34-19.28)	0.71 (0.57 to 0.85)	0	984 (870-1119)	125.06 (110.65-142.30)	932 (846-1012)	191.42 (173.79-207.96)	-1.41 (-3.05 to 0.26)	0.097
Australia	488 (434-549)	14.86 (13.23-16.72)	2615 (2343-2883)	35.30 (31.63-38.92)	2.83 (2.76 to 2.90)	0	25793 (22296-28301)	348.18 (300.98-382.04)	4016 (3653-4373)	122.37 (111.31-133.26)	3.31 (2.51 to 4.12)	0
Austria	146 (123-170)	7.50 (6.32-8.76)	293 (262-329)	9.91 (8.86-11.14)	0.90 (0.86 to 0.94)	0	4219 (3772-4669)	142.77 (127.66-157.98)	1439 (1295-1580)	73.97 (66.55-81.19)	2.33 (1.57 to 3.09)	0
Azerbaijan	84 (70-98)	9.63 (8.07-11.25)	178 (150-209)	9.30 (7.81-10.90)	-0.11 (-0.18 to -0.05)	0.001	1378 (702-2332)	71.90 (36.66-121.73)	975 (468-1771)	111.73 (53.63-203.05)	-1.45 (-1.64 to -1.27)	0
Bahamas	2 (2-2)	8.44 (7.28-9.64)	8 (7-9)	10.98 (9.78-12.29)	0.86 (0.81 to 0.91)	0	166 (136-203)	230.96 (189.68-282.18)	29 (26-33)	119.07 (106.23-133.54)	1.98 (1.07 to 2.91)	0
Bahrain	3 (3-4)	11.28 (9.27-13.33)	30 (26-35)	18.68 (15.96-21.81)	1.64 (1.56 to 1.71)	0	324 (200-461)	199.52 (123.01-283.95)	53 (37-89)	187.32 (131.55-318.82)	0.12 (-0.09 to 0.32)	0.261

Bangladesh	1461 （1243-1702)	19.28 （16.41-22.48)	4693 （4071-5429)	20.08 （17.42-23.23)	0.13 （0.09 to 0.17)	0	65963 （44146-99449)	282.30 （188.93-425.61)	25324 （14497-39243)	334.32 （191.39-518.08)	-0.52 （-0.68 to -0.36)	0
Barbados	3 （3-4)	6.97 （6.10-7.79)	9 （8-10)	10.10 （9.07-11.19)	1.21 （1.17 to 1.24)	0	162 （130-194)	177.41 （143.15-213.33)	46 （41-50)	97.12 （87.35-106.81)	2.19 （1.76 to 2.62)	0
Belarus	175 （133-227)	7.56 （5.76-9.83)	59 （45-75)	2.06 （1.58-2.62)	-4.13 （-4.22 to -4.03)	0	872 （720-1039)	30.31 （25.01-36.13)	3895 （3315-4428)	168.26 （143.20-191.29)	-5.31 （-5.65 to -4.97)	0
Belgium	273 （243-306)	10.40 （9.27-11.65)	668 （599-738)	17.66 （15.84-19.53)	1.72 （1.69 to 1.75)	0	9244 （8254-10088)	244.49 （218.31-266.82)	3026 （2772-3262)	115.43 （105.72-124.43)	2.41 （1.67 to 3.15)	0
Belize	2 （2-3)	15.70 （13.58-17.95)	13 （12-14)	25.71 （23.14-28.60)	1.60 （1.50 to 1.70)	0	205 （176-243)	409.21 （351.89-484.54)	32 （27-39)	208.22 （173.97-251.86)	2.12 （0.43 to 3.84)	0.014
Benin	14 （12-17)	4.46 （3.69-5.27)	24 （20-28)	2.91 （2.42-3.41)	-1.38 （-1.43 to -1.33)	0	1035 （395-2216)	125.20 （47.70-267.90)	631 （241-1089)	197.33 （75.26-340.36)	-1.50 （-1.74 to -1.26)	0
Bermuda	2 （2-2)	19.31 （17.28-21.40)	7 （6-7)	28.91 （26.29-31.84)	1.31 （1.29 to 1.33)	0	87 （75-104)	375.32 （320.93-446.78)	28 （24-33)	264.97 （224.42-308.36)	1.08 （0.12 to 2.05)	0.027
Bhutan	7 （6-9)	18.34 （15.34-21.46)	29 （25-33)	29.01 （25.47-32.91)	1.49 （1.46 to 1.52)	0	326 （216-498)	327.55 （216.57-500.06)	110 （68-166)	277.84 （170.17-417.83)	0.55 （0.45 to 0.65)	0

Bolivia (Plurinational State of)	275 (244-311)	52.52 (46.53-59.38)	1284 (1168-1419)	83.08 (75.60-91.79)	1.50 (1.47 to 1.53)	0	12675 (8118-18319)	820.15 (525.26-1185.37)	3641 (1801-6957)	694.44 (343.39-1326.85)	0.55 (0.47 to 0.63)	0
Bosnia and Herzegovina	42 (33-52)	5.68 (4.51-7.05)	50 (42-59)	4.57 (3.86-5.42)	-0.69 (-0.75 to -0.64)	0	690 (429-1090)	63.00 (39.20-99.51)	594 (373-899)	80.93 (50.83-122.53)	-0.79 (-1.10 to -0.48)	0
Botswana	12 (10-14)	12.87 (10.61-15.30)	31 (26-35)	12.62 (10.90-14.57)	-0.06 (-0.12 to 0.00)	0.067	313 (105-632)	129.32 (43.11-260.57)	174 (51-341)	186.66 (54.72-366.80)	-1.23 (-1.77 to -0.68)	0
Brazil	1090 (857-1326)	7.37 (5.80-8.97)	4958 (4184-5707)	11.45 (9.66-13.18)	1.42 (1.37 to 1.48)	0	73306 (66963-77935)	169.27 (154.62-179.96)	13354 (12459-14314)	90.36 (84.31-96.86)	2.08 (1.68 to 2.48)	0
Brunei Darussalam	9 (8-10)	56.85 (48.53-65.39)	28 (24-31)	46.25 (40.69-52.46)	-0.67 (-0.73 to -0.61)	0	151 (112-198)	252.71 (186.44-331.12)	57 (40-78)	365.47 (255.62-501.09)	-1.21 (-1.52 to -0.91)	0
Bulgaria	78 (63-96)	3.44 (2.79-4.24)	56 (47-66)	2.35 (1.96-2.77)	-1.23 (-1.28 to -1.19)	0	1282 (1093-1504)	53.93 (45.98-63.28)	1066 (907-1229)	47.10 (40.05-54.29)	0.61 (0.02 to 1.21)	0.043
Burkina Faso	23 (19-28)	3.17 (2.57-3.82)	32 (26-38)	2.11 (1.72-2.51)	-1.31 (-1.37 to -1.25)	0	1313 (522-2568)	87.33 (34.72-170.83)	831 (297-1445)	114.95 (41.01-199.77)	-0.87 (-0.98 to -0.76)	0
Burundi	22 (18-27)	5.92 (4.78-7.08)	46 (38-54)	5.71 (4.73-6.73)	-0.12 (-0.15 to -0.09)	0	754 (276-1628)	94.22 (34.46-203.34)	455 (156-801)	120.88 (41.52-212.80)	-0.80 (-0.95 to -0.65)	0

Cabo Verde	1（1-1)	1.91（1.54-2.26)	2（1-2)	2.07（1.64-2.52)	0.27（0.22 to 0.32)	0	78（32-146)	102.02（41.99-190.08)	97（27-205)	246.15（67.71-518.84)	-2.95（-3.67 to -2.22)	0
Cambodia	26（21-31)	3.52（2.84-4.22)	92（75-107)	4.22（3.45-4.94)	0.58（0.57 to 0.59)	0	418（146-1067)	19.24（6.72-49.06)	126（37-352)	16.98（4.93-47.47)	0.39（0.34 to 0.43)	0
Cameroon	34（28-41)	4.57（3.74-5.53)	59（49-70)	2.92（2.40-3.43)	-1.45（-1.49 to -1.40)	0	2911（1098-5496)	143.04（53.94-270.00)	1560（630-2649)	210.66（85.11-357.73)	-1.25（-1.35 to -1.15)	0
Canada	1272（1141-1425)	23.31（20.90-26.11)	5809（5195-6471)	47.34（42.34-52.74)	2.32（2.28 to 2.35)	0	52261（46770-57102)	425.88（381.14-465.33)	12915（11792-14242)	236.57（216.01-260.89)	1.98（1.22 to 2.74)	0
Central African Republic	11（8-13)	5.70（4.46-6.97)	19（15-23)	5.26（4.26-6.48)	-0.26（-0.31 to -0.22)	0	446（172-923)	124.56（48.04-258.10)	256（87-496)	135.69（46.23-262.69)	-0.26（-0.40 to -0.12)	0
Chad	19（16-23)	4.20（3.45-4.95)	28（23-33)	3.02（2.53-3.55)	-1.07（-1.10 to -1.04)	0	1353（530-2559)	145.68（57.05-275.64)	849（312-1545)	183.12（67.43-333.41)	-0.72（-0.78 to -0.66)	0
Chile	612（557-672)	36.41（33.12-39.94)	3191（2923-3509)	71.26（65.28-78.36)	2.21（2.15 to 2.26)	0	36706（33570-39315)	819.76（749.71-878.02)	6571（6101-6990)	390.72（362.78-415.64)	2.45（1.88 to 3.02)	0
China	8078（6035-10324)	5.63（4.20-7.19)	33281（27064-39626)	8.78（7.14-10.46)	1.43（1.23 to 1.62)	0	179166（124959-230460)	47.28（32.97-60.81)	64212（49219-96643)	44.74（34.29-67.34)	0.16（0.02 to 0.31)	0.028

Colombia	157 （131-184)	5.47 （4.55-6.37)	957 （849-1071)	10.00 （8.88-11.20)	1.98 （1.95 to 2.01)	0	14169 （11945-16707)	148.16 （124.91-174.70)	1418 （1289-1555)	49.26 （44.79-54.01)	3.70 （2.92 to 4.49)	0
Comoros	2 （2-2)	5.94 （4.71-7.12)	4 （4-5)	5.56 （4.65-6.48)	-0.21 （-0.24 to -0.18)	0	70 （28-152)	86.18 （34.71-188.51)	35 （13-66)	107.49 （39.28-204.88)	-0.72 （-0.86 to -0.57)	0
Congo	11 （9-13)	6.08 （4.83-7.39)	27 （23-33)	6.31 （5.20-7.50)	0.12 （0.08 to 0.15)	0	524 （221-983)	120.56 （50.84-226.24)	260 （90-551)	146.18 （50.70-309.61)	-0.63 （-0.72 to -0.54)	0
Cook Islands	0 （0-0)	7.87 （6.60-9.35)	0 （0-0)	7.18 （6.10-8.39)	-0.30 （-0.36 to -0.24)	0	4 （2-7)	75.93 （43.43-140.78)	2 （1-3)	94.90 （64.58-152.77)	-0.75 （-0.94 to -0.57)	0
Costa Rica	57 （50-64)	20.09 （17.73-22.53)	296 （270-327)	30.87 （28.18-34.17)	1.38 （1.34 to 1.42)	0	3098 （2739-3477)	323.60 （286.11-363.19)	568 （513-621)	199.60 （180.24-218.31)	1.73 （0.75 to 2.73)	0.001
Côte d'Ivoire	29 （23-35)	2.25 （1.72-2.91)	55 （45-65)	2.27 （1.82-2.77)	-1.24 （-1.27 to -1.20)	0	625 （539-715)	41.88 （36.11-47.97)	345 （306-391)	31.02 （27.51-35.09)	-1.20 （-1.35 to -1.05)	0
Croatia	25 （19-32)	2.39 （1.96-2.87)	34 （27-41)	2.59 （2.18-3.01)	0.04 （-0.00 to 0.08)	0.073	1212 （1045-1371)	35.46 （30.60-40.13)	307 （270-347)	18.14 （15.95-20.46)	0.96 （0.20 to 1.73)	0.014
Cuba	41 （33-49)	31.68 （26.77-37.15)	89 （74-103)	34.14 （29.94-38.66)	0.26 （0.20 to 0.31)	0	1509 （1086-2002)	431.81 （310.71-572.77)	829 （550-1262)	593.26 （393.67-903.59)	2.28 （1.60 to 2.95)	0

Cyprus	44 （37-52)	3.31 （2.65-4.06)	119 （105-135)	8.18 （7.04-9.43)	0.24 （0.19 to 0.29)	0	6505 （5650-7417)	186.22 （161.74-212.32)	1731 （1480-2021)	73.10 （62.50-85.34)	-1.17 （-1.60 to -0.74)	0
Czechia	78 （63-96)	4.51 （3.60-5.47)	286 （246-330)	3.07 （2.53-3.64)	2.98 （2.90 to 3.05)	0	2325 （850-4504)	129.66 （47.39-251.15)	1204 （445-2133)	187.91 （69.45-332.86)	3.14 （2.02 to 4.27)	0
Democratic People's Republic of Korea	138 （110-169)	5.18 （4.13-6.34)	297 （247-350)	5.26 （4.38-6.20)	0.05 （0.01 to 0.09)	0.007	2662 （1533-4856)	47.24 （27.21-86.16)	1093 （607-2020)	41.06 （22.79-75.90)	0.45 （0.39 to 0.52)	0
Democratic Republic of the Congo	154 （120-188)	5.82 （4.54-7.12)	374 （302-452)	6.20 （4.99-7.48)	0.20 （0.19 to 0.21)	0	8720 （3287-23736)	144.38 （54.43-392.99)	3384 （1270-7599)	128.13 （48.10-287.77)	0.38 （0.26 to 0.50)	0
Denmark	176 （155-199)	13.50 （11.86-15.27)	363 （326-402)	18.85 （16.93-20.89)	1.09 （1.02 to 1.15)	0	5371 （4817-5976)	279.01 （250.19-310.38)	1759 （1591-1936)	134.84 （121.93-148.37)	2.39 （1.66 to 3.13)	0
Djibouti	1 （1-1)	5.43 （4.33-6.54)	6 （5-7)	5.58 （4.60-6.64)	0.09 （0.07 to 0.11)	0	73 （29-149)	70.32 （28.14-143.33)	18 （7-33)	84.33 （32.30-153.04)	-0.57 （-0.73 to -0.42)	0
Dominica	1 （0-1)	5.87 （5.03-6.75)	1 （1-1)	8.71 （7.76-9.78)	1.28 （1.27 to 1.29)	0	16 （10-22)	106.46 （68.89-146.74)	8 （6-12)	80.03 （58.55-118.92)	0.94 （0.81 to 1.07)	0
Dominican Republic	17 （14-20)	2.74 （2.28-3.25)	75 （65-86)	4.51 （3.87-5.15)	1.63 （1.59 to 1.67)	0	778 （456-1237)	46.59 （27.27-74.04)	184 （111-353)	30.04 （18.18-57.68)	1.42 （1.02 to 1.81)	0

Ecuador	257（227-290)	29.88（26.46-33.74)	1639（1501-1779)	59.22（54.23-64.28)	2.26（2.16 to 2.35)	0	18526（15224-22404)	669.42（550.10-809.53)	2369（2111-2664)	275.54（245.47-309.85)	2.86（2.00 to 3.72)	0
Egypt	268（210-329)	5.99（4.70-7.36)	862（690-1058)	7.79（6.24-9.56)	0.85（0.80 to 0.91)	0	5215（3856-6842)	47.13（34.84-61.83)	3010（2211-4164)	67.32（49.44-93.13)	-1.16（-1.48 to -0.84)	0
El Salvador	55（48-62)	11.23（9.83-12.65)	231（207-256)	22.62（20.25-24.99)	2.30（2.22 to 2.38)	0	1947（1291-2564)	190.37（126.25-250.72)	707（527-1089)	144.57（107.82-222.77)	0.88（0.66 to 1.11)	0
Equatorial Guinea	2（1-2)	5.76（4.63-6.96)	6（5-7)	7.15（5.88-8.45)	0.71（0.67 to 0.74)	0	85（33-181)	107.05（42.20-228.75)	42（15-81)	131.70（46.33-252.91)	-0.64（-0.97 to -0.31)	0
Eritrea	8（6-10)	4.53（3.57-5.59)	21（17-24)	4.70（3.90-5.54)	0.12（0.10 to 0.14)	0	401（167-700)	91.05（37.86-158.96)	182（55-335)	102.92（31.16-189.60)	-0.43（-0.48 to -0.37)	0
Estonia	8（6-10)	2.26（1.73-2.90)	14（12-17)	3.23（2.64-3.85)	1.17（1.11 to 1.23)	0	183（156-215)	41.73（35.59-49.26)	974（856-1114)	271.75（238.82-310.77)	-5.69（-7.29 to -4.06)	0
Eswatini	6（5-7)	13.10（10.95-15.47)	9（8-11)	10.33（8.90-11.92)	-0.77（-0.81 to -0.72)	0	152（49-291)	167.36（53.86-321.71)	88（26-177)	195.03（58.59-391.29)	-0.50（-0.63 to -0.37)	0
Ethiopia	156（120-190)	4.84（3.73-5.92)	359（295-428)	5.25（4.31-6.25)	0.26（0.21 to 0.31)	0	5113（1743-10660)	74.70（25.45-155.72)	2951（893-5254)	91.85（27.78-163.53)	-0.66（-0.76 to -0.56)	0

Fiji	2 （2-3)	3.65 （2.95-4.45)	5 （4-5)	3.35 （2.74-4.02)	-0.28 （-0.31 to -0.25)	0	73 （46-105)	53.09 （33.46-76.53)	29 （21-39)	50.97 （37.81-68.45)	0.10 （-0.04 to 0.24)	0.172
Finland	167 （146-191)	14.07 （12.29-16.08)	540 （479-601)	26.85 （23.80-29.88)	2.12 （2.00 to 2.24)	0	7117 （6249-7819)	353.61 （310.49-388.48)	1799 （1586-2046)	151.49 （133.61-172.29)	2.80 （2.22 to 3.40)	0
France	1610 （1441-1820)	11.57 （10.35-13.08)	4186 （3787-4577)	18.93 （17.13-20.70)	1.61 （1.58 to 1.64)	0	48836 （42525-54656)	220.88 （192.33-247.20)	15950 （14436-17514)	114.61 （103.73-125.85)	2.17 （1.60 to 2.75)	0
Gabon	7 （5-8)	6.74 （5.49-8.05)	12 （10-15)	6.93 （5.75-8.14)	0.09 （0.06 to 0.13)	0	186 （75-395)	104.24 （42.06-220.88)	136 （51-258)	138.69 （51.69-263.61)	-0.90 （-1.00 to -0.81)	0
Gambia	2 （2-3)	4.42 （3.57-5.41)	5 （4-6)	3.10 （2.61-3.63)	-1.15 （-1.19 to -1.11)	0	242 （97-483)	156.73 （62.57-312.63)	111 （40-202)	200.09 （72.68-364.21)	-0.82 （-1.36 to -0.28)	0.003
Georgia	80 （68-93)	7.20 （6.14-8.41)	98 （89-109)	9.32 （8.44-10.34)	0.86 （0.73 to 0.99)	0	675 （570-784)	64.26 （54.31-74.71)	674 （563-801)	60.66 （50.71-72.09)	0.18 （-2.08 to 2.50)	0.874
Germany	2400 （2093-2728)	11.44 （9.98-13.00)	5327 （4820-5853)	16.91 （15.30-18.57)	1.28 （1.23 to 1.32)	0	72703 （65475-79328)	230.70 （207.77-251.72)	29086 （25835-32719)	138.61 （123.12-155.93)	1.75 （1.51 to 1.99)	0
Ghana	64 （52-77)	6.24 （5.13-7.54)	149 （127-171)	5.45 （4.67-6.27)	-0.43 （-0.47 to -0.40)	0	3575 （1560-5928)	131.07 （57.19-217.38)	1287 （506-2093)	126.02 （49.53-204.95)	0.13 （0.06 to 0.20)	0.001

Greece	150 （125-175)	5.59 （4.69-6.53)	509 （451-571)	13.85 （12.26-15.53)	3.00 （2.90 to 3.10)	0	8501 （7597-9295)	231.29 （206.71-252.90)	1206 （1099-1329)	45.12 （41.11-49.70)	5.42 （3.24 to 7.65)	0
Greenland	2 （2-3)	39.38 （33.67-46.14)	6 （5-7)	41.81 （36.36-48.11)	0.19 （0.11 to 0.26)	0	44 （21-65)	321.92 （155.50-475.42)	23 （13-32)	423.92 （237.60-580.93)	-0.81 （-1.06 to -0.57)	0
Grenada	1 （1-1)	6.16 （5.42-6.93)	2 （1-2)	8.31 （7.41-9.34)	0.97 （0.93 to 1.01)	0	30 （26-34)	149.69 （129.42-169.42)	9 （7-10)	71.46 （61.14-81.42)	2.29 （1.20 to 3.40)	0
Guam	3 （2-3)	21.04 （17.62-24.86)	6 （5-6)	14.95 （12.76-17.16)	-1.09 （-1.13 to -1.05)	0	68 （52-99)	179.46 （138.23-262.23)	45 （33-58)	346.13 （253.45-449.43)	-2.12 （-2.37 to -1.87)	0
Guatemala	79 （70-89)	13.72 （12.10-15.48)	357 （327-390)	19.50 （17.87-21.32)	1.14 （1.08 to 1.20)	0	5442 （4636-6278)	297.23 （253.25-342.92)	1147 （968-1392)	199.45 （168.40-241.96)	1.06 （-0.23 to 2.38)	0.108
Guinea	25 （20-30)	4.47 （3.68-5.36)	28 （24-33)	3.12 （2.62-3.65)	-1.16 （-1.20 to -1.12)	0	1326 （521-2723)	145.14 （57.02-298.11)	1015 （406-1744)	183.36 （73.44-315.08)	-0.75 （-0.87 to -0.63)	0
Guinea-Bissau	3 （2-3)	4.33 （3.45-5.29)	3 （3-4)	2.72 （2.21-3.28)	-1.50 （-1.54 to -1.46)	0	177 （73-311)	154.05 （63.31-270.71)	153 （50-295)	239.40 （78.73-461.83)	-1.41 （-1.51 to -1.31)	0
Guyana	4 （3-5)	6.44 （5.59-7.37)	9 （7-10)	7.55 （6.62-8.59)	0.51 （0.46 to 0.56)	0	169 （130-215)	150.23 （115.30-190.60)	87 （76-100)	142.43 （124.04-162.85)	0.45 （-0.83 to 1.74)	0.493

Haiti	35（29-41)	6.40（5.33-7.61)	98（84-114)	8.27（7.07-9.58)	0.83（0.80 to 0.86)	0	2004（969-3745)	169.08（81.79-316.01)	735（297-1208)	136.20（54.99-223.93)	0.71（0.62 to 0.80)	0
Honduras	40（34-48)	12.07（10.09-14.29)	209（180-241)	19.91（17.09-22.92)	1.63（1.58 to 1.67)	0	3519（1804-5228)	334.63（171.58-497.07)	668（445-1009)	200.39（133.60-302.59)	1.68（1.46 to 1.90)	0
Hungary	148（125-174)	5.74（4.85-6.75)	218（191-248)	6.85（6.02-7.80)	0.58（0.53 to 0.62)	0	4673（4092-5259)	146.95（128.67-165.37)	2577（2390-2820)	100.04（92.79-109.48)	1.41（0.84 to 1.98)	0
Iceland	5（4-6)	10.39（8.98-11.79)	16（14-18)	16.37（14.71-18.14)	1.48（1.46 to 1.51)	0	204（180-227)	209.74（185.24-233.42)	38（34-42)	79.90（71.69-88.37)	3.25（2.54 to 3.96)	0
India	18380（14556-22358)	23.97（18.99-29.16)	55615（45880-66289)	27.66（22.82-32.97)	0.46（0.44 to 0.49)	0	881760（576662-1222666)	438.59（286.83-608.15)	279777（159448-434060)	364.91（207.97-566.15)	0.63（0.16 to 1.10)	0.009
Indonesia	825（640-1019)	5.11（3.96-6.31)	2624（2100-3189)	6.27（5.02-7.61)	0.66（0.65 to 0.68)	0	11369（3925-26628)	27.15（9.37-63.58)	3855（1239-9453)	23.86（7.67-58.51)	0.41（0.35 to 0.48)	0
Iran（Islamic Republic of)	152（111-195)	3.30（2.40-4.23)	559（443-681)	4.30（3.41-5.24)	0.86（0.84 to 0.88)	0	1370（755-1970)	10.54（5.81-15.17)	349（226-482)	7.57（4.89-10.45)	1.06（0.97 to 1.15)	0
Iraq	71（58-84)	5.61（4.56-6.66)	245（202-291)	6.30（5.21-7.48)	0.37（0.32 to 0.41)	0	2965（1949-4113)	76.31（50.16-105.87)	841（564-1239)	66.61（44.72-98.12)	0.43（0.34 to 0.53)	0

Ireland	133 （119-148)	19.55 （17.48-21.72)	561 （499-631)	42.76 （38.06-48.04)	2.57 （2.44 to 2.71)	0	6641 （5852-7411)	506.01 （445.94-564.72)	1640 （1505-1772)	240.87 （221.10-260.36)	2.62 （2.34 to 2.91)	0
Israel	84 （73-97)	10.41 （9.00-11.96)	256 （230-282)	12.81 （11.51-14.12)	0.68 （0.63 to 0.73)	0	3235 （2866-3552)	161.88 （143.42-177.73)	917 （821-1008)	113.08 （101.34-124.40)	1.31 （0.88 to 1.74)	0
Italy	1199 （912-1476)	7.88 （5.99-9.70)	4192 （3526-4907)	18.45 （15.52-21.60)	2.80 （2.57 to 3.02)	0	53864 （47949-58275)	237.11 （211.07-256.53)	5640 （4789-6558)	37.06 （31.47-43.09)	6.59 （5.64 to 7.55)	0
Jamaica	9 （8-11)	3.22 （2.72-3.70)	26 （22-29)	4.88 （4.25-5.54)	1.35 （1.33 to 1.38)	0	534 （409-670)	101.00 （77.32-126.64)	121 （108-134)	41.20 （36.62-45.45)	3.05 （1.33 to 4.79)	0
Japan	11411 （9002-14156)	38.53 （30.40-47.80)	30908 （26084-35939)	59.21 （49.97-68.85)	1.38 （1.31 to 1.45)	0	368672 （321401-403055)	706.26 （615.70-772.12)	100024 （91810-107973)	337.77 （310.03-364.61)	2.38 （1.95 to 2.81)	0
Jordan	41 （34-48)	18.97 （15.98-22.37)	281 （242-324)	22.36 （19.25-25.76)	0.52 （0.46 to 0.58)	0	2461 （1760-3334)	195.71 （140.02-265.20)	469 （310-761)	218.27 （144.04-354.17)	-0.33 （-0.54 to -0.12)	0.002
Kazakhstan	119 （99-140)	5.68 （4.74-6.68)	279 （240-324)	8.80 （7.56-10.21)	1.40 （1.29 to 1.52)	0	2940 （2287-3792)	92.65 （72.08-119.49)	1783 （1554-2020)	85.26 （74.30-96.60)	0.08 （-0.91 to 1.07)	0.878
Kenya	77 （61-94)	5.78 （4.54-7.04)	216 （172-261)	5.77 （4.59-6.98)	-0.01 （-0.04 to 0.01)	0.387	4211 （1309-12808)	112.56 （34.99-342.39)	1232 （390-3410)	92.43 （29.26-255.88)	0.61 （0.46 to 0.75)	0

Kiribati	0 (0-0)	5.67 (4.72-6.73)	1 (1-1)	5.51 (4.59-6.47)	-0.09 (-0.12 to -0.05)	0	13 (7-25)	105.12 (57.66-195.25)	7 (3-12)	114.39 (56.00-200.53)	-0.28 (-0.33 to -0.23)	0
Kuwait	12 (10-14)	13.39 (11.27-15.56)	88 (77-100)	18.96 (16.41-21.53)	1.15 (1.02 to 1.27)	0	830 (692-965)	178.03 (148.45-206.96)	215 (187-241)	235.22 (205.03-263.25)	-1.00 (-2.71 to 0.75)	0.261
Kyrgyzstan	23 (20-28)	4.54 (3.82-5.36)	21 (17-26)	2.52 (2.03-3.09)	-1.89 (-2.02 to -1.76)	0	185 (153-218)	21.86 (18.07-25.81)	454 (372-536)	88.14 (72.22-104.18)	-4.82 (-7.40 to -2.17)	0
Lao People's Democratic Republic	17 (14-21)	4.99 (4.04-5.99)	44 (36-52)	5.71 (4.72-6.71)	0.43 (0.42 to 0.45)	0	197 (62-499)	25.42 (7.97-64.57)	89 (22-262)	26.07 (6.54-76.43)	-0.08 (-0.14 to -0.02)	0.005
Latvia	26 (20-32)	4.08 (3.16-5.17)	17 (14-20)	2.56 (2.11-3.04)	-1.48 (-1.52 to -1.44)	0	268 (227-311)	40.69 (34.46-47.27)	2417 (2154-2720)	386.63 (344.57-435.05)	-6.94 (-8.04 to -5.83)	0
Lebanon	26 (21-32)	6.81 (5.41-8.38)	84 (70-99)	8.59 (7.15-10.07)	0.75 (0.74 to 0.77)	0	1874 (1374-2699)	191.23 (140.25-275.40)	638 (318-1169)	168.31 (83.85-308.26)	0.43 (0.29 to 0.58)	0
Lesotho	18 (15-21)	12.80 (10.56-15.11)	20 (17-23)	10.80 (9.23-12.56)	-0.54 (-0.58 to -0.50)	0	281 (82-560)	155.90 (45.44-310.33)	222 (62-436)	158.48 (44.66-312.09)	-0.06 (-0.26 to 0.14)	0.573
Liberia	9 (7-10)	4.54 (3.66-5.42)	11 (9-13)	3.33 (2.72-3.92)	-1.00 (-1.12 to -0.89)	0	451 (157-968)	136.53 (47.41-293.12)	362 (130-682)	192.15 (68.91-362.01)	-1.12 (-1.31 to -0.93)	0

Libya	24（19-29)	7.64（6.08-9.44)	63（52-76)	7.59（6.23-9.11)	-0.03（-0.05 to -0.01)	0.008	299（76-942)	35.79（9.03-112.69)	42（19-77)	13.54（6.19-24.88)	3.19（2.99 to 3.39)	0
Lithuania	16（12-20)	2.00（1.56-2.51)	17（13-21)	1.76（1.39-2.14)	-0.41（-0.48 to -0.33)	0	221（188-257)	22.87（19.38-26.56)	1150（982-1368)	145.18（123.91-172.66)	-5.68（-7.01 to -4.33)	0
Luxembourg	9（8-10)	9.47（8.32-10.77)	26（23-29)	14.56（13.07-16.22)	1.40（1.38 to 1.43)	0	345（303-385)	194.10（170.65-216.93)	81（73-89)	86.98（78.62-95.24)	2.62（2.16 to 3.08)	0
Madagascar	55（45-65)	6.51（5.31-7.66)	123（101-145)	6.63（5.49-7.84)	0.06（0.02 to 0.10)	0.001	2532（1057-5132)	137.09（57.22-277.88)	1216（488-2243)	143.97（57.76-265.52)	-0.14（-0.24 to -0.05)	0.002
Malawi	28（22-33)	4.41（3.55-5.30)	53（44-62)	4.51（3.73-5.34)	0.07（0.04 to 0.11)	0	1115（430-2231)	95.38（36.75-190.81)	521（191-959)	82.84（30.38-152.44)	0.50（0.35 to 0.66)	0
Malaysia	106（89-122)	7.18（6.06-8.30)	438（375-505)	8.93（7.66-10.30)	0.71（0.66 to 0.75)	0	4499（3251-5955)	91.77（66.32-121.46)	1208（882-1755)	81.87（59.76-118.91)	0.36（0.18 to 0.53)	0
Maldives	5（4-6)	30.11（24.23-36.84)	24（21-28)	44.80（38.34-52.21)	1.29（1.27 to 1.31)	0	173（128-225)	317.62（234.74-413.83)	80（42-137)	521.55（275.12-892.17)	-1.65（-2.09 to -1.20)	0
Mali	31（24-38)	4.57（3.59-5.64)	53（44-63)	3.69（3.02-4.38)	-0.70（-0.73 to -0.67)	0	3363（1175-7083)	232.37（81.18-489.36)	1775（607-3165)	265.49（90.79-473.38)	-0.42（-0.52 to -0.32)	0

Malta	15 (13-17)	20.59 (18.44-23.16)	57 (51-62)	35.85 (32.62-39.48)	1.81 (1.68 to 1.94)	0	794 (700-892)	503.37 (443.72-565.09)	146 (132-158)	201.29 (182.66-218.62)	2.86 (2.47 to 3.24)	0
Marshall Islands	0 (0-0)	9.35 (7.78-11.17)	1 (0-1)	9.02 (7.50-10.70)	-0.12 (-0.16 to -0.09)	0	7 (3-13)	111.48 (56.91-214.79)	3 (2-6)	131.53 (72.48-236.99)	-0.54 (-0.61 to -0.48)	0
Mauritania	7 (6-8)	4.28 (3.42-5.11)	11 (9-13)	3.09 (2.58-3.64)	-1.04 (-1.09 to -0.99)	0	446 (180-848)	124.39 (50.26-236.33)	337 (127-567)	203.10 (76.64-341.27)	-1.58 (-1.81 to -1.34)	0
Mauritius	29 (25-33)	23.62 (20.46-27.43)	158 (142-176)	46.66 (41.92-52.09)	2.23 (2.18 to 2.28)	0	2768 (2546-2979)	818.04 (752.58-880.38)	344 (317-372)	282.88 (260.95-306.05)	3.54 (2.16 to 4.94)	0
Mexico	1223 (975-1485)	17.59 (14.02-21.36)	5574 (4696-6512)	25.86 (21.79-30.22)	1.24 (1.18 to 1.29)	0	71953 (63928-80516)	333.88 (296.64-373.62)	12513 (11853-13179)	179.97 (170.48-189.56)	1.90 (1.75 to 2.04)	0
Micronesia (Federated States of)	1 (1-1)	8.52 (7.19-10.05)	1 (1-1)	8.56 (7.06-10.15)	0.01 (-0.03 to 0.06)	0.560	15 (8-27)	110.46 (63.01-201.87)	11 (6-18)	138.01 (79.58-225.88)	-0.72 (-0.78 to -0.67)	0
Monaco	2 (1-2)	15.56 (13.51-18.08)	3 (2-3)	17.98 (15.93-20.27)	0.47 (0.43 to 0.50)	0	46 (33-63)	295.72 (211.39-401.80)	30 (21-40)	268.63 (191.60-361.49)	0.30 (0.27 to 0.34)	0
Mongolia	20 (17-23)	11.57 (10.00-13.22)	37 (31-42)	9.31 (7.89-10.70)	-0.69 (-0.76 to -0.63)	0	474 (286-739)	120.01 (72.32-187.22)	368 (218-566)	212.14 (126.00-326.71)	-1.79 (-2.00 to -1.58)	0

Montenegro	2 (2-3)	2.22 (1.73-2.79)	4 (3-4)	2.04 (1.61-2.49)	-0.27 (-0.30 to -0.25)	0	28 (19-41)	16.28 (10.97-23.73)	18 (12-26)	16.35 (11.07-24.18)	0.04 (-0.22 to 0.29)	0.768
Morocco	157 (127-190)	6.69 (5.41-8.10)	474 (388-572)	7.90 (6.45-9.51)	0.53 (0.52 to 0.55)	0	1729 (560-3944)	28.78 (9.31-65.65)	259 (121-470)	11.04 (5.15-20.03)	3.13 (3.01 to 3.26)	0
Mozambique	39 (31-47)	3.99 (3.22-4.80)	67 (56-80)	3.75 (3.10-4.49)	-0.20 (-0.25 to -0.16)	0	1311 (535-2644)	73.27 (29.91-147.77)	639 (235-1268)	65.36 (24.07-129.71)	0.38 (0.32 to 0.45)	0
Myanmar	268 (218-327)	6.85 (5.56-8.35)	868 (729-1012)	10.28 (8.64-11.99)	1.32 (1.30 to 1.35)	0	4173 (1297-10686)	49.44 (15.37-126.58)	1551 (371-5041)	39.61 (9.49-128.78)	0.72 (0.64 to 0.80)	0
Namibia	14 (12-17)	13.23 (11.09-15.76)	28 (24-32)	12.27 (10.63-14.10)	-0.25 (-0.28 to -0.22)	0	395 (120-783)	174.70 (53.20-346.42)	204 (60-399)	188.21 (55.25-367.83)	-0.23 (-0.39 to -0.08)	0.003
Nauru	0 (0-0)	9.13 (7.49-10.92)	0 (0-0)	9.35 (7.78-11.07)	0.07 (0.05 to 0.09)	0	1 (1-2)	133.09 (64.41-263.26)	1 (1-2)	135.37 (72.90-244.77)	-0.07 (-0.11 to -0.02)	0.003
Nepal	387 (327-457)	25.15 (21.21-29.70)	1327 (1154-1515)	33.54 (29.16-38.28)	0.96 (0.91 to 1.01)	0	21308 (14150-29983)	538.37 (357.53-757.56)	7074 (3736-11828)	459.53 (242.68-768.36)	0.53 (0.45 to 0.62)	0
Netherlands	257 (219-299)	7.76 (6.61-9.03)	1162 (1023-1307)	19.85 (17.48-22.34)	3.09 (3.00 to 3.18)	0	15982 (14050-17695)	273.16 (240.14-302.44)	2188 (1972-2441)	66.08 (59.55-73.71)	4.63 (4.12 to 5.14)	0

New Zealand	125 (99-151)	18.94 (15.10-22.93)	412 (352-477)	28.90 (24.68-33.42)	1.37 (1.33 to 1.41)	0	4364 (3890-4785)	305.93 (272.73-335.44)	966 (883-1059)	146.85 (134.16-160.88)	2.50 (2.05 to 2.94)	0
Nicaragua	18 (16-21)	7.41 (6.30-8.60)	121 (107-135)	14.85 (13.11-16.64)	2.29 (2.22 to 2.37)	0	833 (542-1103)	102.35 (66.57-135.46)	181 (138-262)	72.65 (55.70-105.61)	1.06 (0.74 to 1.39)	0
Niger	19 (15-23)	4.31 (3.44-5.23)	38 (31-45)	2.75 (2.26-3.21)	-1.46 (-1.59 to -1.32)	0	1801 (681-3689)	129.36 (48.93-264.94)	831 (303-1471)	188.97 (68.99-334.64)	-1.23 (-1.46 to -1.00)	0
Nigeria	342 (267-427)	4.70 (3.66-5.86)	523 (408-632)	3.48 (2.72-4.21)	-0.97 (-1.01 to -0.93)	0	16619 (6494-30929)	110.78 (43.29-206.17)	10571 (4149-16963)	145.11 (56.95-232.84)	-0.87 (-0.99 to -0.75)	0
Niue	0 (0-0)	9.18 (7.86-10.58)	0 (0-0)	8.60 (7.25-10.08)	-0.21 (-0.26 to -0.16)	0	0 (0-1)	112.04 (66.28-185.75)	1 (0-1)	141.32 (95.85-230.60)	-0.74 (-0.82 to -0.66)	0
North Macedonia	7 (6-9)	2.26 (1.77-2.80)	12 (10-15)	2.03 (1.61-2.51)	-0.35 (-0.37 to -0.33)	0	164 (95-278)	27.74 (16.08-46.94)	82 (61-110)	24.96 (18.51-33.32)	0.38 (0.18 to 0.57)	0
Northern Mariana Islands	0 (0-0)	11.99 (9.22-14.97)	1 (1-1)	12.51 (10.05-15.14)	0.03 (-0.40 to 0.45)	0.906	13 (9-23)	138.50 (87.76-236.81)	5 (4-7)	204.48 (148.07-278.12)	-1.21 (-1.64 to -0.78)	0
Norway	168 (133-204)	15.47 (12.32-18.82)	377 (312-442)	23.24 (19.25-27.24)	1.32 (1.29 to 1.36)	0	4152 (3794-4457)	255.99 (233.93-274.82)	1710 (1586-1845)	157.88 (146.47-170.37)	1.68 (1.01 to 2.34)	0

Oman	6（5-7)	6.08（4.88-7.26)	32（26-39)	10.18（8.27-12.35)	1.66（1.57 to 1.75)	0	249（142-405)	79.66（45.38-129.66)	62（40-104)	61.82（39.33-103.03)	0.76（0.33 to 1.20)	0.001
Pakistan	1802（1446-2189)	19.76（15.85-24.00)	3411（2827-4054)	17.20（14.26-20.45)	-0.45（-0.48 to -0.41)	0	48564（32555-67774)	244.95（164.20-341.84)	23814（15050-34360)	261.13（165.03-376.78)	-0.20（-0.29 to -0.11)	0
Palau	0（0-0)	9.86（8.23-11.69)	0（0-0)	9.15（7.57-10.96)	-0.25（-0.30 to -0.20)	0	4（3-7)	97.77（62.51-161.14)	2（1-3)	122.96（82.13-198.62)	-0.75（-0.85 to -0.66)	0
Palestine	35（29-41)	24.20（20.07-28.56)	137（119-158)	31.94（27.59-36.73)	0.90（0.84 to 0.96)	0	1502（1033-1974)	349.07（240.03-458.82)	552（386-754)	380.25（265.71-519.16)	-0.30（-0.43 to -0.17)	0
Panama	22（19-25)	9.16（7.98-10.43)	147（132-163)	19.80（17.71-21.97)	2.53（2.49 to 2.56)	0	2119（1665-2527)	284.93（223.92-339.80)	268（243-295)	110.50（100.21-121.55)	3.23（2.65 to 3.81)	0
Papua New Guinea	37（30-45)	12.50（10.28-15.26)	122（103-142)	14.89（12.57-17.23)	0.57（0.52 to 0.62)	0	1865（1031-3281)	227.08（125.58-399.44)	627（387-1076)	211.81（130.79-363.67)	0.22（0.13 to 0.31)	0
Paraguay	20（17-23)	5.62（4.81-6.32)	86（78-95)	8.70（7.83-9.64)	1.42（1.38 to 1.47)	0	1249（792-1787)	126.15（80.01-180.49)	304（227-461)	83.84（62.55-126.96)	1.34（1.04 to 1.65)	0
Peru	1316（1183-1465)	66.74（59.99-74.31)	7109（6623-7598)	127.09（118.40-135.84)	2.12（2.06 to 2.17)	0	69081（49293-89851)	1235.03（881.26-1606.36)	16942（12359-23057)	859.24（626.80-1169.40)	1.23（0.57 to 1.89)	0

Philippines	152 （118-189)	3.17 （2.46-3.95)	307 （241-376)	2.20 （1.73-2.69)	-1.18 （-1.23 to -1.14)	0	1006 （761-1314)	7.21 （5.46-9.42)	384 （282-526)	8.03 （5.91-11.02)	-0.35 （-0.43 to -0.26)	0
Poland	426 （314-555)	5.52 （4.08-7.21)	716 （569-883)	5.91 （4.69-7.29)	0.23 （0.11 to 0.36)	0	14623 （13299-15963)	120.70 （109.77-131.75)	7266 （6835-7771)	94.29 （88.70-100.84)	0.90 （0.63 to 1.18)	0
Portugal	256 （216-301)	10.48 （8.86-12.33)	869 （780-952)	22.32 （20.04-24.47)	2.48 （2.45 to 2.50)	0	11359 （9972-12419)	291.96 （256.31-319.21)	2097 （1921-2275)	86.00 （78.78-93.33)	4.07 （3.55 to 4.60)	0
Puerto Rico	60 （53-69)	9.96 （8.68-11.35)	208 （185-231)	17.83 （15.83-19.75)	1.90 （1.86 to 1.94)	0	3476 （2904-4062)	297.78 （248.75-347.93)	873 （792-957)	143.82 （130.49-157.65)	2.42 （1.21 to 3.64)	0
Qatar	1 （1-1)	6.95 （5.31-8.87)	11 （9-14)	7.32 （5.60-9.36)	0.16 （0.09 to 0.23)	0	108 （62-174)	70.62 （40.19-113.62)	15 （11-23)	90.55 （64.65-138.55)	-0.88 （-1.55 to -0.20)	0.011
Republic of Korea	750 （632-881)	15.06 （12.70-17.70)	5859 （5120-6701)	35.04 （30.61-40.07)	2.78 （2.60 to 2.95)	0	41479 （26260-54203)	248.03 （157.02-324.11)	9740 （6289-16006)	195.70 （126.37-321.62)	0.71 （0.52 to 0.91)	0
Republic of Moldova	12 （9-15)	1.53 （1.10-1.99)	15 （11-18)	1.38 （1.06-1.74)	-0.33 （-0.37 to -0.29)	0	97 （76-121)	9.17 （7.17-11.47)	435 （382-499)	56.31 （49.48-64.67)	-5.79 （-6.43 to -5.15)	0
Romania	794 （659-940)	15.86 （13.17-18.80)	643 （555-744)	10.71 （9.23-12.38)	-1.24 （-1.33 to -1.14)	0	7756 （6767-8863)	129.15 （112.69-147.59)	12429 （10814-14435)	248.39 （216.12-288.47)	-2.19 （-2.86 to -1.52)	0

Russian Federation	846 （616-1114)	2.68 （1.95-3.53)	705 （533-903)	1.66 （1.25-2.12)	-1.54 （-1.60 to -1.49)	0	17638 （16181-19244)	41.50 （38.07-45.27)	28106 （24639-31106)	89.05 （78.06-98.55)	-2.81 （-4.11 to -1.51)	0
Rwanda	26 （21-32)	5.61 （4.47-6.73)	59 （48-71)	5.60 （4.57-6.72)	0.01 （-0.14 to 0.16)	0.877	971 （368-2212)	91.78 （34.78-209.21)	555 （186-981)	118.57 （39.65-209.74)	-0.80 （-0.93 to -0.66)	0
Saint Kitts and Nevis	1 （0-1)	8.46 （7.37-9.64)	2 （1-2)	12.02 （10.46-13.84)	1.14 （1.06 to 1.23)	0	18 （15-22)	140.48 （117.91-168.59)	6 （5-7)	98.45 （82.71-115.52)	1.62 （0.68 to 2.57)	0.001
Saint Lucia	1 （1-1)	8.37 （7.37-9.47)	5 （5-6)	12.08 （10.82-13.30)	1.19 （1.16 to 1.22)	0	82 （67-98)	193.77 （159.38-233.62)	17 （15-20)	118.02 （104.29-134.10)	1.62 （0.44 to 2.81)	0.007
Saint Vincent and the Grenadines	0 （0-1)	3.67 （3.07-4.25)	2 （1-2)	6.29 （5.63-7.01)	1.75 （1.72 to 1.79)	0	26 （23-30)	102.86 （88.83-116.19)	3 （2-3)	21.25 （18.76-23.96)	5.11 （3.50 to 6.73)	0
Samoa	1 （1-2)	8.81 （7.28-10.49)	2 （2-3)	8.86 （7.53-10.37)	0.02 （-0.00 to 0.04)	0.078	31 （18-54)	124.54 （73.81-220.05)	19 （12-34)	134.75 （80.25-232.73)	-0.25 （-0.31 to -0.20)	0
San Marino	0 （0-0)	5.47 （4.57-6.44)	1 （1-1)	5.59 （4.76-6.35)	0.08 （0.03 to 0.13)	0.003	6 （4-10)	50.30 （30.74-82.55)	4 （3-5)	68.67 （49.29-93.47)	-1.15 （-1.63 to -0.67)	0
Sao Tome and Principe	1 （1-1)	8.70 （7.14-10.37)	2 （1-2)	8.56 （7.10-10.17)	-0.06 （-0.09 to -0.02)	0.006	65 （25-135)	354.16 （135.61-739.41)	46 （17-83)	406.43 （149.10-743.94)	-0.42 （-0.60 to -0.24)	0

Saudi Arabia	206 (175-239)	23.14 (19.65-26.80)	1004 (845-1185)	33.36 (28.06-39.36)	1.19 (1.16 to 1.23)	0	11825 (7974-16559)	392.77 (264.85-550.02)	3477 (2268-6050)	390.30 (254.62-679.09)	0.03 (-0.09 to 0.16)	0.585
Senegal	25 (21-30)	4.74 (3.91-5.68)	44 (37-51)	3.38 (2.84-3.96)	-1.09 (-1.12 to -1.06)	0	1796 (715-3595)	139.10 (55.40-278.41)	1018 (371-1713)	192.60 (70.17-324.08)	-1.03 (-1.18 to -0.88)	0
Serbia	65 (51-82)	3.15 (2.47-3.94)	98 (83-115)	3.51 (2.96-4.10)	0.35 (0.31 to 0.40)	0	1517 (946-2150)	54.07 (33.72-76.64)	1067 (775-1439)	51.30 (37.25-69.19)	0.18 (0.06 to 0.30)	0.004
Seychelles	1 (0-1)	5.27 (4.34-6.25)	1 (1-1)	5.89 (4.82-6.94)	0.35 (0.33 to 0.37)	0	4 (2-10)	20.25 (7.47-46.91)	2 (1-6)	23.85 (8.23-58.45)	-0.62 (-0.94 to -0.30)	0
Sierra Leone	14 (12-17)	4.22 (3.48-5.00)	17 (14-20)	2.82 (2.34-3.32)	-1.31 (-1.36 to -1.27)	0	776 (282-1666)	128.41 (46.72-275.87)	607 (214-1114)	183.25 (64.50-336.03)	-1.16 (-1.28 to -1.04)	0
Singapore	30 (25-37)	8.35 (6.84-10.10)	201 (174-232)	13.22 (11.45-15.27)	1.50 (1.41 to 1.59)	0	1735 (1548-1929)	114.22 (101.90-126.99)	354 (320-393)	97.64 (88.28-108.30)	0.57 (-0.07 to 1.22)	0.079
Slovakia	40 (32-49)	3.86 (3.05-4.74)	81 (68-96)	4.94 (4.16-5.88)	0.81 (0.76 to 0.86)	0	1155 (686-1733)	70.44 (41.85-105.68)	667 (492-975)	64.25 (47.42-94.03)	0.26 (0.13 to 0.39)	0
Slovenia	16 (13-19)	3.60 (2.93-4.37)	63 (55-73)	8.66 (7.44-9.97)	2.87 (2.69 to 3.06)	0	1186 (1002-1374)	162.05 (136.89-187.69)	413 (368-466)	95.91 (85.34-108.16)	1.68 (0.60 to 2.78)	0.002

Solomon Islands	2 （1-2)	6.81 （5.48-8.22)	4 （3-4)	6.82 （5.74-7.97)	-0.00 （-0.03 to 0.03)	0.813	46 （29-78)	84.98 （52.73-143.49)	18 （12-29)	80.36 （51.06-127.13)	0.13 （-0.13 to 0.40)	0.333
Somalia	19 （15-24)	5.37 （4.22-6.57)	44 （36-52)	4.65 （3.80-5.56)	-0.46 （-0.49 to -0.42)	0	916 （358-1719)	97.14 （37.94-182.28)	394 （123-744)	109.52 （34.21-206.52)	-0.38 （-0.46 to -0.30)	0
South Africa	674 （543-812)	19.98 （16.08-24.05)	1374 （1107-1643)	17.42 （14.03-20.82)	-0.45 （-0.50 to -0.40)	0	16180 （11962-22327)	205.11 （151.64-283.04)	7139 （4130-10591)	211.50 （122.36-313.78)	-0.16 （-0.59 to 0.28)	0.482
South Sudan	27 （22-32)	6.48 （5.28-7.73)	31 （26-36)	4.95 （4.16-5.76)	-0.87 （-0.90 to -0.84)	0	615 （250-1310)	98.68 （40.15-210.32)	519 （181-1073)	125.11 （43.65-258.72)	-0.76 （-0.93 to -0.60)	0
Spain	1954 （1735-2202)	20.44 （18.15-23.03)	5632 （5051-6204)	36.39 （32.64-40.09)	1.88 （1.79 to 1.97)	0	77191 （66055-85034)	498.81 （426.85-549.49)	19058 （17633-20528)	199.34 （184.44-214.72)	3.03 （2.39 to 3.67)	0
Sri Lanka	88 （73-103)	4.95 （4.10-5.80)	362 （308-421)	7.51 （6.40-8.73)	1.37 （1.33 to 1.40)	0	4991 （2993-7991)	103.62 （62.15-165.91)	1985 （1524-2714)	111.54 （85.65-152.47)	-0.17 （-0.64 to 0.30)	0.478
Sudan	97 （78-117)	6.50 （5.21-7.88)	226 （185-266)	7.24 （5.95-8.53)	0.35 （0.33 to 0.37)	0	884 （254-2344)	28.40 （8.16-75.29)	176 （70-357)	11.83 （4.70-24.01)	2.88 （2.70 to 3.07)	0
Suriname	3 （3-3)	6.86 （5.87-7.89)	10 （9-11)	9.12 （8.12-10.29)	0.93 （0.91 to 0.94)	0	128 （76-185)	114.39 （68.17-165.43)	42 （31-62)	95.40 （70.37-142.15)	0.65 （0.24 to 1.06)	0.002

Sweden	388（314-470)	16.35（13.22-19.78)	942（786-1111)	27.99（23.35-32.99)	1.75（1.73 to 1.78)	0	11195（9855-12600)	332.54（292.75-374.28)	4441（4051-4819)	187.07（170.62-203.00)	1.78（0.95 to 2.62)	0
Switzerland	216（192-242)	12.77（11.35-14.31)	505（451-554)	17.22（15.40-18.90)	0.99（0.87 to 1.10)	0	5968（5198-6611)	203.62（177.36-225.57)	2387（2146-2640)	141.32（127.03-156.28)	1.22（0.75 to 1.68)	0
Syrian Arab Republic	71（57-86)	8.05（6.49-9.74)	230（187-276)	9.73（7.92-11.67)	0.61（0.59 to 0.63)	0	2153（1377-3307)	91.13（58.27-139.98)	615（405-924)	69.81（45.97-104.82)	0.87（0.69 to 1.06)	0
Taiwan（Province of China)	126（103-150)	4.53（3.70-5.40)	801（707-914)	10.64（9.39-12.14)	2.81（2.71 to 2.92)	0	7295（6484-8161)	96.88（86.10-108.38)	858（760-970)	30.95（27.41-34.98)	3.69（2.45 to 4.95)	0
Tajikistan	133（116-153)	28.49（24.67-32.73)	322（281-368)	31.33（27.37-35.84)	0.30（0.22 to 0.39)	0	2292（1327-4062)	223.13（129.17-395.51)	1667（884-2848)	355.99（188.69-608.21)	-1.61（-1.81 to -1.41)	0
Thailand	206（166-247)	3.48（2.80-4.16)	634（514-755)	3.26（2.64-3.89)	-0.22（-0.26 to -0.19)	0	3973（2751-6314)	20.44（14.15-32.48)	1534（1116-2146)	25.84（18.81-36.17)	-0.76（-0.99 to -0.52)	0
Timor-Leste	2（2-3)	5.17（4.16-6.20)	9（7-10)	6.02（5.03-7.06)	0.50（0.43 to 0.56)	0	36（11-84)	25.15（7.83-59.27)	9（3-23)	21.29（6.52-53.34)	0.56（0.48 to 0.63)	0
Togo	8（6-10)	4.14（3.30-4.96)	17（13-20)	2.64（2.14-3.14)	-1.45（-1.50 to -1.40)	0	874（324-1816)	139.89（51.94-290.73)	354（135-626)	181.46（69.30-321.11)	-0.83（-1.00 to -0.67)	0

Tokelau	0 (0-0)	9.18 (7.67-10.82)	0 (0-0)	8.73 (7.59-10.04)	-0.15 (-0.21 to -0.09)	0	0 (0-1)	117.09 (69.10-198.61)	0 (0-1)	127.43 (67.26-217.51)	-0.28 (-0.44 to -0.12)	0.001
Tonga	1 (1-1)	6.39 (5.27-7.57)	1 (1-1)	6.95 (6.06-7.95)	0.26 (0.24 to 0.28)	0	12 (7-21)	90.21 (54.52-155.63)	8 (5-14)	88.54 (56.70-144.73)	0.05 (-0.11 to 0.22)	0.513
Trinidad and Tobago	13 (11-14)	9.21 (8.06-10.36)	39 (35-44)	11.24 (9.92-12.61)	0.65 (0.60 to 0.71)	0	785 (612-984)	225.46 (175.83-282.45)	206 (187-225)	149.75 (136.54-163.65)	1.39 (0.90 to 1.88)	0
Tunisia	58 (46-71)	6.62 (5.25-8.10)	186 (151-223)	7.96 (6.48-9.52)	0.60 (0.58 to 0.61)	0	644 (235-1744)	27.57 (10.06-74.61)	102 (49-172)	11.72 (5.67-19.80)	2.80 (2.60 to 2.99)	0
Türkiye	421 (345-502)	7.37 (6.28-8.58)	1622 (1371-1891)	3.17 (2.61-3.77)	1.08 (1.00 to 1.16)	0	276 (217-349)	38.97 (30.73-49.26)	352 (298-394)	108.53 (91.77-121.32)	0.74 (0.56 to 0.92)	0
Turkmenistan	24 (20-28)	8.48 (7.00-10.20)	22 (18-27)	8.82 (7.55-10.34)	-2.70 (-2.76 to -2.63)	0	2 (1-4)	114.81 (62.31-202.14)	2 (1-3)	134.05 (73.35-238.47)	-3.20 (-3.87 to -2.54)	0
Tuvalu	0 (0-0)	7.05 (5.78-8.40)	0 (0-0)	9.82 (8.30-11.45)	0.13 (0.11 to 0.14)	0	18932 (12885-26386)	114.59 (77.99-159.71)	5434 (3299-8725)	90.95 (55.22-146.04)	-0.50 (-0.53 to -0.48)	0
Uganda	64 (51-77)	6.09 (4.91-7.33)	135 (111-160)	5.71 (4.68-6.77)	-0.22 (-0.26 to -0.17)	0	2346 (887-5492)	98.94 (37.41-231.60)	1173 (412-2334)	112.12 (39.36-223.14)	-0.41 (-0.50 to -0.33)	0

Ukraine	978 （717-1295)	7.84 （5.75-10.38)	310 （238-384)	2.28 （1.75-2.83)	-3.92 （-4.05 to -3.79)	0	4249 （3290-5426)	31.29 （24.23-39.96)	16703 （14624-18513)	133.98 （117.30-148.50)	-4.61 （-5.03 to -4.19)	0
United Arab Emirates	5 （4-6)	8.78 （6.75-11.04)	60 （42-79)	8.35 （5.90-11.02)	-0.17 （-0.24 to -0.11)	0	1762 （1232-2508)	245.35 （171.51-349.30)	277 （166-449)	493.18 （294.94-800.29)	-2.38 （-3.90 to -0.83)	0.003
United Kingdom	3404 （2810-4071)	22.92 （18.92-27.41)	9693 （8310-11097)	46.09 （39.51-52.76)	2.26 （2.19 to 2.33)	0	130945 （120038-137859)	622.65 （570.79-655.53)	35085 （33058-37166)	236.22 （222.57-250.22)	3.11 （2.57 to 3.65)	0
United Republic of Tanzania	76 （61-91)	4.21 （3.40-5.03)	186 （153-218)	4.50 （3.72-5.30)	0.22 （0.19 to 0.25)	0	3140 （1195-6808)	76.18 （28.99-165.14)	1404 （540-2619)	77.82 （29.93-145.17)	-0.08 （-0.16 to 0.01)	0.067
United States of America	16848 （13510-20265)	32.12 （25.75-38.63)	48686 （41030-57254)	48.57 （40.93-57.11)	1.39 （1.27 to 1.52)	0	462811 （418504-494382)	461.67 （417.47-493.16)	142475 （131476-153960)	271.58 （250.61-293.47)	1.73 （1.38 to 2.09)	0
United States Virgin Islands	2 （1-2)	11.39 （9.78-13.12)	6 （5-7)	18.80 （16.83-21.05)	1.63 （1.59 to 1.66)	0	42 （30-61)	131.18 （93.64-189.93)	21 （16-31)	150.59 （112.98-220.23)	-0.47 （-0.91 to -0.02)	0.041
Uruguay	103 （89-120)	15.27 （13.15-17.81)	246 （225-268)	27.25 （24.87-29.60)	1.89 （1.86 to 1.93)	0	2902 （2631-3154)	320.86 （290.98-348.82)	1003 （911-1094)	148.65 （135.03-162.10)	2.62 （2.32 to 2.92)	0
Uzbekistan	366 （311-422)	18.72 （15.91-21.59)	573 （506-650)	12.33 （10.89-13.98)	-1.33 （-1.42 to -1.24)	0	2587 （2177-3119)	55.65 （46.83-67.10)	4400 （3436-5693)	225.00 （175.67-291.10)	-4.36 （-5.24 to -3.48)	0

Vanuatu	1 （1-1)	8.36 （6.79-10.01)	2 （2-3)	7.64 （6.40-8.90)	-0.29 （-0.32 to -0.26)	0	34 （19-61)	119.77 （65.44-214.06)	12 （7-20)	123.36 （67.97-204.89)	-0.10 （-0.28 to 0.08)	0.292
Venezuela （Bolivarian Republic of)	132 （115-149)	8.40 （7.32-9.54)	624 （565-698)	11.92 （10.80-13.33)	1.14 （1.10 to 1.17)	0	7370 （5709-9351)	140.86 （109.11-178.72)	1372 （1233-1515)	87.62 （78.71-96.77)	1.54 （0.82 to 2.27)	0
Viet Nam	344 （279-410)	4.93 （4.00-5.88)	1245 （1018-1469)	7.13 （5.83-8.41)	1.20 （1.16 to 1.24)	0	4773 （1720-10690)	27.32 （9.85-61.20)	1565 （494-3886)	22.45 （7.09-55.73)	0.64 （0.56 to 0.73)	0
Yemen	48 （38-59)	5.98 （4.72-7.33)	154 （125-183)	6.84 （5.58-8.14)	0.43 （0.39 to 0.47)	0	586 （174-1444)	26.07 （7.72-64.22)	89 （36-179)	11.09 （4.46-22.26)	2.76 （2.45 to 3.08)	0
Zambia	24 （19-28)	5.13 （4.22-6.13)	57 （47-67)	5.30 （4.36-6.21)	0.10 （0.08 to 0.13)	0	1085 （436-2145)	100.48 （40.41-198.62)	409 （163-719)	88.92 （35.34-156.11)	0.40 （0.31 to 0.49)	0
Zimbabwe	40 （33-48)	6.01 （4.93-7.18)	61 （52-72)	5.52 （4.66-6.52)	-0.27 （-0.30 to -0.23)	0	603 （177-1157)	54.42 （16.02-104.52)	342 （104-604)	51.58 （15.63-91.06)	0.24 （-0.00 to 0.48)	0.052
