# Supplementary material for: Global burden, trends and projections analysis of interstitial lung disease and pulmonary sarcoidosis in elderly adults (aged 55+ Years) based on GBD 2021
Source: PLoS One. 2026 Apr 20;21(4):e0347482. doi: 10.1371/journal.pone.0347482 (PMC13095001; doi:10.1371/journal.pone.0347482)
Supplement: S4 Table — (PDF) [file pone.0347482.s004.pdf]

Supplementary Table 4 Predicted and Observed Values from BAPC Retrospective Validation (1990 – 2021).

	Incidence					Prevalence					Deaths				
Time	pred	sd	low_95	up_95	obs	pred	sd	low_95	up_95	obs	pred	sd	low_95	up_95	obs
1990	14.302	0.046	14.211	14.392	14.296	192.169	0.174	191.828	192.509	192.158	8.049	0.035	7.980	8.118	8.073
1991	14.469	0.045	14.380	14.557	14.460	192.883	0.171	192.547	193.219	192.860	8.168	0.033	8.103	8.233	8.163
1992	14.652	0.045	14.564	14.740	14.644	193.806	0.170	193.473	194.138	193.785	8.317	0.033	8.252	8.382	8.308
1993	14.850	0.045	14.763	14.937	14.845	194.905	0.168	194.576	195.235	194.890	8.445	0.033	8.381	8.510	8.423
1994	15.054	0.044	14.967	15.141	15.050	196.070	0.167	195.743	196.397	196.055	8.635	0.033	8.570	8.700	8.606
1995	15.269	0.044	15.182	15.356	15.264	197.475	0.166	197.150	197.800	197.461	8.972	0.033	8.906	9.037	8.996
1996	15.539	0.044	15.452	15.626	15.535	199.551	0.165	199.228	199.874	199.543	9.122	0.033	9.056	9.188	9.126
1997	15.878	0.044	15.791	15.965	15.879	202.477	0.164	202.154	202.799	202.486	9.248	0.033	9.183	9.314	9.228
1998	16.246	0.045	16.159	16.334	16.253	205.716	0.164	205.394	206.038	205.743	9.492	0.034	9.426	9.558	9.478
1999	16.588	0.045	16.500	16.675	16.599	208.558	0.164	208.237	208.879	208.592	9.769	0.034	9.703	9.835	9.767
2000	16.854	0.045	16.767	16.941	16.866	210.575	0.163	210.256	210.894	210.606	10.067	0.034	10.000	10.134	10.085
2001	17.056	0.044	16.969	17.143	17.064	211.926	0.162	211.610	212.243	211.947	10.295	0.034	10.228	10.362	10.307
2002	17.281	0.044	17.195	17.368	17.290	213.650	0.160	213.337	213.964	213.668	10.457	0.034	10.390	10.524	10.440
2003	17.513	0.044	17.427	17.599	17.522	215.449	0.159	215.137	215.760	215.465	10.852	0.035	10.784	10.919	10.874
2004	17.722	0.044	17.636	17.808	17.729	217.007	0.157	216.699	217.316	217.018	11.172	0.035	11.104	11.240	11.211
2005	17.935	0.044	17.849	18.020	17.937	218.596	0.156	218.290	218.901	218.600	11.263	0.034	11.196	11.331	11.274
2006	18.218	0.043	18.133	18.303	18.218	220.882	0.154	220.579	221.185	220.882	11.356	0.034	11.289	11.422	11.351
2007	18.642	0.043	18.557	18.726	18.645	224.703	0.153	224.403	225.004	224.707	11.608	0.034	11.541	11.675	11.618
2008	19.105	0.043	19.020	19.189	19.112	228.947	0.153	228.648	229.246	228.956	11.785	0.034	11.719	11.852	11.791
2009	19.491	0.043	19.406	19.575	19.500	232.398	0.151	232.102	232.695	232.409	11.887	0.034	11.821	11.953	11.875
2010	19.711	0.043	19.627	19.794	19.718	234.144	0.150	233.851	234.438	234.151	12.097	0.033	12.031	12.162	12.088

2011	19.772	0.042	19.690	19.854	19.774	234.145	0.147	233.856	234.434	234.144	12.363	0.033	12.298	12.428	12.365
2012	19.814	0.041	19.733	19.895	19.815	233.809	0.145	233.524	234.093	233.804	12.532	0.033	12.467	12.596	12.533
2013	19.838	0.041	19.758	19.918	19.838	233.223	0.143	232.943	233.502	233.216	12.681	0.033	12.617	12.745	12.682
2014	19.827	0.040	19.748	19.906	19.823	232.351	0.140	232.076	232.626	232.342	12.839	0.033	12.776	12.903	12.842
2015	19.812	0.040	19.734	19.890	19.804	231.461	0.138	231.190	231.732	231.451	12.884	0.032	12.821	12.947	12.880
2016	19.777	0.039	19.700	19.854	19.761	230.323	0.136	230.057	230.590	230.308	12.949	0.032	12.885	13.012	12.941
2017	19.748	0.262	19.235	20.261	19.732	228.935	2.642	223.756	234.114	229.395	13.025	0.143	12.744	13.306	13.024
2018	19.860	0.317	19.239	20.481	19.712	229.063	3.237	222.718	235.408	228.675	13.124	0.181	12.768	13.480	13.115
2019	19.968	0.369	19.243	20.692	19.679	229.154	3.789	221.728	236.581	228.086	13.218	0.216	12.795	13.640	13.136
2020	20.069	0.422	19.242	20.896	19.665	229.185	4.325	220.708	237.662	227.891	13.306	0.248	12.820	13.791	12.816
2021	20.162	0.477	19.227	21.096	19.561	229.137	4.866	219.599	238.675	227.508	13.388	0.279	12.841	13.935	12.644
