# Supplementary material for: Global burden, trends and projections analysis of interstitial lung disease and pulmonary sarcoidosis in elderly adults (aged 55+ Years) based on GBD 2021
Source: PLoS One. 2026 Apr 20;21(4):e0347482. doi: 10.1371/journal.pone.0347482 (PMC13095001; doi:10.1371/journal.pone.0347482)
Supplement: S5 Table — (PDF) [file pone.0347482.s005.pdf]

Supplementary Table 5 Model performance evaluation.

	MAE (per 100,000 population)	RMSE (per 100,000 population)	MAPE(%)
Incidence	0.291	0.355	1.485
Prevalence	0.968	1.080	0.425
Deaths	0.265	0.400	2.081
