# Supplementary material for: Global burden, trends and projections analysis of interstitial lung disease and pulmonary sarcoidosis in elderly adults (aged 55+ Years) based on GBD 2021
Source: PLoS One. 2026 Apr 20;21(4):e0347482. doi: 10.1371/journal.pone.0347482 (PMC13095001; doi:10.1371/journal.pone.0347482)
Supplement: S6 Table — Abbreviations: BAPC, Bayesian age–period–cohort. (PDF) [file pone.0347482.s006.pdf]

	Incidence				Prevalence				Deaths			
Time	val	sd	low_95	up_95	val	sd	low_95	up_95	val	sd	low_95	up_95
1990	14.307	0.047	14.215	14.398	192.174	0.174	191.833	192.515	8.065	0.036	7.994	8.136
1991	14.470	0.046	14.380	14.560	192.884	0.172	192.548	193.221	8.172	0.035	8.104	8.240
1992	14.652	0.046	14.562	14.741	193.806	0.170	193.474	194.139	8.318	0.034	8.251	8.386
1993	14.850	0.046	14.761	14.939	194.906	0.168	194.576	195.236	8.441	0.034	8.373	8.508
1994	15.053	0.045	14.964	15.142	196.070	0.167	195.743	196.398	8.628	0.034	8.560	8.695
1995	15.268	0.045	15.179	15.356	197.475	0.166	197.150	197.800	8.979	0.035	8.911	9.047
1996	15.537	0.045	15.449	15.626	199.551	0.165	199.228	199.875	9.123	0.035	9.055	9.191
1997	15.878	0.045	15.790	15.967	202.478	0.165	202.155	202.801	9.242	0.035	9.174	9.310
1998	16.248	0.045	16.159	16.337	205.718	0.164	205.396	206.041	9.488	0.035	9.419	9.556
1999	16.591	0.045	16.502	16.680	208.561	0.164	208.240	208.882	9.767	0.035	9.698	9.836
2000	16.857	0.045	16.768	16.946	210.577	0.163	210.258	210.896	10.069	0.035	10.000	10.139
2001	17.057	0.045	16.969	17.146	211.927	0.162	211.610	212.244	10.296	0.035	10.227	10.365
2002	17.283	0.045	17.195	17.371	213.651	0.160	213.337	213.965	10.450	0.035	10.381	10.519
2003	17.514	0.045	17.427	17.602	215.449	0.159	215.137	215.761	10.856	0.036	10.787	10.926
2004	17.722	0.044	17.635	17.809	217.007	0.157	216.698	217.315	11.182	0.036	11.112	11.252
2005	17.933	0.044	17.847	18.020	218.594	0.156	218.288	218.900	11.264	0.035	11.194	11.333
2006	18.215	0.044	18.129	18.302	220.880	0.155	220.577	221.183	11.350	0.035	11.282	11.419
2007	18.640	0.044	18.555	18.726	224.701	0.154	224.400	225.002	11.608	0.035	11.539	11.676
2008	19.105	0.044	19.020	19.191	228.944	0.153	228.645	229.244	11.784	0.035	11.716	11.852
2009	19.492	0.043	19.407	19.577	232.395	0.151	232.098	232.692	11.879	0.034	11.812	11.947
2010	19.711	0.043	19.627	19.795	234.141	0.150	233.847	234.434	12.090	0.034	12.024	12.157
2011	19.770	0.042	19.687	19.853	234.140	0.148	233.851	234.429	12.361	0.034	12.294	12.427
2012	19.811	0.042	19.729	19.893	233.804	0.145	233.519	234.088	12.529	0.034	12.463	12.595

2013	19.835	0.041	19.754	19.915	233.218	0.143	232.938	233.497	12.678	0.033	12.613	12.744
2014	19.821	0.041	19.742	19.901	232.345	0.140	232.070	232.620	12.838	0.033	12.772	12.903
2015	19.803	0.040	19.725	19.882	231.454	0.138	231.184	231.725	12.879	0.033	12.815	12.943
2016	19.762	0.039	19.685	19.839	230.312	0.136	230.046	230.578	12.941	0.032	12.878	13.005
2017	19.734	0.039	19.658	19.810	229.399	0.133	229.137	229.660	13.024	0.032	12.961	13.086
2018	19.714	0.038	19.640	19.789	228.679	0.131	228.422	228.935	13.112	0.032	13.050	13.174
2019	19.683	0.037	19.609	19.756	228.091	0.129	227.839	228.343	13.128	0.031	13.067	13.189
2020	19.670	0.037	19.597	19.742	227.897	0.127	227.648	228.145	12.825	0.030	12.766	12.884
2021	19.571	0.037	19.500	19.643	227.516	0.125	227.271	227.761	12.663	0.030	12.604	12.722
2022	19.324	0.330	18.678	19.970	224.389	2.692	219.112	229.665	12.714	0.177	12.367	13.060
2023	19.393	0.395	18.618	20.168	223.982	3.238	217.637	230.328	12.755	0.223	12.318	13.192
2024	19.460	0.458	18.563	20.357	223.541	3.744	216.203	230.878	12.790	0.263	12.274	13.307
2025	19.523	0.519	18.506	20.539	223.033	4.234	214.734	231.332	12.819	0.300	12.230	13.408
2026	19.579	0.581	18.441	20.717	222.426	4.728	213.159	231.693	12.842	0.336	12.184	13.500
2027	19.631	0.645	18.366	20.895	221.730	5.240	211.459	232.001	12.860	0.370	12.134	13.585
2028	19.681	0.713	18.284	21.079	221.005	5.778	209.681	232.329	12.875	0.404	12.084	13.667
2029	19.731	0.785	18.192	21.270	220.261	6.349	207.816	232.706	12.889	0.437	12.031	13.746
2030	19.777	0.863	18.087	21.468	219.476	6.966	205.823	233.129	12.899	0.471	11.976	13.822
2031	19.818	0.946	17.963	21.673	218.620	7.638	203.650	233.589	12.906	0.505	11.915	13.896
2032	19.854	1.038	17.820	21.888	217.690	8.374	201.277	234.104	12.905	0.541	11.845	13.965
2033	19.889	1.138	17.660	22.119	216.742	9.180	198.750	234.735	12.905	0.578	11.772	14.038
2034	19.925	1.246	17.483	22.367	215.795	10.058	196.081	235.509	12.907	0.617	11.697	14.117
2035	19.959	1.364	17.286	22.631	214.838	11.015	193.248	236.429	12.911	0.660	11.618	14.203
