# Supplementary material for: Global burden, trends and projections analysis of interstitial lung disease and pulmonary sarcoidosis in elderly adults (aged 55+ Years) based on GBD 2021
Source: PLoS One. 2026 Apr 20;21(4):e0347482. doi: 10.1371/journal.pone.0347482 (PMC13095001; doi:10.1371/journal.pone.0347482)
Supplement: S7 Table — Abbreviations: BAPC, Bayesian age–period–cohort. (PDF) [file pone.0347482.s007.pdf]

		Incidence		Prevalence		Deaths	
group	Time	The number of cases	Rate (per 100 000 population)	The number of cases	Rate (per 100 000 population)	The number of cases	Rate (per 100 000 population)
55-59 years	1990	17870.299	9.649	213781.553	115.433	3871.031	2.090
	1991	18187.139	9.682	216127.828	115.059	3919.057	2.086
	1992	18537.047	9.700	219163.661	114.683	3968.369	2.077
	1993	18868.344	9.714	222192.831	114.387	4047.702	2.084
	1994	19144.716	9.718	224807.259	114.113	4130.422	2.097
	1995	19444.110	9.744	227656.434	114.087	4244.834	2.127
	1996	19791.091	9.829	230555.938	114.507	4296.926	2.134
	1997	20170.130	9.965	233246.943	115.234	4320.114	2.134
	1998	20630.902	10.153	236461.337	116.371	4397.020	2.164
	1999	21155.045	10.362	240227.120	117.663	4501.394	2.205
	2000	21464.545	10.468	242234.552	118.136	4617.275	2.252
	2001	21751.693	10.447	245242.795	117.785	4720.342	2.267
	2002	22723.190	10.499	256840.111	118.667	4858.450	2.245
	2003	23716.220	10.519	268918.445	119.278	5040.835	2.236
	2004	24880.623	10.449	283412.271	119.024	5228.762	2.196
	2005	25978.947	10.448	296620.725	119.291	5396.437	2.170
	2006	27477.624	10.505	312265.918	119.379	5593.283	2.138
	2007	28629.514	10.566	321806.076	118.766	5745.353	2.120
	2008	29941.341	10.640	332260.005	118.078	5908.680	2.100
	2009	31103.566	10.726	341386.509	117.723	6097.806	2.103
	2010	32406.274	10.726	353660.431	117.057	6363.743	2.106
	2011	33108.329	10.676	361206.103	116.471	6559.076	2.115
	2012	33646.914	10.617	366881.072	115.762	6762.773	2.134
	2013	34051.211	10.580	371051.865	115.285	6935.769	2.155
	2014	34377.962	10.561	374160.903	114.948	7119.943	2.187
	2015	34727.482	10.549	377407.798	114.641	7363.553	2.237
	2016	35260.333	10.531	382068.544	114.113	7589.645	2.267
	2017	36031.028	10.490	388484.139	113.106	7774.693	2.264
	2018	37075.381	10.417	397740.663	111.749	8098.074	2.275
	2019	38260.631	10.335	409143.798	110.522	8378.204	2.263
	2020	39406.901	10.259	420622.053	109.506	8441.872	2.198
	2021	40477.621	10.229	431205.611	108.965	8564.858	2.164
	2022	41965.186	10.258	444398.625	108.628	8936.044	2.184
	2023	43006.373	10.278	452184.012	108.063	9152.754	2.187
	2024	43892.829	10.298	458215.659	107.508	9336.105	2.190
	2025	44660.026	10.320	462898.264	106.965	9493.833	2.194
	2026	45342.064	10.343	466610.236	106.435	9633.426	2.197
	2027	45971.729	10.367	469703.726	105.921	9762.126	2.201
	2028	46501.449	10.393	471703.922	105.422	9870.098	2.206

	2029	46912.571	10.421	472440.389	104.941	9953.629	2.211
	2030	47279.952	10.451	472683.054	104.480	10028.804	2.217
	2031	47678.240	10.483	473178.430	104.039	10111.617	2.223
	2032	48181.561	10.518	474646.656	103.619	10217.928	2.231
	2033	48758.278	10.557	476752.528	103.222	10341.172	2.239
	2034	49361.664	10.598	479024.202	102.850	10471.598	2.248
	2035	50043.319	10.643	481950.786	102.503	10620.320	2.259
60-64 years	1990	20079.588	12.502	255002.577	158.772	5859.354	3.648
	1991	20638.631	12.591	259414.290	158.262	6020.771	3.673
	1992	21141.812	12.713	262943.119	158.116	6186.816	3.720
	1993	21603.241	12.840	265952.943	158.065	6343.955	3.770
	1994	22041.967	12.972	268856.379	158.230	6464.114	3.804
	1995	22508.991	13.103	272440.401	158.589	6691.876	3.895
	1996	23068.329	13.235	277025.135	158.936	6812.436	3.908
	1997	23753.601	13.382	282733.748	159.280	6910.706	3.893
	1998	24447.628	13.531	288504.791	159.677	7028.276	3.890
	1999	25058.135	13.650	293599.779	159.932	7108.098	3.872
	2000	25639.887	13.760	298842.746	160.383	7247.785	3.890
	2001	26194.778	13.902	304145.224	161.413	7382.709	3.918
	2002	26714.229	14.071	309181.673	162.857	7546.921	3.975
	2003	27320.979	14.295	315132.352	164.883	7877.502	4.122
	2004	28045.582	14.554	322110.012	167.160	8128.522	4.218
	2005	28552.451	14.698	326518.969	168.081	8190.359	4.216
	2006	29272.225	14.788	332797.573	168.126	8330.518	4.208
	2007	31311.016	15.161	352723.100	170.794	8755.716	4.240
	2008	33533.345	15.546	373919.466	173.348	9102.971	4.220
	2009	35948.165	15.749	398043.428	174.380	9399.050	4.118
	2010	37871.228	15.871	418147.669	175.238	9734.797	4.080
	2011	39787.218	15.834	438919.600	174.670	10331.946	4.112
	2012	40664.591	15.613	448277.823	172.116	10650.557	4.089
	2013	41481.737	15.331	457214.412	168.980	10835.637	4.005
	2014	42119.835	15.102	464073.388	166.393	11093.515	3.978
	2015	43259.369	14.890	476521.426	164.015	11513.403	3.963
	2016	44032.568	14.771	484328.206	162.468	11735.218	3.937
	2017	44793.158	14.711	490732.026	161.167	11917.528	3.914
	2018	45566.490	14.746	496531.801	160.680	12328.770	3.990
	2019	46344.738	14.839	502361.668	160.854	12611.672	4.038
	2020	47185.249	14.958	508555.208	161.220	12565.944	3.984
	2021	48039.896	15.010	518736.980	162.081	12719.650	3.974
	2022	47656.457	14.494	520034.940	158.162	13144.969	3.998
	2023	49161.265	14.524	533024.324	157.477	13607.462	4.020
	2024	51054.769	14.551	549785.184	156.693	14151.638	4.033
	2025	53118.840	14.577	567999.261	155.869	14721.977	4.040

	2026	55137.046	14.603	585402.356	155.044	15271.638	4.045
	2027	56893.751	14.630	599758.859	154.230	15750.248	4.050
	2028	58327.482	14.659	610485.692	153.429	16139.832	4.056
	2029	59552.050	14.689	618841.061	152.643	16471.052	4.063
	2030	60615.186	14.721	625366.570	151.874	16757.283	4.070
	2031	61562.380	14.754	630565.409	151.124	17011.547	4.077
	2032	62436.252	14.790	634896.565	150.395	17246.044	4.085
	2033	63172.860	14.828	637728.863	149.689	17443.504	4.094
	2034	63749.695	14.869	638863.646	149.009	17598.090	4.105
	2035	64268.481	14.913	639343.849	148.355	17738.208	4.116
65-69 years	1990	17886.615	14.470	254789.137	206.124	7423.256	6.005
	1991	18649.334	14.635	263026.291	206.409	7696.426	6.040
	1992	19432.637	14.802	271087.747	206.486	7995.994	6.091
	1993	20273.160	15.001	279569.393	206.871	8312.537	6.151
	1994	21086.369	15.187	287432.552	207.018	8675.233	6.248
	1995	21849.704	15.386	294349.205	207.276	9058.220	6.379
	1996	22677.790	15.655	301543.994	208.157	9355.957	6.458
	1997	23558.385	16.029	308750.299	210.070	9668.693	6.578
	1998	24427.127	16.417	315705.140	212.175	10011.204	6.728
	1999	25237.727	16.769	322348.578	214.180	10279.423	6.830
	2000	25987.628	17.035	329151.976	215.758	10617.491	6.960
	2001	26683.158	17.188	336042.136	216.457	10923.253	7.036
	2002	27412.450	17.287	343500.772	216.621	11136.375	7.023
	2003	28132.846	17.381	350793.209	216.723	11482.973	7.094
	2004	28815.069	17.475	357373.315	216.730	11689.163	7.089
	2005	29601.040	17.640	364992.729	217.507	11931.789	7.110
	2006	30722.036	18.047	375615.179	220.642	12158.107	7.142
	2007	32140.297	18.672	388816.108	225.888	12489.931	7.256
	2008	33802.131	19.431	404350.506	232.442	12885.288	7.407
	2009	35491.176	20.160	420444.234	238.822	13324.797	7.569
	2010	36519.237	20.512	429708.644	241.355	13828.232	7.767
	2011	37210.299	20.462	436349.699	239.944	14247.973	7.835
	2012	39143.980	20.576	457634.931	240.551	14743.059	7.750
	2013	41040.198	20.612	478396.119	240.265	15019.768	7.543
	2014	43124.636	20.426	502430.801	237.971	15509.512	7.346
	2015	44990.472	20.360	523470.088	236.894	16112.348	7.292
	2016	47195.038	20.254	547839.765	235.107	17018.394	7.304
	2017	48255.869	19.963	558727.790	231.140	17628.524	7.293
	2018	49310.760	19.627	570079.560	226.904	18460.484	7.348
	2019	50294.133	19.416	580580.732	224.129	18914.791	7.302
	2020	51852.042	19.236	598305.161	221.958	18921.758	7.020
	2021	52569.039	19.058	611036.479	221.517	18957.900	6.873
	2022	52309.432	18.578	613229.315	217.791	19224.972	6.828

	2023	53375.925	18.611	623353.166	217.352	19721.882	6.877
	2024	54209.236	18.670	630514.422	217.152	20174.711	6.948
	2025	55006.845	18.738	636915.540	216.964	20630.559	7.028
	2026	55953.348	18.800	644590.087	216.576	21134.252	7.101
	2027	57244.963	18.847	655666.365	215.873	21740.976	7.158
	2028	59112.517	18.887	672730.481	214.939	22527.693	7.198
	2029	61452.474	18.921	694601.202	213.871	23452.211	7.221
	2030	63995.693	18.955	718271.930	212.748	24420.256	7.233
	2031	66477.183	18.990	740830.029	211.624	25352.359	7.242
	2032	68634.523	19.026	759420.180	210.514	26163.391	7.253
	2033	70400.682	19.064	773386.684	209.423	26826.203	7.264
	2034	71919.611	19.103	784398.016	208.352	27393.948	7.276
	2035	73246.268	19.145	793111.082	207.304	27887.630	7.289
70-74 years	1990	14099.149	16.654	211904.724	250.297	8180.209	9.662
	1991	14806.971	16.878	220199.662	250.999	8529.682	9.723
	1992	15635.502	17.106	230339.923	252.000	9020.661	9.869
	1993	16524.453	17.323	241183.683	252.840	9469.894	9.928
	1994	17477.694	17.617	252645.077	254.663	10022.863	10.103
	1995	18393.229	17.911	262763.331	255.871	10693.525	10.413
	1996	19421.186	18.327	274050.765	258.611	11124.000	10.497
	1997	20567.993	18.810	286439.256	261.962	11584.415	10.594
	1998	21800.899	19.332	299739.603	265.798	12272.777	10.883
	1999	22943.880	19.756	311902.265	268.572	12859.658	11.073
	2000	23923.986	20.084	321987.964	270.310	13324.583	11.186
	2001	24826.780	20.371	330932.227	271.534	13850.032	11.364
	2002	25688.333	20.716	338900.897	273.300	14386.042	11.601
	2003	26531.912	21.072	346434.388	275.145	15178.197	12.055
	2004	27382.625	21.428	354176.383	277.159	15854.565	12.407
	2005	28319.630	21.781	363415.290	279.513	16154.012	12.425
	2006	29482.815	22.192	375877.841	282.922	16379.510	12.329
	2007	30953.412	22.715	392210.075	287.826	17299.565	12.695
	2008	32497.318	23.275	409351.657	293.181	17858.502	12.790
	2009	33882.565	23.739	424195.216	297.204	18208.786	12.758
	2010	35056.244	24.056	436676.197	299.654	18793.865	12.897
	2011	36130.462	24.363	447516.202	301.763	19539.949	13.176
	2012	37167.734	24.722	457382.549	304.225	20091.205	13.364
	2013	38357.462	25.174	468345.762	307.377	20477.654	13.440
	2014	39647.964	25.634	480589.098	310.726	20895.363	13.510
	2015	40566.735	25.861	487485.078	310.774	21273.309	13.562
	2016	41401.927	25.775	493897.145	307.477	22007.875	13.701
	2017	43748.386	25.963	519689.767	308.411	23241.125	13.792
	2018	46062.702	26.075	546143.284	309.156	24466.286	13.850
	2019	48496.930	25.855	576701.595	307.460	25377.362	13.530

	2020	50653.097	25.837	604515.911	308.354	26010.519	13.268
	2021	52636.204	25.572	632735.180	307.393	27278.685	13.252
	2022	51158.743	24.029	626655.310	294.342	26338.287	12.371
	2023	52841.440	23.890	644381.116	291.328	27089.636	12.247
	2024	54480.325	23.769	661326.984	288.533	27853.534	12.152
	2025	56084.509	23.682	677869.641	286.238	28624.758	12.087
	2026	57648.945	23.636	694053.518	284.559	29421.200	12.063
	2027	59157.487	23.633	709553.454	283.465	30265.800	12.091
	2028	60424.196	23.676	721994.036	282.895	31078.547	12.177
	2029	61437.938	23.750	731127.834	282.637	31828.600	12.304
	2030	62419.727	23.837	739472.880	282.395	32588.996	12.445
	2031	63577.113	23.916	749364.550	281.891	33429.557	12.575
	2032	65129.437	23.977	763231.632	280.979	34435.443	12.677
	2033	67343.367	24.027	784129.139	279.766	35730.438	12.748
	2034	70103.963	24.072	810711.715	278.378	37249.078	12.790
	2035	73098.346	24.115	839399.603	276.919	38837.975	12.813
75-79 years	1990	10406.186	16.905	170048.562	276.252	8944.065	14.530
	1991	10687.919	17.302	173071.655	280.181	9150.525	14.814
	1992	10986.555	17.791	175576.585	284.326	9370.770	15.175
	1993	11324.267	18.365	178229.822	289.043	9522.184	15.443
	1994	11736.503	18.867	181643.265	292.004	9807.732	15.767
	1995	12350.689	19.357	189144.130	296.435	10515.594	16.481
	1996	13123.615	19.786	199048.804	300.096	11040.628	16.645
	1997	14072.262	20.291	212123.318	305.863	11577.687	16.694
	1998	15091.303	20.776	226462.527	311.765	12365.122	17.023
	1999	16142.611	21.289	241244.306	318.158	13381.009	17.647
	2000	17092.195	21.696	253527.651	321.815	14425.391	18.311
	2001	18008.865	22.077	264902.469	324.749	15258.402	18.706
	2002	18946.311	22.439	275918.812	326.787	15930.859	18.868
	2003	19958.082	22.849	287528.479	329.172	17004.260	19.467
	2004	20942.173	23.209	298602.600	330.919	18008.336	19.957
	2005	21902.319	23.591	309125.621	332.954	18650.781	20.088
	2006	22986.922	24.100	321928.742	337.514	19289.749	20.224
	2007	24183.351	24.791	337144.833	345.620	20155.979	20.663
	2008	25410.433	25.515	353264.058	354.715	20990.881	21.077
	2009	26573.780	26.140	368684.166	362.660	21692.581	21.338
	2010	27607.740	26.540	382133.214	367.359	22628.856	21.754
	2011	28495.955	26.686	392994.229	368.035	23555.323	22.059
	2012	29410.366	26.751	403336.159	366.862	24311.740	22.113
	2013	30306.920	26.805	412663.589	364.979	25108.658	22.207
	2014	31132.882	26.849	420335.878	362.503	25969.870	22.397
	2015	32010.584	26.949	429201.152	361.341	26523.977	22.330
	2016	33018.350	27.216	440149.352	362.799	27268.889	22.477

	2017	34015.988	27.562	451540.641	365.868	28179.272	22.833
	2018	35188.950	28.045	465344.045	370.872	29356.143	23.396
	2019	36418.434	28.525	481128.171	376.854	30530.137	23.913
	2020	37254.738	28.799	490548.774	379.211	30561.892	23.625
	2021	37657.604	28.553	495529.406	375.730	30715.322	23.290
	2022	38661.701	27.728	514851.698	369.252	31573.946	22.645
	2023	40574.590	27.833	538669.550	369.510	32669.635	22.410
	2024	42819.367	27.866	567056.547	369.030	33992.421	22.122
	2025	45156.614	27.830	596568.552	367.666	35452.075	21.849
	2026	47364.241	27.728	623885.782	365.230	36894.807	21.599
	2027	49266.075	27.575	646448.343	361.833	38141.724	21.349
	2028	50961.221	27.416	665710.512	358.131	39286.947	21.135
	2029	52630.262	27.278	684367.998	354.699	40462.812	20.971
	2030	54271.504	27.178	702671.867	351.880	41653.866	20.859
	2031	55872.651	27.125	720572.454	349.820	42880.706	20.817
	2032	57411.307	27.122	737646.439	348.477	44171.801	20.868
	2033	58717.344	27.171	751558.017	347.779	45418.629	21.017
	2034	59797.021	27.257	762266.685	347.466	46589.813	21.237
	2035	60865.015	27.357	772388.076	347.171	47792.488	21.482
80-84 years	1990	6697.269	18.932	98262.207	277.766	7003.666	19.798
	1991	7012.310	19.216	102880.502	281.930	7372.671	20.204
	1992	7333.624	19.553	107486.182	286.576	7749.122	20.660
	1993	7669.386	19.884	112233.708	290.982	8079.686	20.948
	1994	8002.708	20.288	116919.293	296.406	8507.581	21.568
	1995	8316.379	20.749	121060.859	302.046	9140.744	22.806
	1996	8626.019	21.422	125300.266	311.177	9416.230	23.385
	1997	8990.162	22.283	130160.645	322.609	9620.342	23.844
	1998	9401.717	23.241	135433.518	334.792	10044.167	24.829
	1999	9861.553	24.028	140747.370	342.940	10554.139	25.716
	2000	10466.319	24.661	148522.384	349.948	11297.766	26.620
	2001	11141.667	25.054	156886.395	352.786	12093.481	27.194
	2002	11894.633	25.430	166794.236	356.592	12894.602	27.568
	2003	12664.867	25.749	177180.336	360.221	14194.998	28.859
	2004	13475.622	26.155	188313.325	365.499	15577.013	30.234
	2005	14284.472	26.582	198680.933	369.730	16373.914	30.471
	2006	15166.832	27.139	210357.360	376.400	17244.523	30.856
	2007	16141.643	27.757	223657.305	384.599	18367.177	31.584
	2008	17187.379	28.426	238136.016	393.848	19488.678	32.232
	2009	18160.304	28.945	251430.650	400.746	20445.086	32.587
	2010	19004.532	29.314	262099.455	404.287	21550.776	33.242
	2011	19771.062	29.558	271086.400	405.271	22968.579	34.338
	2012	20468.386	29.798	279004.138	406.181	24128.985	35.128
	2013	21126.186	29.990	286363.434	406.507	25410.749	36.072

	2014	21744.826	30.111	293445.881	406.345	26743.372	37.032
	2015	22410.601	30.190	301584.532	406.276	27533.399	37.091
	2016	23099.680	30.165	310272.872	405.178	28293.710	36.948
	2017	23838.811	30.097	319442.342	403.307	29323.761	37.022
	2018	24582.962	30.054	328482.311	401.592	30228.060	36.956
	2019	25291.234	30.038	336692.325	399.886	31008.059	36.828
	2020	25927.955	30.064	345471.245	400.579	31019.902	35.968
	2021	26390.342	30.132	353011.147	403.059	31220.879	35.647
	2022	28396.077	31.091	371416.649	406.664	32838.491	35.955
	2023	29539.992	31.583	384005.609	410.568	34067.510	36.424
	2024	30654.127	32.053	396122.860	414.204	35260.304	36.870
	2025	31809.242	32.449	408701.915	416.917	36433.736	37.166
	2026	33079.356	32.745	422848.939	418.577	37606.462	37.227
	2027	34551.265	32.952	439885.024	419.520	38829.016	37.031
	2028	36391.390	33.076	461892.192	419.816	40321.021	36.648
	2029	38545.009	33.116	488008.430	419.275	42106.875	36.176
	2030	40779.072	33.074	515048.794	417.729	44055.821	35.731
	2031	42881.558	32.952	540002.331	414.964	45965.902	35.322
	2032	44683.752	32.772	560536.617	411.109	47605.664	34.915
	2033	46301.268	32.582	578234.918	406.906	49120.691	34.566
	2034	47919.315	32.419	595699.795	403.010	50699.197	34.300
	2035	49522.271	32.301	612971.577	399.811	52307.140	34.117
85-89 years	1990	3625.500	23.992	38488.198	254.702	4054.719	26.833
	1991	3851.999	24.371	40614.335	256.956	4312.966	27.287
	1992	4100.815	24.757	43241.706	261.050	4633.320	27.971
	1993	4342.864	25.235	45903.306	266.730	4931.231	28.654
	1994	4587.421	25.714	48709.319	273.035	5293.189	29.670
	1995	4830.816	26.222	51631.206	280.253	5812.561	31.550
	1996	5094.317	26.751	55028.691	288.959	6188.851	32.498
	1997	5391.617	27.435	59005.313	300.243	6528.518	33.220
	1998	5718.383	28.142	63335.345	311.693	6993.349	34.416
	1999	6047.490	28.915	67530.501	322.886	7562.525	36.159
	2000	6355.534	29.708	70992.370	331.839	8139.347	38.046
	2001	6632.087	30.644	73836.468	341.165	8604.728	39.759
	2002	6911.088	31.686	76446.336	350.487	8936.310	40.971
	2003	7204.408	32.772	79078.602	359.721	9565.987	43.515
	2004	7534.702	33.587	81929.050	365.207	10190.313	45.424
	2005	8006.475	34.219	87037.387	371.990	10756.464	45.972
	2006	8574.926	34.644	93359.963	377.188	11554.338	46.681
	2007	9231.879	35.113	101364.401	385.533	12594.485	47.902
	2008	9910.566	35.543	109896.634	394.131	13549.273	48.593
	2009	10610.562	36.036	118624.487	402.882	14468.368	49.139
	2010	11272.290	36.439	126102.301	407.645	15536.625	50.225

	2011	11890.058	36.792	132477.485	409.935	16788.502	51.950
	2012	12512.735	37.028	138389.852	409.530	18071.255	53.477
	2013	13134.783	37.213	144326.967	408.902	19543.237	55.369
	2014	13668.697	37.205	149607.120	407.219	20957.195	57.044
	2015	14162.127	37.197	154644.065	406.175	21897.158	57.513
	2016	14668.420	37.183	160119.582	405.892	22794.170	57.782
	2017	15120.972	37.177	165338.979	406.508	23659.099	58.169
	2018	15579.533	37.162	170626.079	407.000	24314.550	57.998
	2019	16061.441	37.112	175954.755	406.570	25125.530	58.056
	2020	16490.405	36.980	181424.910	406.846	25107.133	56.303
	2021	16788.463	36.719	185449.576	405.604	25225.112	55.171
	2022	19085.735	39.153	201452.052	413.267	28020.438	57.482
	2023	19829.033	39.544	207748.192	414.300	28900.008	57.634
	2024	20642.472	39.939	214745.088	415.488	29853.166	57.760
	2025	21542.213	40.386	222603.494	417.325	30928.186	57.983
	2026	22525.168	40.920	231258.004	420.107	32148.249	58.401
	2027	23565.167	41.538	240399.681	423.749	33490.006	59.032
	2028	24605.073	42.196	249465.795	427.820	34871.804	59.803
	2029	25660.109	42.825	258616.956	431.613	36272.418	60.536
	2030	26775.982	43.354	268320.770	434.444	37689.355	61.024
	2031	28002.394	43.750	279174.939	436.178	39122.454	61.124
	2032	29399.237	44.027	291918.309	437.164	40602.881	60.805
	2033	31126.009	44.194	308114.377	437.477	42382.597	60.177
	2034	33143.399	44.248	327264.457	436.916	44495.499	59.404
	2035	35226.763	44.193	346994.102	435.309	46770.913	58.675
90-94 years	1990	1278.335	29.831	10161.047	237.120	1500.365	35.013
	1991	1379.099	30.132	10954.390	239.345	1638.235	35.794
	1992	1479.598	30.546	11735.951	242.290	1778.564	36.719
	1993	1598.618	31.014	12660.715	245.622	1941.537	37.666
	1994	1724.231	31.500	13626.995	248.951	2131.941	38.948
	1995	1852.108	32.002	14623.141	252.667	2367.192	40.902
	1996	1983.303	32.612	15703.808	258.225	2561.640	42.122
	1997	2135.518	33.327	17137.264	267.444	2784.100	43.449
	1998	2289.856	34.149	18671.371	278.445	3022.657	45.077
	1999	2447.621	34.945	20215.354	288.616	3323.913	47.456
	2000	2604.018	35.722	21686.019	297.485	3667.019	50.304
	2001	2763.196	36.417	23137.277	304.932	3974.453	52.380
	2002	2927.105	37.194	24614.465	312.771	4244.561	53.935
	2003	3098.387	37.933	26159.985	320.268	4674.825	57.232
	2004	3274.674	38.736	27754.859	328.315	5093.414	60.251
	2005	3451.768	39.633	29293.625	336.348	5390.534	61.894
	2006	3629.762	40.795	30844.136	346.655	5676.276	63.795
	2007	3821.551	42.127	32478.095	358.022	6025.857	66.426

	2008	4029.391	43.486	34244.838	369.581	6314.531	68.148
	2009	4262.735	44.383	36102.976	375.900	6615.443	68.879
	2010	4567.690	44.762	38857.687	380.790	7205.315	70.609
	2011	4894.405	44.752	41635.927	380.695	7936.570	72.567
	2012	5244.150	44.741	44660.711	381.029	8732.126	74.499
	2013	5575.025	44.628	47601.628	381.047	9585.430	76.731
	2014	5882.794	44.541	50545.767	382.701	10412.243	78.835
	2015	6180.812	44.494	53384.664	384.304	11074.789	79.725
	2016	6467.413	44.394	56212.296	385.854	11773.101	80.813
	2017	6747.907	44.147	58976.852	385.843	12497.731	81.764
	2018	7044.250	43.906	61844.332	385.472	13145.210	81.933
	2019	7333.249	43.598	64425.905	383.026	13846.047	82.318
	2020	7548.914	43.282	66502.813	381.295	13983.862	80.177
	2021	7699.854	43.041	68081.905	380.572	14153.668	79.118
	2022	9145.262	47.183	77217.481	398.387	16422.684	84.729
	2023	9596.182	47.948	80342.039	401.436	17232.442	86.103
	2024	10135.502	48.685	84165.110	404.277	18156.876	87.214
	2025	10710.748	49.376	88230.974	406.740	19084.676	87.979
	2026	11268.063	49.989	92095.864	408.570	19944.439	88.481
	2027	11773.641	50.539	95485.525	409.880	20699.630	88.855
	2028	12286.426	51.044	98906.208	410.909	21444.107	89.090
	2029	12876.517	51.555	102924.848	412.091	22300.461	89.287
	2030	13532.361	52.133	107441.779	413.916	23266.214	89.632
	2031	14238.479	52.822	112317.522	416.679	24335.626	90.281
	2032	14969.849	53.622	117336.386	420.295	25477.308	91.259
	2033	15709.441	54.472	122375.749	424.337	26662.661	92.452
	2034	16485.899	55.285	127660.176	428.103	27907.755	93.587
	2035	17321.281	55.968	133361.057	430.914	29197.699	94.343
95+ years	1990	363.779	35.732	2234.064	219.438	427.730	42.013
	1991	382.877	36.129	2350.950	221.840	459.702	43.378
	1992	405.082	36.554	2485.444	224.284	496.060	44.764
	1993	428.506	37.041	2626.861	227.070	535.942	46.328
	1994	454.330	37.516	2781.269	229.662	581.063	47.981
	1995	487.060	38.044	2987.398	233.343	646.113	50.467
	1996	528.939	38.633	3264.869	238.460	712.843	52.065
	1997	572.560	39.407	3566.961	245.497	776.744	53.460
	1998	625.516	40.262	3934.712	253.263	862.031	55.486
	1999	683.314	41.101	4315.540	259.576	966.135	58.112
	2000	745.914	41.834	4705.797	263.918	1089.376	61.096
	2001	811.048	42.491	5092.538	266.796	1207.299	63.250
	2002	879.198	43.092	5521.473	270.623	1332.082	65.289
	2003	945.550	43.741	5957.306	275.583	1498.826	69.335
	2004	1013.044	44.325	6415.510	280.703	1663.992	72.806

	2005	1084.621	44.972	6910.409	286.528	1804.913	74.837
	2006	1163.211	45.713	7450.690	292.802	1961.592	77.088
	2007	1250.378	46.647	8050.844	300.346	2134.852	79.643
	2008	1346.964	47.679	8704.274	308.106	2326.322	82.345
	2009	1452.809	48.780	9393.411	315.397	2537.000	85.183
	2010	1558.952	49.849	10059.079	321.646	2770.391	88.585
	2011	1658.303	50.951	10655.819	327.398	3004.472	92.312
	2012	1757.194	52.055	11216.707	332.284	3231.118	95.719
	2013	1852.185	52.952	11791.761	337.114	3453.891	98.743
	2014	1942.516	53.276	12389.370	339.797	3664.692	100.510
	2015	2057.398	53.096	13286.382	342.885	3948.225	101.893
	2016	2172.752	52.590	14221.009	344.209	4281.875	103.639
	2017	2288.169	52.040	15227.609	346.325	4652.220	105.807
	2018	2401.819	51.473	16220.643	347.619	4997.100	107.091
	2019	2524.639	50.982	17240.661	348.154	5409.619	109.241
	2020	2628.237	50.272	18162.133	347.400	5605.962	107.229
	2021	2707.861	49.683	18940.907	347.520	5697.626	104.538
	2022	2983.448	54.307	20245.798	368.529	6206.330	112.972
	2023	3186.233	55.120	21479.136	371.579	6645.630	114.966
	2024	3499.372	55.967	23405.268	374.329	7302.429	116.790
	2025	3823.185	56.858	25352.024	377.031	7968.687	118.509
	2026	4109.966	57.785	27013.368	379.801	8561.113	120.367
	2027	4340.799	58.750	28281.605	382.775	9047.862	122.458
	2028	4613.253	59.704	29803.302	385.708	9615.775	124.445
	2029	4970.980	60.622	31852.190	388.441	10336.353	126.053
	2030	5348.445	61.483	33996.695	390.811	11061.726	127.161
	2031	5703.837	62.248	35971.863	392.573	11718.473	127.888
	2032	6016.143	62.934	37648.413	393.835	12277.285	128.431
	2033	6354.482	63.564	39470.845	394.826	12873.478	128.773
	2034	6766.369	64.201	41732.267	395.966	13602.076	129.060
	2035	7219.552	64.922	44228.314	397.723	14407.781	129.562
