# Supplementary figures and images for: Global burden, trends and projections analysis of interstitial lung disease and pulmonary sarcoidosis in elderly adults (aged 55+ Years) based on GBD 2021
Source: PLoS One. 2026 Apr 20;21(4):e0347482. doi: 10.1371/journal.pone.0347482 (PMC13095001; doi:10.1371/journal.pone.0347482)

A

<55 years  
n=105380.2 (27%)

55+ years  
n=284886.9 (73%)

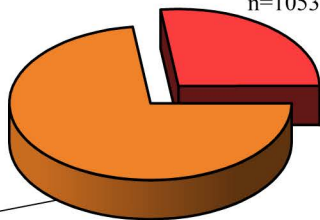

B

<55 years  
n=991948.6 (23%)

55+ years  
n=3314679.1 (77%)

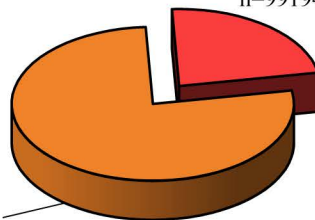

C

<55 years  
n=13854.5 (7.4%)

55+ years  
n=174367.8 (92.6%)

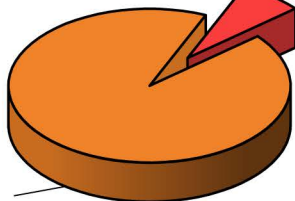

D

<55 years  
n=757896.8 (18.7%)

55+ years  
n=3284253.7 (81.3%)

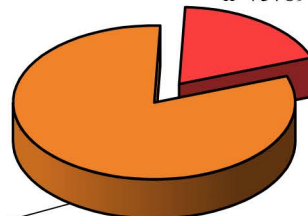

Supplement: S1 Fig — Abbreviations: ILD&PS, Interstitial lung disease and pulmonary sarcoidosis; DALYs, disability-adjusted life-years. (PDF) [file pone.0347482.s008.pdf]

A

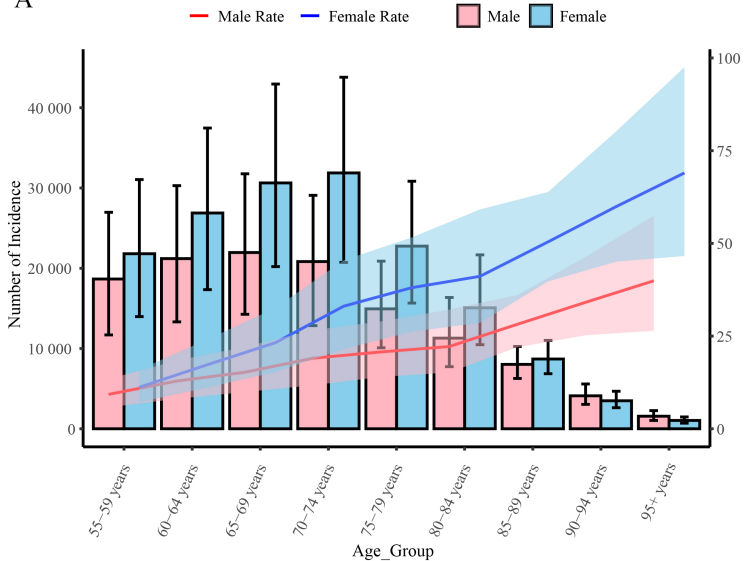

B

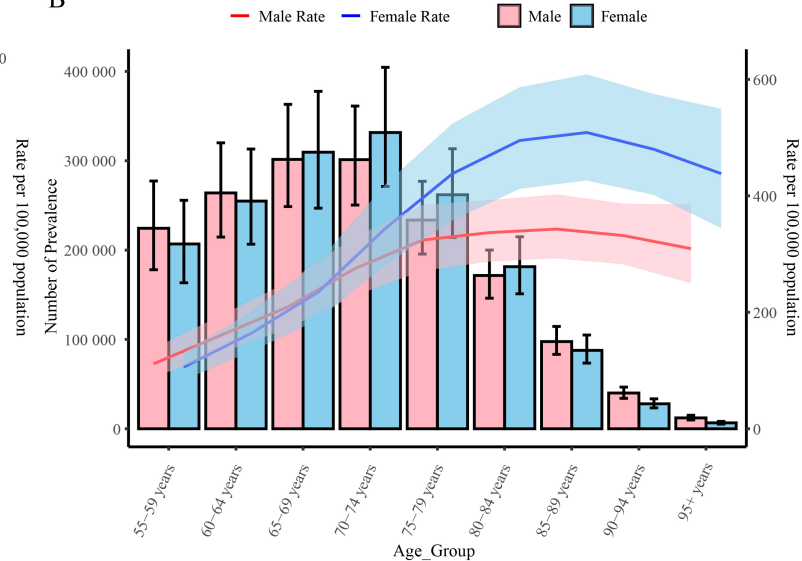

C

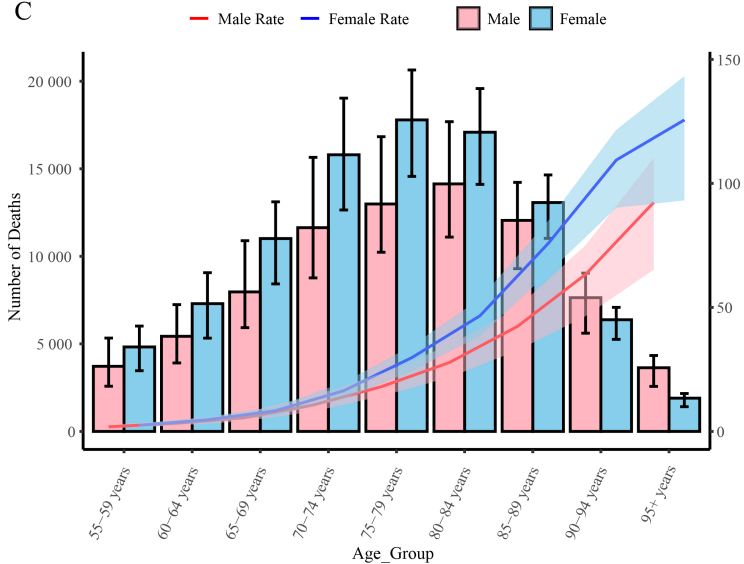

D

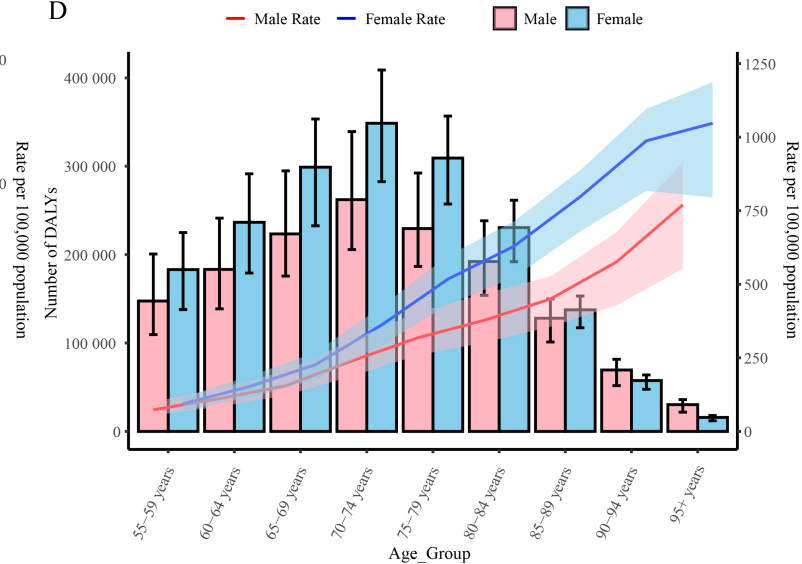

Supplement: S2 Fig — Abbreviations: ILD&PS, Interstitial lung disease and pulmonary sarcoidosis; DALYs, disability-adjusted life-years. (PDF) [file pone.0347482.s009.pdf]

A

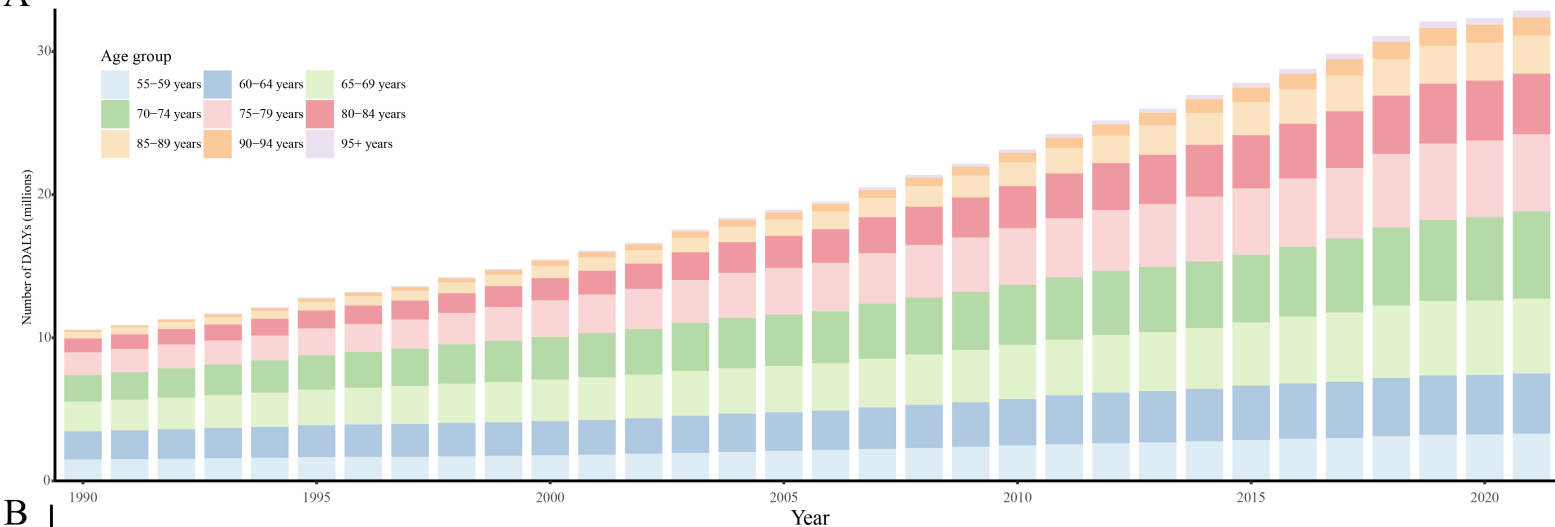

B

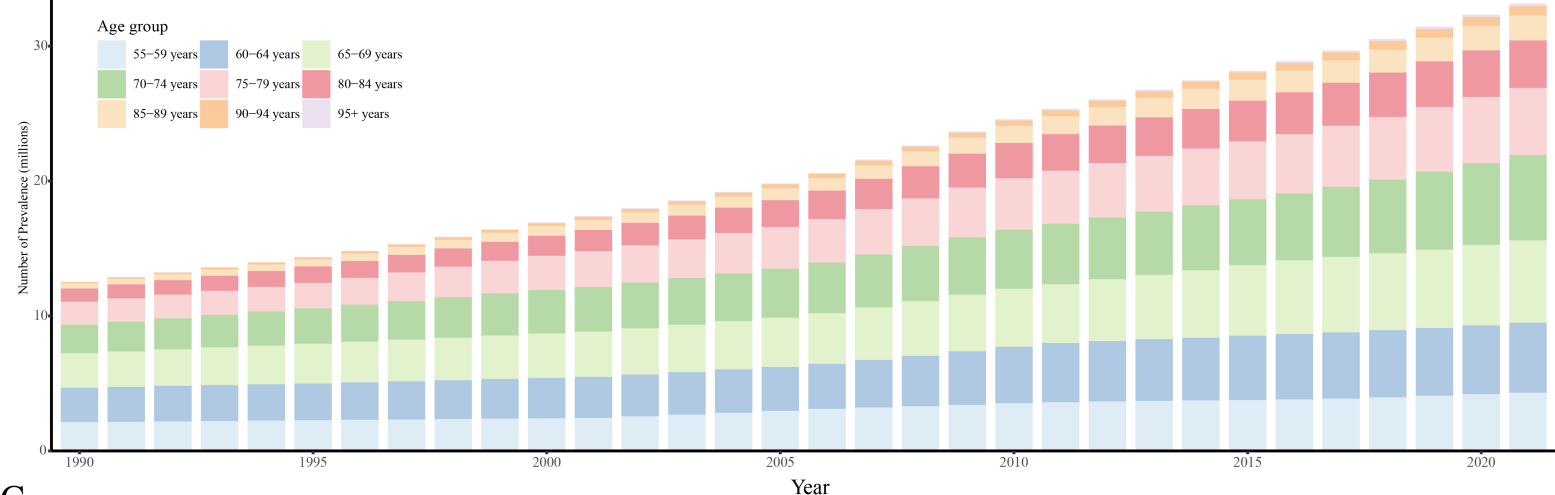

C

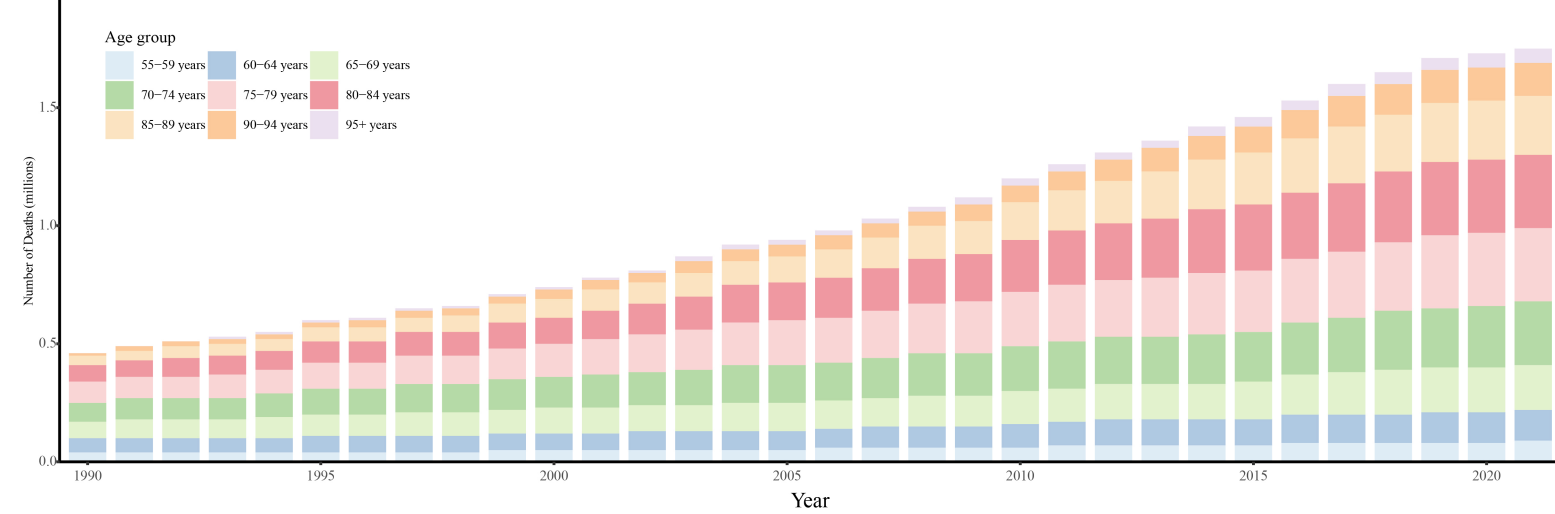

Supplement: S3 Fig — Abbreviations: ILD&PS, Interstitial lung disease and pulmonary sarcoidosis; DALYs, disability-adjusted life-years. (PDF) [file pone.0347482.s010.pdf]

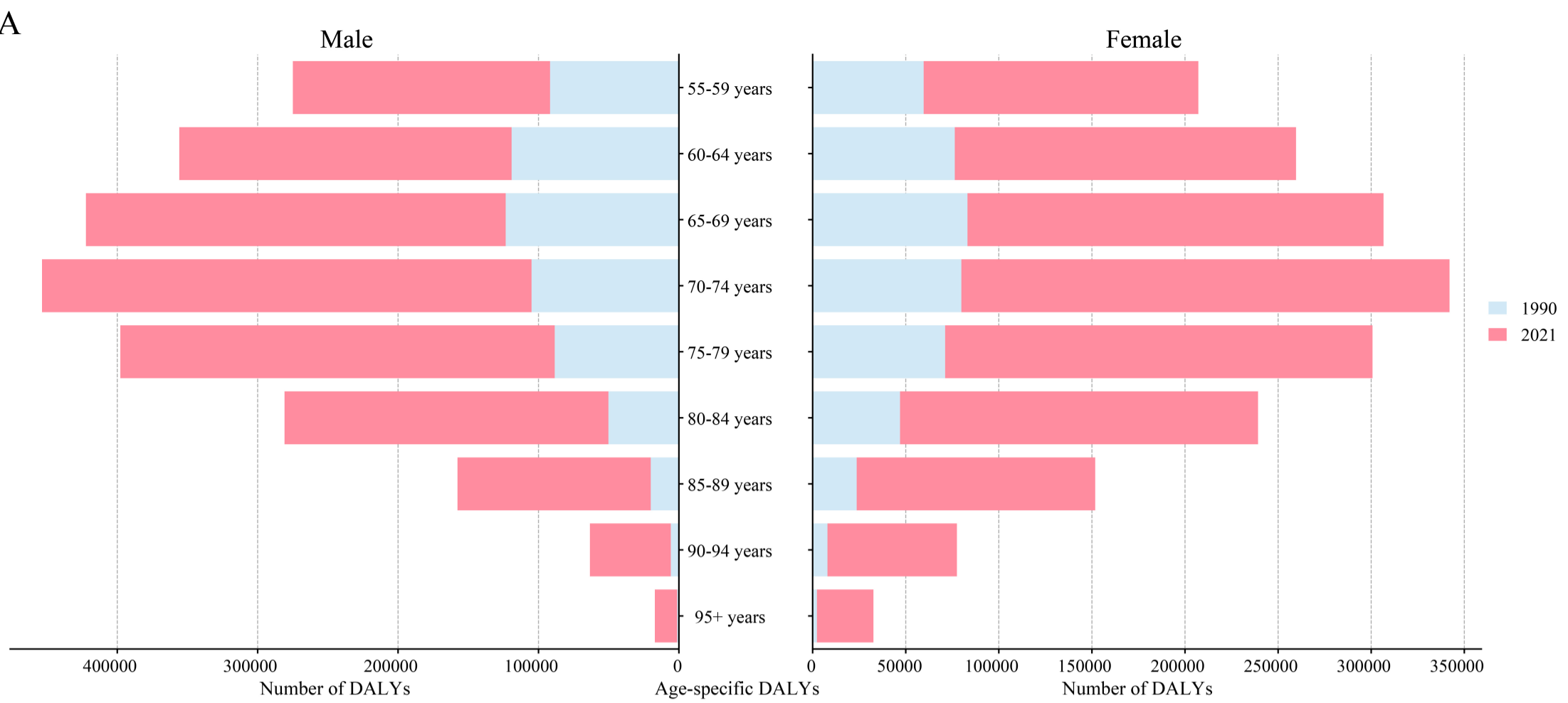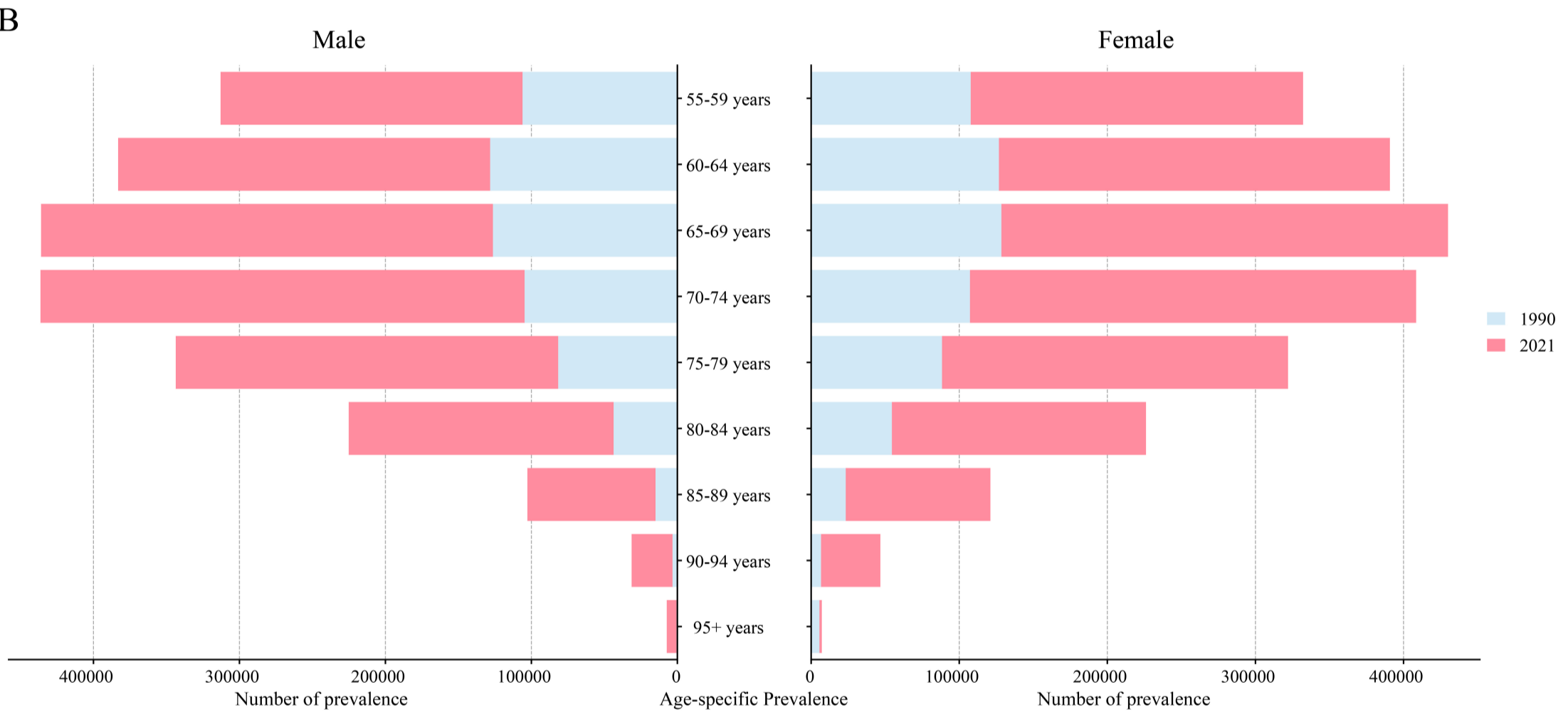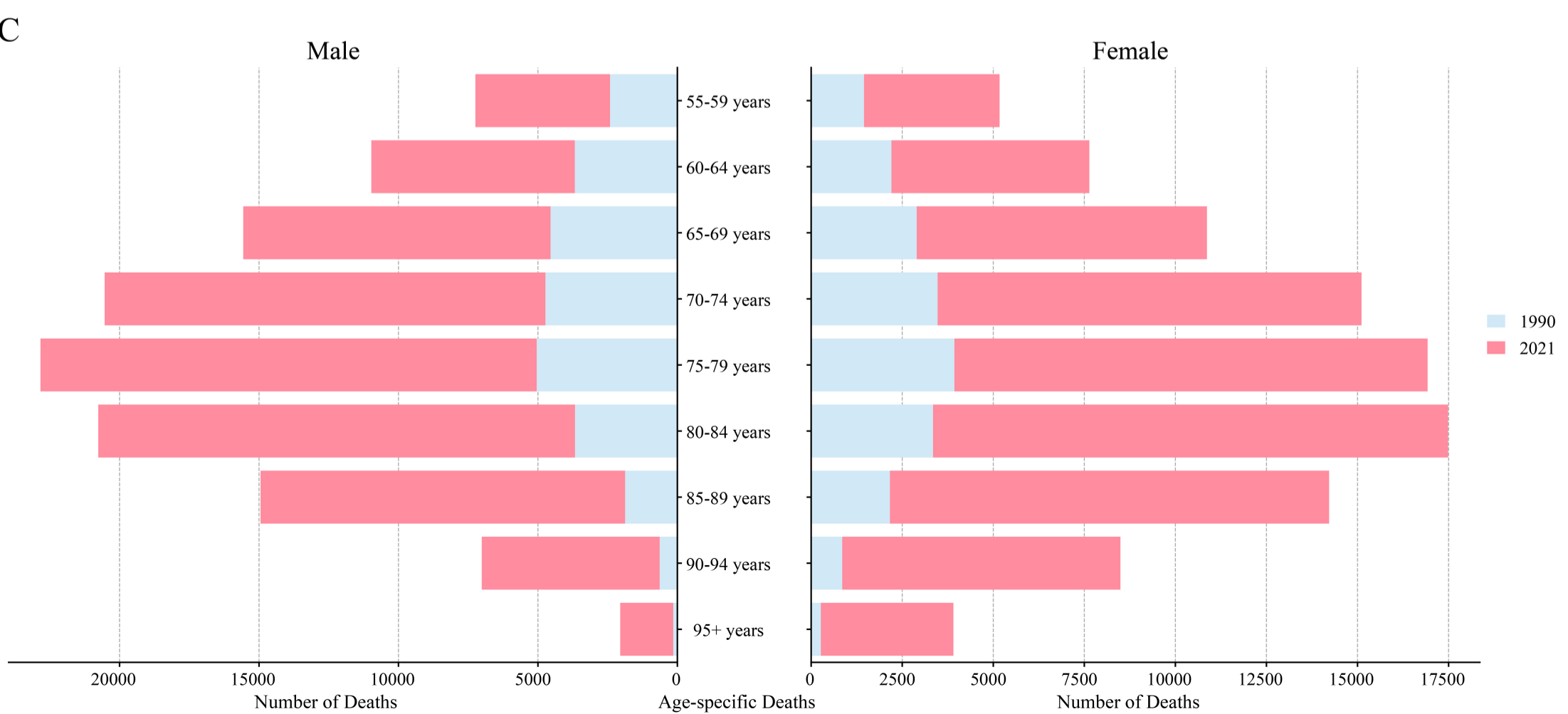

Supplement: S4 Fig — Abbreviations: ILD&PS, Interstitial lung disease and pulmonary sarcoidosis; DALYs, disability-adjusted life-years. (PDF) [file pone.0347482.s011.pdf]

A

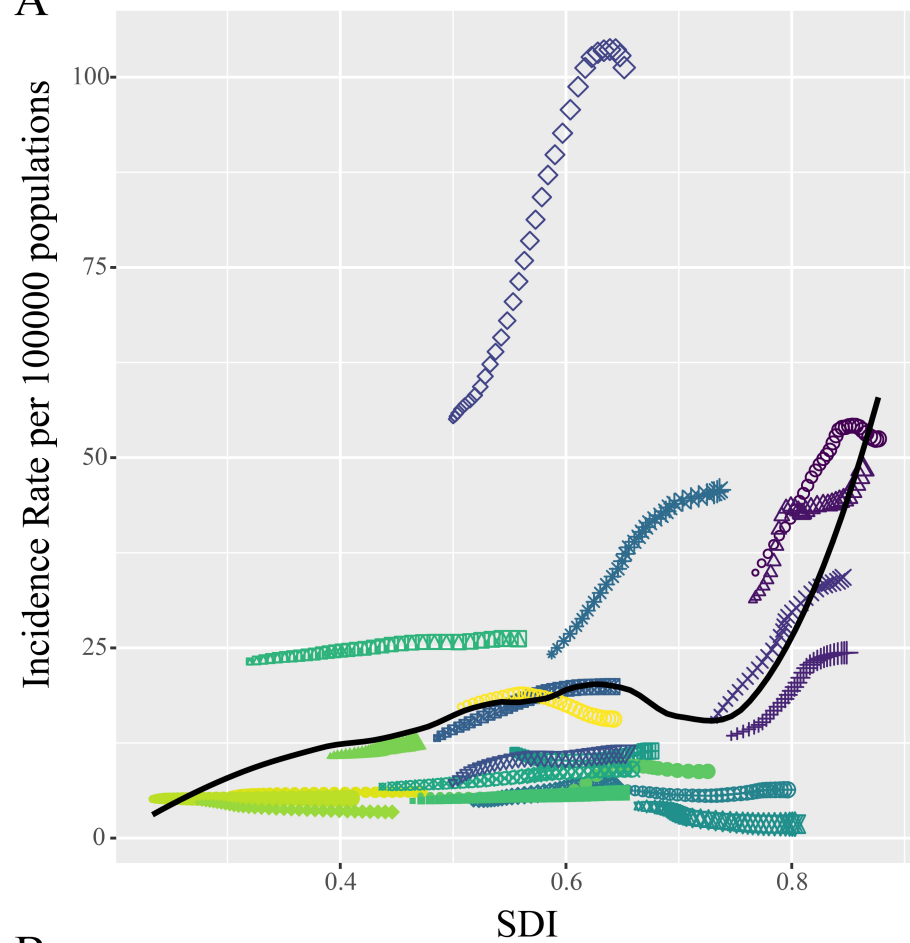

B

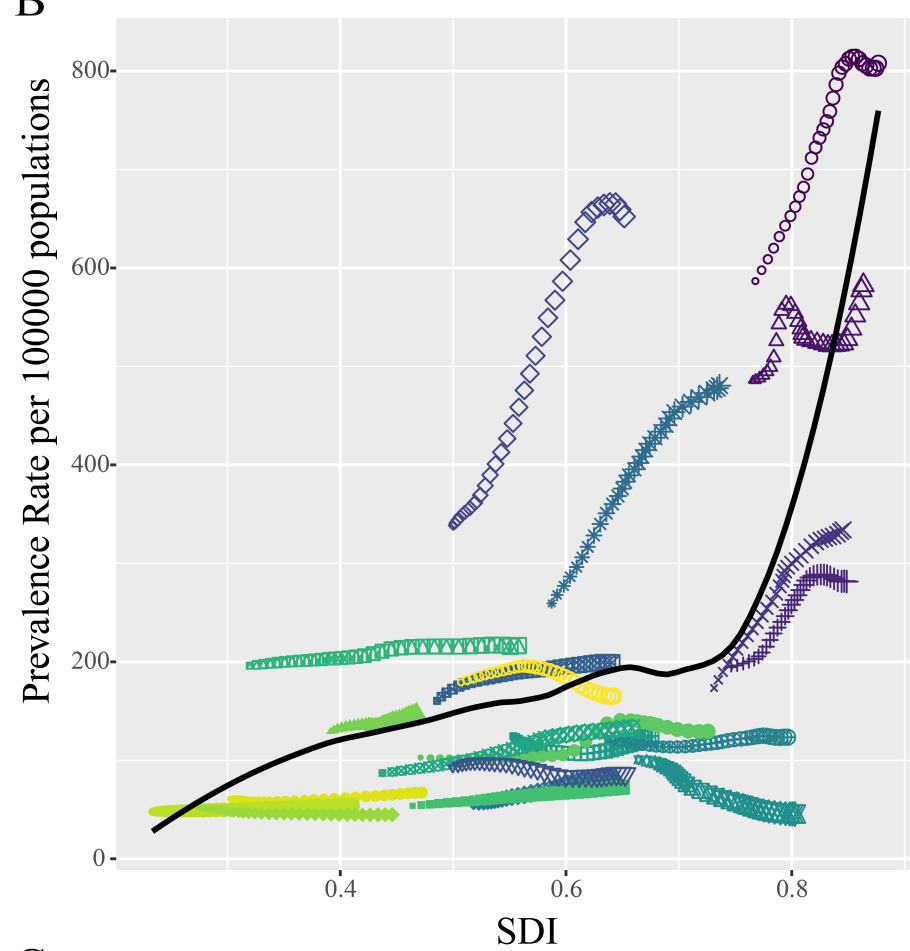

C

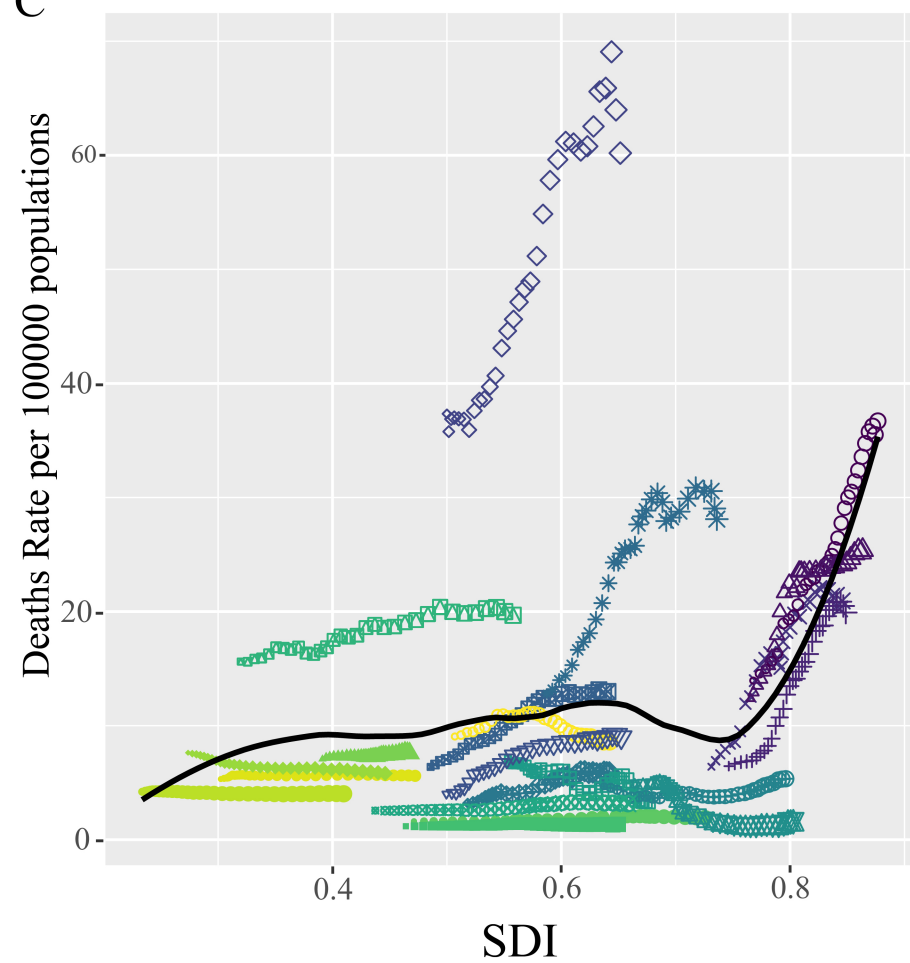

Supplement: S5 Fig — Abbreviations: SDI, Sociodemographic Index. (PDF) [file pone.0347482.s012.pdf]

A

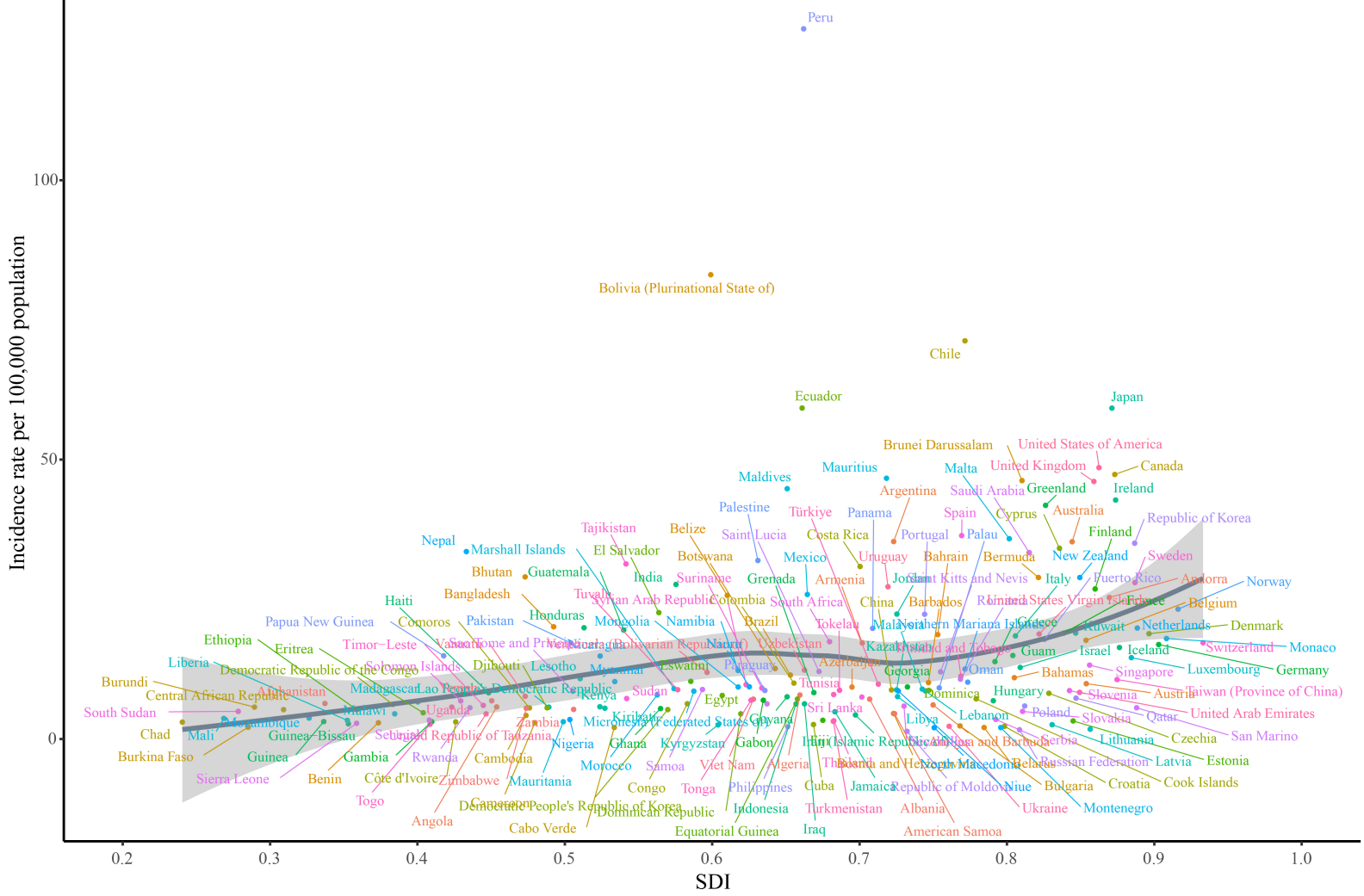

B

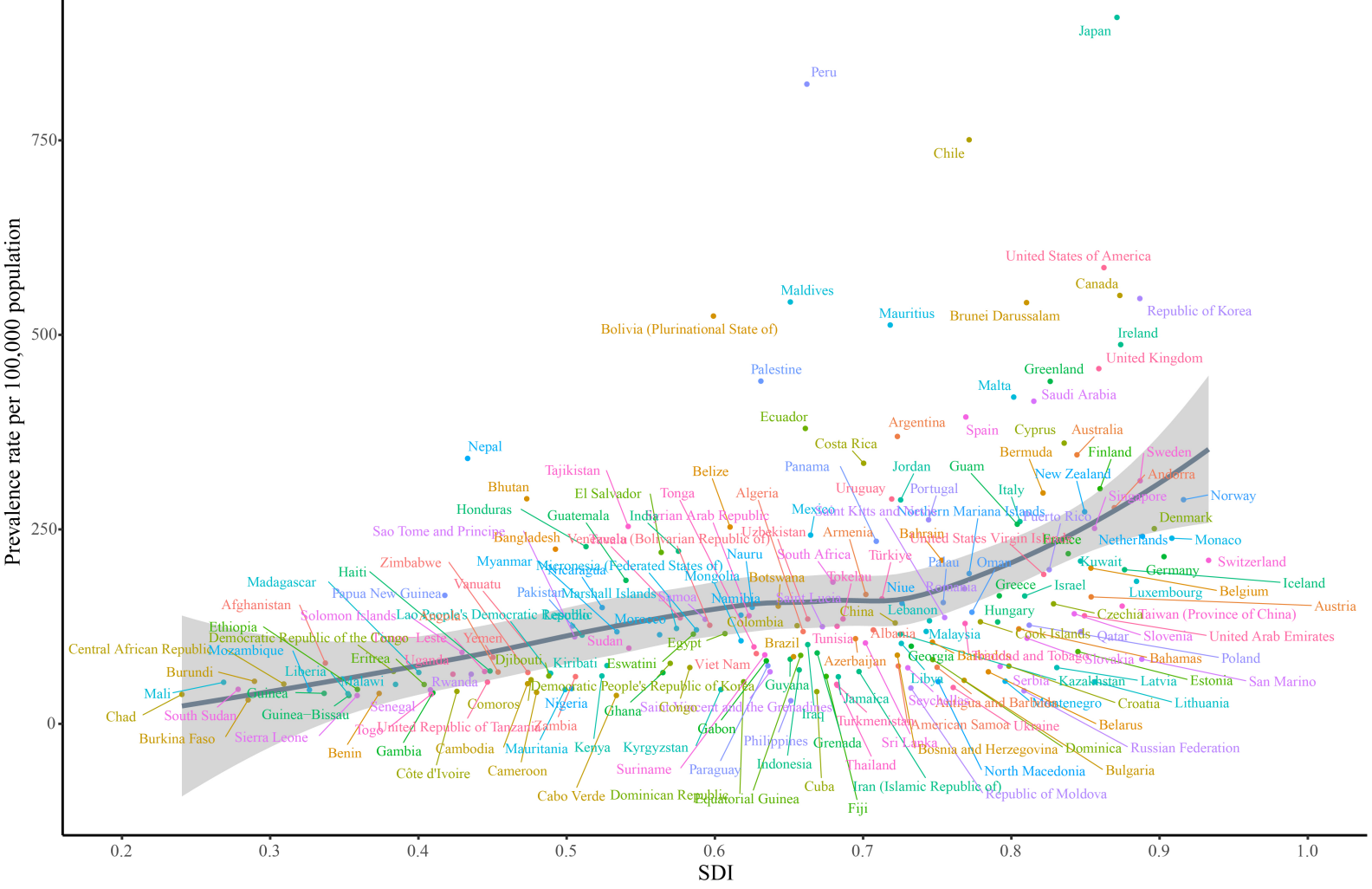

C

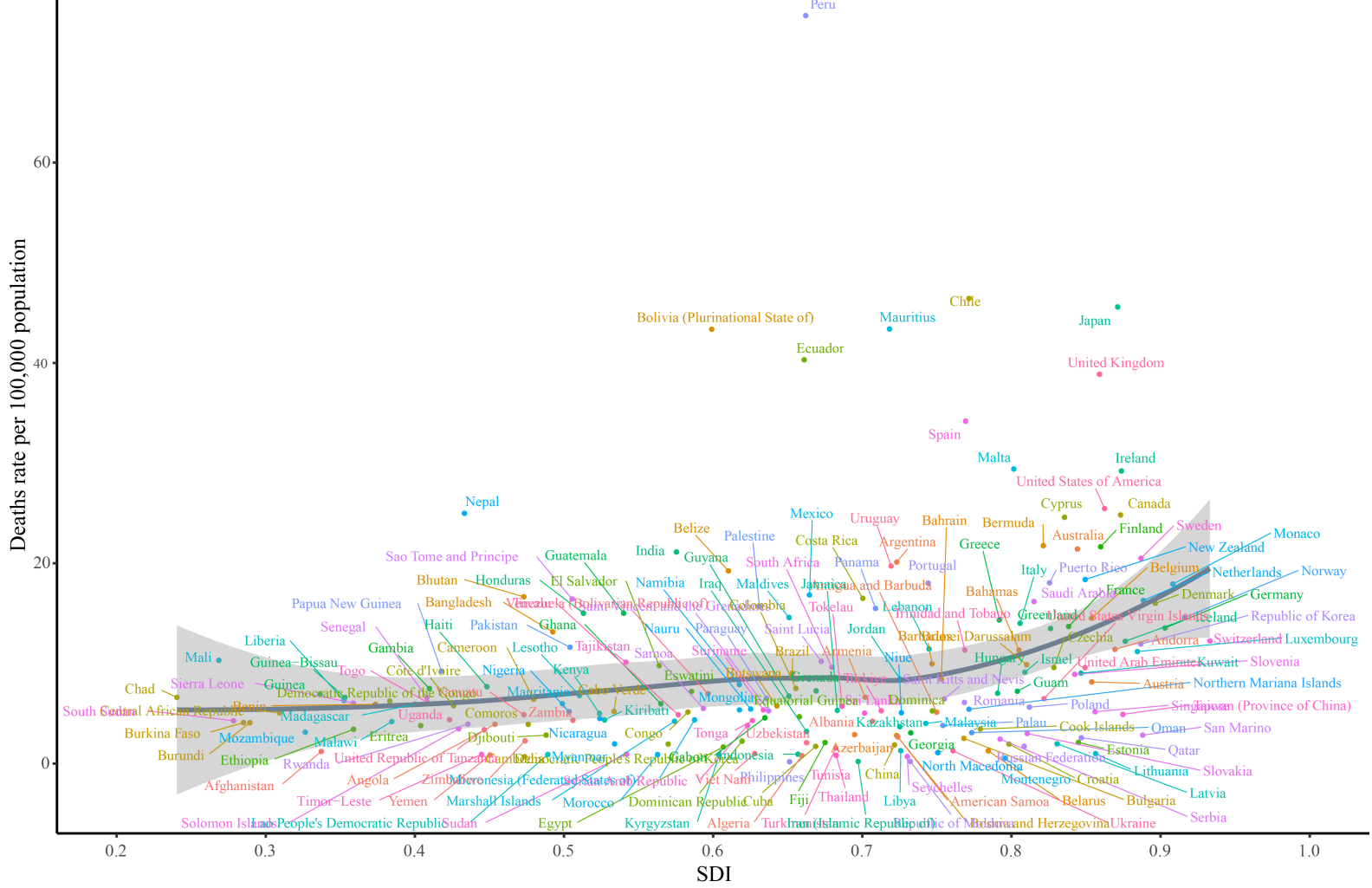

Supplement: S6 Fig — Abbreviations: SDI, Sociodemographic Index. (PDF) [file pone.0347482.s013.pdf]

A

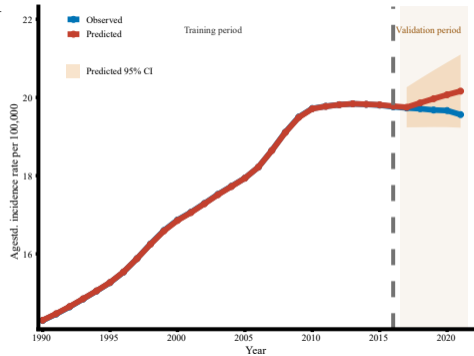

B

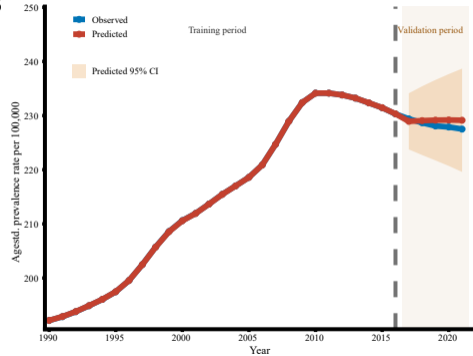

C

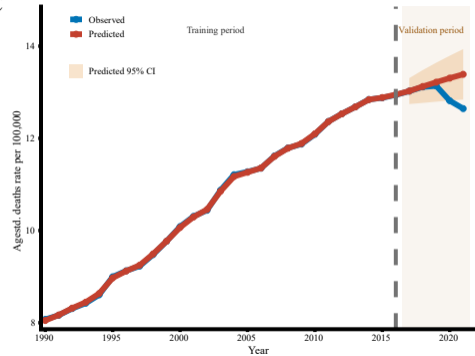

Supplement: S7 Fig — (PDF) [file pone.0347482.s014.pdf]
